# Opportunities lost: Barriers to increasing the use of effective contraception in the Philippines

**DOI:** 10.1371/journal.pone.0218187

**Published:** 2019-07-25

**Authors:** Mari Nagai, Saverio Bellizzi, John Murray, Jacqueline Kitong, Esperanza I. Cabral, Howard L. Sobel

**Affiliations:** 1 Bureau of International Health Cooperation, National Center for Global Health and Medicine, Tokyo, Japan; 2 Partnership for Maternal, Newborn and Child Health, Geneva, Switzerland; 3 Independent consultant, maternal and child health, Iowa City, United States of America; 4 World Health Organization Philippines Country Office, Manila, Philippines; 5 Responsible Parenthood and Reproductive Health National Implementation Team (RP-RH NIT), Department of Health, Manila, Philippines; 6 Division of NCD and Health through Life-Course, Reproductive, Maternal, Newborn, Child and Adolescent Health, World Health Organization Regional Office of the Western Pacific, Manila, Philippines; University of Washington, UNITED STATES

## Abstract

**Background:**

In the Philippines, one in four pregnancies are unintended and 610 000 unsafe abortions are performed each year. This study explored the association between missed opportunities to provide family planning counseling, quality of counseling and its impact on utilization of effective contraception in the Philippines.

**Methods:**

One-hundred-one nationally representative health facilities were randomly selected from five levels of the health system. Sexually-active women 18–49 years old, wanting to delay or limit childbearing, attending primary care clinics between April 24 and August 8, 2017 were included. Data on contraceptive use, counseling and availability were collected using interviews and facility assessments. Effective contraceptive methods were defined as those with rates of unintended pregnancy of less than 10 per 100 women in first year of typical use.

**Findings:**

849 women were recruited of whom 51.1% currently used effective contraceptive methods, 20.6% were former effective method users and 28.3% had never used an effective method. Of 1664 cumulative clinic visits reported by women in the previous year, 72.6% had a missed opportunity to receive family planning counseling at any visit regardless of level of facility, with 83.7% having a missed counseling opportunity on the day of the interview. Most women (55.9%) reported health concerns about modern contraception, with 2.9% receiving counseling addressing their concerns. Only 0.6% of former users and 2.1% never-users said they would consider starting a modern contraceptive in the future. Short and long acting reversible contraceptive methods were available in 93% and 68% of facilities respectively.

**Conclusions:**

Missed opportunities to provide family planning counseling are widespread in the Philippines. Delivery of effective contraceptive methods requires that wider legal, policy, social, cultural, and structural barriers are addressed, coupled with systems approaches for improving availability and quality of counseling at all primary health care contacts.

## Introduction

Unintended pregnancies remain an important public health problem worldwide. Between 2010 and 2014, there were an estimated 62 unintended pregnancies per 1000 women aged 15–44 years each year, with rates ranging from 112 in East Africa to 28 in Western Europe [1]. In Philippines, 54% of all pregnancies (1.9 million pregnancies) are reported to be unintended and around 610 000 unsafe abortions are performed each year [2]. Nine percent of women 15 to 19 years of age have begun child bearing [3].

In 2017, the modern contraceptive prevalence rate (CPR) in the Philippines was estimated to be 40% among married women of reproductive age and 17% among unmarried sexually active women [3]. The modern CPR increased only 2% between 2013 and 2017, with rates being much lower in some populations. Forty-six percent of married women used no contraceptive method in 2017 and 14% a traditional method, a decline from 16.7% in 2008; only 10% of women used long acting reversible contraceptives (LARCs) such as IUDs and implants [3]. Among sexually active unmarried women, traditional methods were used by 15% [3].

Women who are fecund, sexually active and who want no more children or to delay the next child, but are not using any method of contraception, are defined as having an “unmet need for family planning”. The unmet need for family planning among married women of reproductive age in Philippines was 17% in 2017, with the demand met by modern methods estimated to be 57%. Among unmarried sexually active women the unmet need increases to 49% and the demand met by modern methods falls to 22% [3]. As a consequence of the low contraceptive met need, 68% of unintended pregnancies occur in women not using any method and 24% in those using traditional methods [4]. Those using LARCs rarely have unintended pregnancies [5].

Several barriers to accessing family planning services have been observed in the Philippines. A 2013 survey found that maintenance of virginity until marriage was important for 83% of women aged 15–24, even though 14% of 15–19 year-olds and 49% of those aged 20–24 years experienced first sexual intercourse before marriage [6]. This social norm paradoxically discourages use of contraception by unmarried women. In addition, infrequent sex was commonly stated as a reason why women with an unintended pregnancy did not use modern methods. Among married women with an unmet need, half cite inappropriate health concerns as a reason not using modern methods including weight loss, chemical toxicity due to prolonged use, excessive bleeding, the buildup of blood if menstruation stops, loss of physical strength, debilitating headaches or stomach aches, and fears that devices that get lost inside the body [7]. Religion-based opposition to contraception was reported as a barrier by only 3%-6% of women and accessibility of methods by 2% to 7% [7].

Although the family planning program in the Philippines began in 1971 and was one of the strongest in Asia, religious concerns, rapid decentralization and various legal interventions have restricted implementation. The Responsible Parenthood and Reproductive Health Act of 2012, designed to re-vitalize family planning service provision, was not put into place until 2017 when legal and programmatic barriers had been overcome [8].

In response, the Department of Health in Philippines sought to identify strategies to improve family planning programming. Improvement of uptake of modern methods of contraception, especially LARCs, became an important public health priority. Experience from other countries in Asia has shown that facility-based contraceptive counseling is often poor [9, 10]. Women are often dissatisfied with clinic visits, because they are unable to discuss their concerns and receive insufficient information about their options. Additionally, providers frequently have inaccurate knowledge about contraceptive methods, including out-of-date information [11, 12, 13, 14]. For these reasons, this study was designed to review current counseling practices as a key barrier to uptake of contraception. Since the government health insurance provider (Philhealth) provides coverage for 66% of the population, with 56% of women currently obtaining contraception at public health facilities [3], the study was designed to focus on practices in the public sector. The objectives were to identify the extent of missed opportunities to provide family planning counselling at primary care visits, whether effective counselling was provided and its impact on women’s concerns and decision making to begin use of modern contraceptives. The goal was to identify systems factors that could be targeted to improve the quality of care and to reduce unintended pregnancies.

## Methods

### Study design

A nationally representative cross-sectional survey design was used. One hundred one health facilities (11 national hospitals, 13 regional hospitals, 23 provincial hospitals, 27 main health centers, and 27 barangay health centers) were selected. In first stage sampling, 23 provinces were randomly selected using probability proportionate to size based on the estimated number of sexually active women with unmet need [15]. Within the sampled provinces, the provincial hospital, 1 main health center and 1 Barangay health center of the most populated Barangay were selected. Regional and national hospitals within the province were also included. The number of facilities sampled was based on estimated participants sample size (n = 820) required to allow comparisons of the proportion women using modern methods of contraception by socio-economic factors with 80% power.

At each facility, women aged 18–49 years of age, who were not currently pregnant or within 6 weeks of delivery, wanting to delay or limit childbearing on the day of the visit were eligible to for the study, to avoid confounding by stage of pregnancy, place of delivery or the early post-pregnancy period on the likelihood and quality of counseling. Questions about missed opportunities to provide family planning counseling were asked about all clinic visits in the previous year. Individual interviews using structured questionnaires were conducted with 10 women per hospital and 6 per health center. Sampled women were divided into equal groups of those currently using and not using effective contraceptive methods. We defined effective contraceptive methods as those with rates of unintended pregnancy of less than 10 per 100 women in first year of typical use (i.e., patch, oral pills, injectables, IUDs, implants and sterilization) [5]. We excluded lactational amenorrhea to focus on non-transient modern methods. Less effective contraceptive methods were defined as condoms, fertility self-monitoring (i.e, standard days method, basal body temperature) and traditional methods (i.e., withdrawal, calendar or rhythm method). Where multiple methods were used, subjects were categorized according to the most effective method used.

Data were collected from women attending the outpatient primary care clinic on the day of the survey. Primary care clinics included postnatal care, reproductive health, well child, and primary care. If more than the required number of women were in attendance, systematic sampling method was used. At five sampled health facilities, the minimum number of women were not available on the day of the survey. At these facilities, 24 (2.8%) women meeting eligibility requirements for the study and living within 1 hour of the facility were identified from clinic registers for the previous days and interviewed at their homes to obtain the minimum sample size.

A closed-ended questionnaire for women, interview guide and health facility assessment checklist were developed referring to previous research. These were finalized after two field pilot tests. To those women who preferred local dialects, the questionnaires and consent forms were translated into one of eight local dialects (Tagalog, Ilocano, Bicolano, Visayan, Ilonggo, Chavacano, Tausug, and Meranao) then validated by back-translation. Eight teams consisting of one team leader and one enumerator collected data from April 24 to August 8, 2017 using paper-based questionnaires. Enumerators near selected facilities were identified to minimize language differences across study sites. Data were collected using: 1) interviews with women on current, former and never use of effective contraceptive methods, concerns and attitudes towards contraception, and management of concerns at outpatient primary care clinics of health facilities; 2) facility assessments of the availability of family planning commodities and policies including hospital accreditation status for use of LARCs.

### Study outcomes

Counseling was defined as any information or advice given about any contraceptive method during the woman's clinic visit. A missed opportunity was defined as a woman’s clinic visit at which a staff member at the health facility did not provide any counseling. When counseling was provided, quality of counseling was measured by asking the woman whether health staff asked about her concerns on family planning, helped her to find solutions to her concerns, offered her information about how different family planning methods work, or explained side-effects or problems she might have with any method. A health concern about a family planning method was defined as any perceived undesirable health effect. Accumulated facility visits were defined as the number of visits to health facilities made by interviewed women between January 1 and December 31, 2016. Accumulated missed opportunities were defined as the total number of accumulated facility visits at which health staff did not provide any family planning counseling on the day of that visit.

### Data management and analysis

The team leader checked completeness and accuracy of completed paper-based questionnaires on the day of interview. Two data managers independently entered the questionnaire data into identical Excel sheets and compared using STATA version 13.1. Inconsistencies were validated with the original paper and corrected. Statistical analysis was also done using STATA.

### Ethical considerations

Ethical clearance was obtained by the WHO Regional Office for the Western Pacific Ethics Review Committee on 12 January 2017, and the National Ethics Committee in the Philippine Council for Health Research and Development of the Department of Science and Technology on 1 February 2017. All women were asked for informed consent. Participants were informed of their right to refuse participation in the study, or not answer specific questions should they assent to participation and were assured of the confidentiality of the collected information. All interviews were conducted in private settings and unique identifiers were used to maintain anonymity. All paper copies are maintained in a locked file cabinet.

## Results

**Fig 1:**
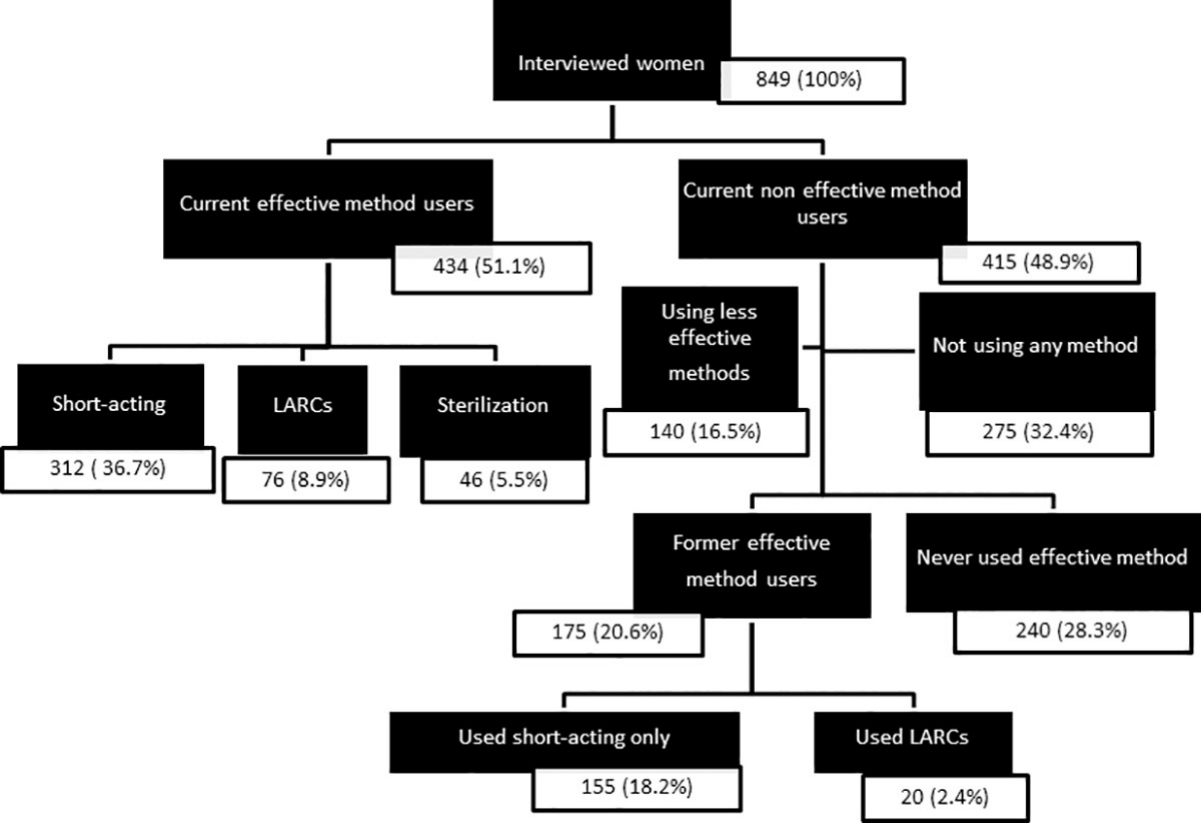
Status of contraceptive use among 849 women 18–49 years of age wanting to delay or limit childbearing, outpatient care clinics, Philippines, 2017.

A total of 849 non-pregnant sexually active women, 18–49 years of age, wanting to delay or limit childbearing were included in the study. Only one woman meeting the entry criteria refused to be interviewed. Of sampled women, 51.1% (434) were currently using an effective contraceptive method, 16.5% (140) a less effective contraceptive method and 32.4% (275) no contraceptive method. Of all women, 36.7% (312) were currently using short-acting effective methods (pills, patches or injectables), 8.9% (76) LARCs (IUD or implants) and 5.5% (46) female sterilization. An additional 18.2% (155/849) of women had used short-acting effective methods in the past and 2.4% (20) had used LARCs in the past. An effective method had never been used by 28.3% (240) of women (Fig 1).

Demographic characteristics of sampled current, former and never users were similar (Table 1). Respondents were predominantly urban, aged 30 years, married or living together, with at least a high school education and 2 living children. They reported an average of 1.9 health facility visits in the past year. Philhealth insurance coverage in our sample was 71.3% with 24.7% having no health insurance. Women who had never used contraception were more likely than current users to have not graduated high school.

**Table 1:** Characteristics of users, former users and never users of effective contraceptive methods for women of reproductive age who are not currently pregnant or within 6 weeks of delivery and want to delay or limit childbearing, outpatient care clinics, Philippines, 2017.

Characteristic	Current users	Former users	Never users	Total
**Total women, N (%)**	434 (51.1)	175 (20.6)	240 (28.3)	849 (100)
**Residence, n (%)**				
Urban	328 (75.6)	118 (67.4)	180 (75.0)	626 (73.7)
Rural	106 (24.4)	57 (32.6)	60 (25.0)	223 (26.3)
**Age, mean (standard error)**	30.7 (7.2)	32.9 (7.5)	29.8 (7.3)	30.9 (7.4)
**Marital status, n (%)**				
Never married	2 (0.4)	3 (1.7)	6 (2.5)	11 (1.3)
Married	258 (59.5)	114 (65.1)	141 (58.7)	513 (60.4)
Living together	167 (38.5)	48 (27.4)	79 (32.9)	294 (34.6)
Divorced/Separated/Widow	7 (1.6)	10 (5.8)	14 (5.9)	31 (3.7)
**Education**^**1**^**, n (%)**				
No Education/ Elementary	44 (10.1)	21 (12.0)	33 (13.7)*	98 (11.5)
High school	250 (57.6)	92 (52.6)	106 (44.2)	448 (52.8)
More than high school^2^	140 (32.2)	62 (35.4)	101 (42.1)	303 (35.7)
**Number of living children, n (%)**				
0	0 (-)	0 (-)	2 (0.9)	2 (0.2)
1	77 (17.7)	28 (16.0)	75 (31.2)	180 (21.2)
2–3	234 (53.9)	91 (52.0)	105 (43.7)	430 (50.7)
4+	123 (28.4)	56 (32.0)	58 (24.2)	237 (27.9)
**Type of visit**				
Postnatal care	95 (58.3)	252 (59.1)	151 (58.1)	498 (58.6)
Reproductive health care	56 (34.3)	152 (35.7)	100 (38.5)	308 (36.3)
Well child care	11 (6.7)	19 (4.5)	6 (2.2)	36 (4.2)
Primary care for herself	1 (0.7)	3 (0.7)	3 (1.2)	7 (0.9)
**Health facility visits in the 12 months preceding the survey, mean (SE)**	1.9 (1.1)	1.9 (1.0)	1.8 (1.3)	1.9 (1.1)
**Financial protection, n (%)**				
None	112 (24.6)	39 (22.3)	59 (24.6)	210 (24.7)
PhilHealth	305 (71.5)	126 (72.0)	174 (72.5)	605 (71.3)
Other health insurance^3^	17 (3.8)	10 (5.7)	7 (2.9)	34 (4.0)

* Statistically different (p < .05) compared with current users

^**1**^ Education categories refer to the highest level of education attended, whether or not that level was completed

^2^ Including college, post-graduate or vocational training.

^3^ Government service insurance system, social security system, private insurance, health maintenance organization, pre-need insurance plan.

**Fig 2:**
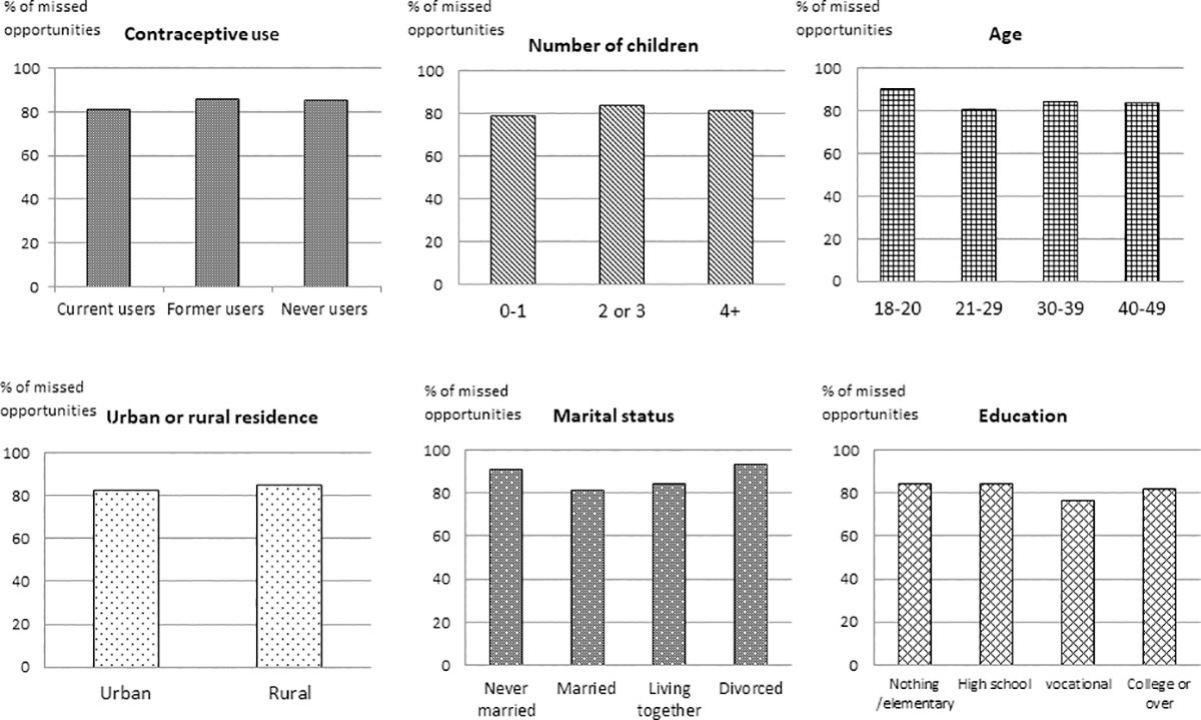
Missed Opportunities to provide family planning counseling on the day of the interview, by women’s characteristic (%), outpatient care clinics, Philippines, 2017.

Of all women, 83.7% (711/849) reported a missed opportunity for family planning counseling on the day of the interview. Missed opportunities were high regardless of contraceptive method used, residence, age group, marital status, education, number of children, or financial protection status, but highest among those younger than 21 years old, and women living together or not married (Fig 2). Missed opportunities remained high across all levels of health facility and facility accreditation or certification status.

**Fig 3:**
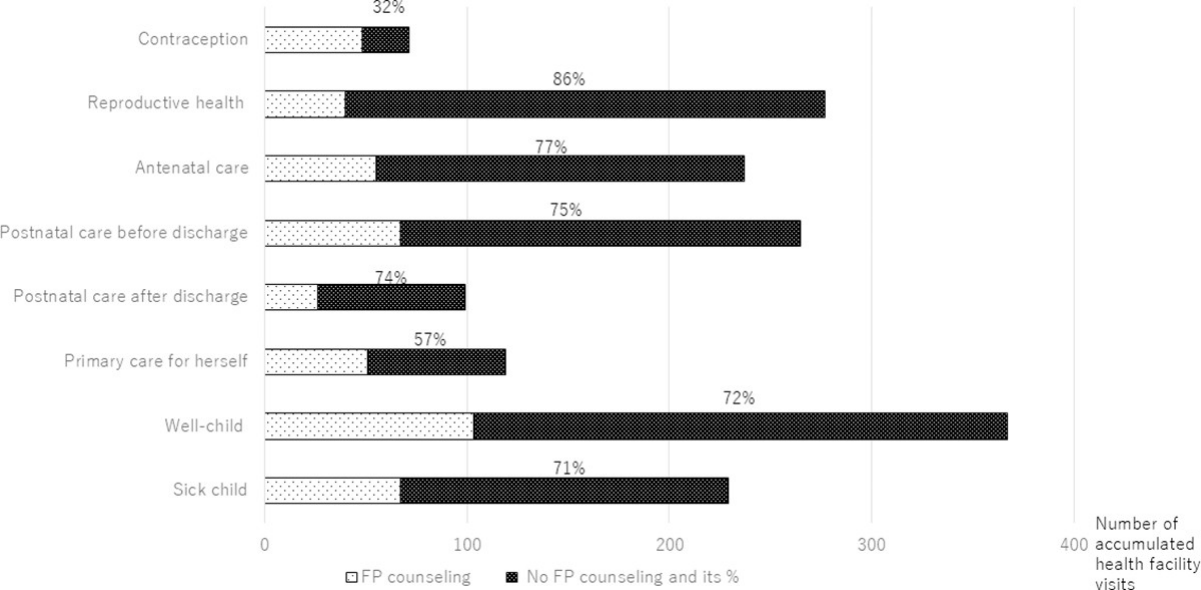
Cumulative number of visits and missed opportunities for family planning counseling by type of outpatient care clinic visit, Philippines, January 1 2017 –December 31, 2017.

The 849 women interviewed reported 1664 accumulated primary health care facility visits including antenatal care and postnatal care before discharge, between January 1 and December 31, 2016, not including the day of the interview. Of the 1664 accumulated health facility visits, 72.6% (1211) were missed opportunities to provide family planning counseling. Missed opportunities were found for 68.6% (587/856) of visits by current effective contraceptive method users, 73.4% (235/320) by former users and 78.9% (385/488) by never users. Overall, women not currently using any effective contraceptive method had a missed opportunity in 76.7% (620/808) of all clinic visits in 2016. Missed opportunities to provide family planning counseling were reported for 85.8% (241/281) of reproductive health clinic visits, 76.8% (182/237) of antenatal care visits, 74.7% (198/265) of postnatal care before discharge contacts, 73.7% (73/99) of postnatal care visits after discharge, 71.9% (264/367) of well-child visits, 70.7% (162/229) of sick child visits and 57.1% (68/119) of primary care visits for herself. Missed opportunities were lowest for contraceptive clinic visits, at which 32.8% (23/70) women reported that family planning counseling about any method was not provided (Fig 3).

Sixteen percent (138/849) of women received family planning counseling on the day of the interview. Of the 138 women receiving counseling, 67.4% (93) were asked if they had concerns about contraceptives, 59.4% (82) received information about how different contraceptives work and 44.9% (62) were told about possible contraceptive side-effects during the counseling. Current effective contraceptive users were more likely to receive information than former or never users. Of the 80 current users receiving family planning counseling, 75.0% (60) were asked about concerns, 66.3% (53) were given explanations about how different methods work, and 55.0% (44) were given advice about side-effects. In contrast, the same information was given less frequently to the 24 former users receiving counseling (50%, 41.6% and 37.5%, respectively) and the 34 never users receiving counseling (62.9%, 55.9% and 23.5%, respectively).

Overall, 55.9% (481/849) of women reported a total of 567 concerns about any effective contraceptive method on the day of the interview. The prevalence of concerns was 72.6% (126/175) among former users, 56.0% (242/434) among current users, and 47.5% (113/240) among never users. Only two women reported concerns that were not related to health: one woman who had never used effective contraception stated that it was illegal to use sterilization and IUDs; and another currently using injectables stated that the church did not allow the use contraception. Of all 567 concerns, 64.9% (368) were medically known side effects such as changes in bleeding patterns, irritability (mood changes), headaches and weight gain^4^, while 35.1% (199) were misperceptions. The latter included the belief that contraceptives caused uterine cancer, cysts, fetal malformations, varicose veins, and dry skin. Misperceptions accounted for 33.6% (38/113) of all concerns among current, 25.7% (44/171) former and 41.3% (117/ 283) never users. Misperceptions were particularly high about female sterilization (87.0% or 20/23 of concerns such as frequent bleeding or cause uterine cancer) and IUDs (67.6% or 50/74 of concerns were misperceptions such as movement to other organs inside the body). Misperceptions were a low proportion of concerns for implants (37.5% or 9/24 of concerns), pill (27.9% or 79/283 of concerns) and injectables (25.1% or 41/163 of concerns).

**Fig 4:**
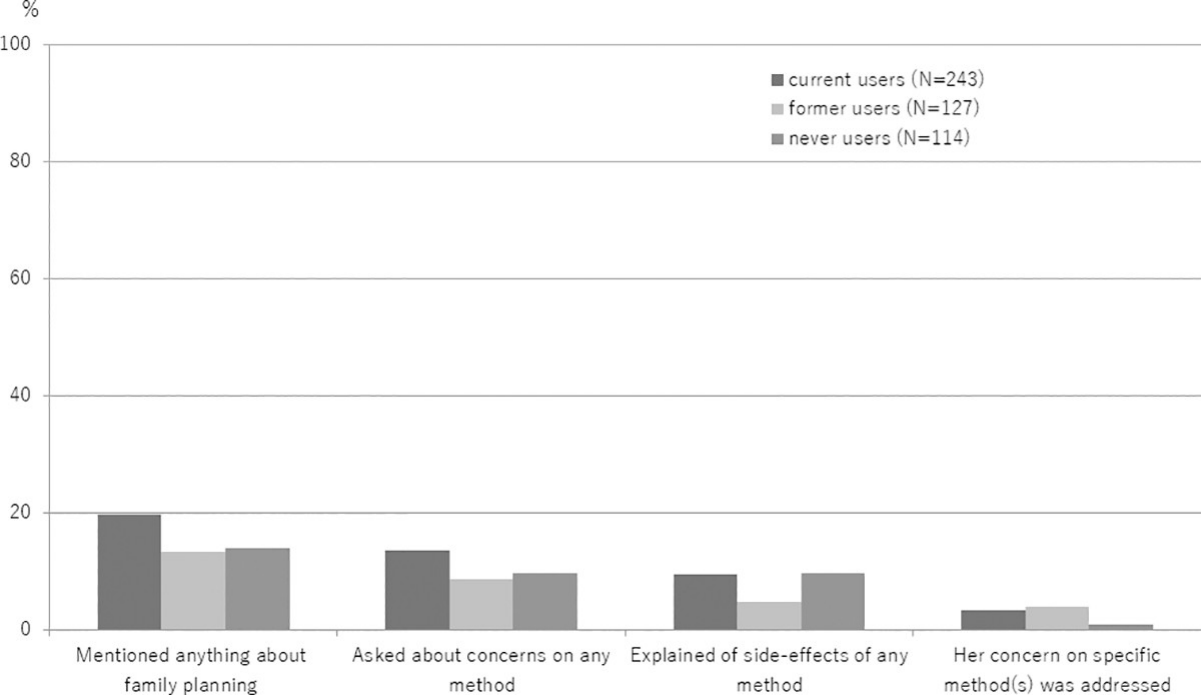
Family planning counseling received by women with health concerns about contraception on the day of the interview at outpatient care clinics, Philippines, 2017.

Of the 481 women with a health concern about effective contraceptive methods, 16.3% (79/481) received family planning counseling on the day of interview. Regardless of contraceptive usage status, information given to women with concerns was limited, with only 2.9% (14/481) of women reporting that their concern about a specific method(s) had been addressed by the health worker during counseling (Fig 4). Only 0.6% (1/175) of former users and 2.1% (5/240) never users with concerns said they would consider starting a modern contraceptive method at the time of the interview.

A stock of non-expired effective short-acting contraceptive methods was available at 87.5% (42/47) of government hospitals, 95.7 (45/47) of health centers, and 100% (7/7) of barangay health stations. LARCs were available in 85.1% (40/47) of government hospitals, 60% (28/47) (61%) of health centers and 1/7 (14.3%) of barangay health stations.

## Discussion

Of 1664 cumulative total clinic visits reported by the women wanting to delay or limit childbearing in 2016, 72.6% had a missed opportunity to receive family planning counseling at any visit regardless of level of facility or socio-economic indicators. On the day of the interview in 2017, 83.7% of women had a missed opportunity. Although 55.9% of women reported health concerns about effective contraceptives on the day of the interview, 16% with a concern received family planning counseling and only 2.9% received counseling addressing her specific concerns. Only 0.6% of former effective contraceptive method users and 2.1% never-users said they would consider starting effective contraceptive method in the future. As relatively few facilities had stock-outs, most women in our study could have received an effective contraceptive on the day of the visit, if they had received high quality of counseling, been offered a method and had decided to use it.

These findings suggest that the quantity and quality of family planning counseling provided at primary care clinic contacts in Philippines to women who wish to delay or limit childbearing is inadequate and unlikely to significantly increase the use of effective contraceptive methods. Around 20% of women attending clinics in this study had previously used an effective contraceptive method, but had discontinued use, highlighting that contraceptive services must focus not only on attracting new users but also on improving continuation rates [16]. To do this, current users should receive continued counseling at every contact, to address the emerging or ongoing concerns about methods; and past users who have stopped use targeted, where appropriate, to resume use by identifying reasons for discontinuation.

The high prevalence of missed opportunities to provide family planning counseling found in this study is consistent with other studies in both developed and developing country settings [17–19].

The World Health Organization recommends providing routine family planning counseling at antenatal care, postnatal care, and other contact points [20–23]. However, experience with integration of family planning counseling into routine primary care is mixed. Significant improvements in family planning outcomes have been seen when improved counseling or referral is integrated into both immunization clinics and general primary care clinic settings [24–26]. In other settings limited improvements in contraceptive use are seen with integration into other services [27–30]. These studies suggest that integrating family planning counseling into routine practice cannot be effective unless systems barriers at primary care clinics are addressed, including the availability of staff, patient numbers and flow, space for adequate one-on-one counseling and availability of low-risk contraceptive methods.

Client-centered counseling approaches are associated with improved method continuation. Women who report experiencing higher quality care have higher rates of contraceptive continuation and contraceptive use [31–33]. In reality, interactions between clients and providers are often provider-dominated, with minimal engagement with women in the process of method selection and with frequent failure of providers to deliver personalized counseling tailored to the individual women's needs and preferences [34–36].

Most counseling in primary care clinics in Philippines is provided by doctors, nurses and midwives. Although family planning counseling at both antenatal and postnatal care contacts is included in national policies and guidelines in Philippines [37], our findings indicate they are rarely translated into practice. Improving delivery of effective contraceptive methods requires addressing wider legal, policy, social, cultural, and structural barriers which prevent individuals from accessing and using contraception and influence the quality of counseling provided. Some recent policy initiatives in Philippines may promote improved family planning counseling. The Universal Health Coverage Act (RA11223) signed into law in Philippines in February 2019, requires that FP counseling should offered at all primary care contacts, along with a package of essential services. The Expanded Maternity Leave Act (RA11210) also signed in February 2019, increases paid maternity leave from 60 to 105 days and is designed to improve opportunities for postpartum care, including improve family planning counseling and method provision. In 2012, the Responsible Parenthood and Reproductive Health Act (RPRHA) passed, which guarantees universal and free access to modern contraceptives, in particular for poor women [8].

However, the RPRHA imposes a ban on the purchase of dedicated emergency contraceptives by national hospitals, and requires parental consent for minors to access contraceptives. These restrictions directly impose barriers to contraceptive use on poorer women and adolescent girls [38].

Although many Philippine-governmental norms and standards are in agreement with adolescents’ human rights to contraceptive information and services recommended by the WHO, a significant number are restrictive, reflecting the strong influence of conservative religious beliefs [39]. In addition, decentralization of the health system, gives local chief executives the power to ignore national health policies and programs. For example, the mayor of Manila banned contraceptive services in local health facilities in 2000 because of his own religious objections [40]. In Philippines, staff report that providers who are members of the church often face pressure not to distribute contraception and sometimes pressure from anti-reproductive health groups. Religious beliefs are not cited as important barriers among women of reproductive age, however, suggesting that if appropriate information and counseling was provided, many women would consider adopting modern methods.

There are also a number of provider perceptions that may limit counseling provision, including lack of knowledge, training, and comfort, assumptions about patient pregnancy risk, negative beliefs about contraceptive methods, a reliance on patients to initiate discussions; and limited communication with other primary care staff [41, 42, 43]. Our finding of 32% missed opportunities at contraception clinics suggests that provider-related perceptions and skills continue to play a role in limiting the quality of counseling in the Philippines.

Social norms are believed to be particularly important barriers in Philippines. These norms prevent women, especially adolescents and unmarried women, from accessing services and using methods effectively. The high value placed on virginity at marriage discourages women from admitting sexual activity and inappropriate health concerns [6].Women frequently do not use any contraceptive method, despite wanting to avoid pregnancy, because they do not perceive themselves to be at risk of pregnancy or they have concerns about the methods, perceptions that are reinforced by families and communities where they live [44]. In some cases, health-provider or community assumptions about needs may conflict with the women’s own assessment, especially in contexts where there has been a history of contraceptive coercion or discrimination. Providers often offer less-effective methods such as condoms to adolescents believing that LARCs are inappropriate for women who have never had a child. This is despite the fact there is no medical reason to withhold LARCs from adolescents and young women [45].

This study confirms that LARCs such as IUDs and implants are used less frequently than short acting methods in the Philippines despite being far more effective [5]. Although previous studies have shown that cost and availability are minor contributors to stoppage or non-use of family planning in general, long-acting methods require specially trained workers and upfront payments which may present barriers to use [46]. In the Philippines, the national policy allowing only accredited facilities, mostly hospitals, to administrator LARCs results in lower availability at peripheral level facilities. Alternative models, which provide on-site family planning counseling and services, have been shown to increase uptake of LARCs [23].

In summary, improving family planning counseling at all clinic contacts in the Philippines will require actions at several levels. Recognizing the role of effective contraception to improve the health and economic development of the country is the first step and should be reflected in national laws and policies. Legal barriers such as limitations on the availability of emergency contraception and requiring parental consent can be carefully re-considered and removed. National policies and guidelines should be reinforced at all levels to ensure they are consistent with WHO recommendations and to remove abstinence-centered and sex-negative content. Mechanisms to avoid local adaptations which bypass national policies and guidelines should be explored. Availability of LARCs can be increased by expanding the range of facilities where they can be provided and the number and categories of staff trained in their use. National policies, guidelines and standard operating procedures for primary care services can emphasize integrating family planning counseling at all contacts. Health worker awareness and counseling skills can be strengthened by incorporating skills into professional medical, nursing and midwifery pre-service training curricula and by providing on-the-job coaching of doctors, nurses and midwives; client-centered counseling approaches can address both relational and task-based communication focusing on common obstacles to use [47]. Development of simple screening tools may allow many counseling tasks to be done by under-employed staff at facilities, removing pressure on those providing clinical services. This approach may be particularly useful for screening current users about concerns; and for identifying and improving support for past users who have stopped use. Systems barriers to counseling need to be identified and addressed in all primary care settings. These include organization of clinic space, patient flow and time to allow counseling to take place, making adequate human resources available, task shifting to improve quality and making LARCs more widely available in primary care settings. Facility accreditation, professional licensure and performance-based financing can help to maintain the motivation of health professionals and provide incentives for prioritizing uptake of effective contraceptive methods.

This study aimed to select a nationally representative sample, based on unmet need for family planning. Since the 2014 Philippine Demographic and Health Survey calculated population-based estimates of unmet need only at the regional level, provinces within a region with higher than average unmet need may be under-represented [15]. Philhealth insurance coverage in the study population was 71.3% compared to 65.8%, in the general population; 24.7% of the sample had no health insurance compared to 9.9% in the general population [3]. Higher uninsured rates suggest that the study population may be poorer and less educated, which puts them at higher risk of not using modern methods of contraception. The sample was not weighted for different outpatient clinic attendance rates, which may differ by clinic level and could not be determined in advance. At the clinic level women were selected by order of attendance until the required number was obtained; if more than the required number was present, they were selected by systematic random sampling ordered by time of arrival. In 24 cases home visits were made to interview women who had attended on the previous day. If the characteristics of women or of counseling provided differed by time of attendance, then the sampling of women at clinics could introduce bias. In addition, the study could not control for the clinic type from which women were sampled, since various clinics often fell on certain days of the week. To mitigate these limitations of sampling, we also analyzed the data from the accumulated visits of interviewed women for previous 12 months. The universal presence of missed opportunities across all clinic types at all levels, and the consistency of findings by demographic characteristics, suggests that these factors are unlikely to produce significant biases.

Since our sample was facility-based, findings may not reflect the practices of those who have less access to public clinics or seek care less regularly. In addition, adolescents under 18 years of age were excluded, because their inclusion would have required a lengthy institutional review process which could not be completed within financial deadlines. Given the high missed opportunities among adult women, omission of these groups likely meant that our findings underestimate the extent of the problem.

We asked respondents about family planning services received on the day of the interview and at all clinic visits in 2016. A maximum period of 6 months has been suggested for the recall of non-significant events [48]. However, rates of missed opportunities did not change significantly between the day of interview and one-year accumulated visits across all demographic categories. To limit the influence of recall bias, detailed data on quality of counseling were collected only for counseling conducted on the day of interview.

## Conclusions and broader implications

As in other low- and middle-income countries, the Philippines faces substantial barriers to improving reproductive health services. This study showed that the vast majority of women who wish to delay pregnancy, attending public health clinics, did not receive family planning counseling regardless of their current status of contraceptive use or clinic attended. Over half of these women had health concerns about effective contraceptive methods which were not addressed. This striking finding means that many women in contact with the health system continue to use no contraception, use traditional and other less-effective methods or stop using contraception altogether. As a consequence, many women are put at unnecessary risk of short-spaced high-risk pregnancies and cycles of high fertility, lower educational and employment potential and poverty [49–51]. The study shows that effective contraceptive methods are generally available and that out-of-pocket expenditure was not a major barrier. Systems approaches for improving availability and quality of contraceptive counseling at all primary health care contacts are now needed. Since similar systems problems exist across low and middle-income countries, better quantification of missed opportunities to provide contraceptive counseling and barriers to use is an urgent priority [52].

## Supporting information

S1 Questionnaire Questionnaire to women in English. (PDF)

S2 Questionnaire Questionnaire to women in Bicol. (PDF)

S3 Questionnaire Questionnaire to women in Chavacano. (PDF)

S4 Questionnaire Questionnaire to women in Ilocano. (PDF)

S5 Questionnaire Questionnaire to women in Ilonggo. (PDF)

S6 Questionnaire Questionnaire to women in Meranao. (PDF)

S7 Questionnaire Questionnaire to women in Tagalog. (PDF)

S8 Questionnaire Questionnaire to women in Tausug. (PDF)

S9 Questionnaire Questionnaire to women in Visaya. (PDF)

S10 Questionnaire Facility assessment checklist. (PDF)

S1 Minimal Dataset 1:Response of 860 women in Excel. (XLSX)

S2 Minimal Dataset 2:Response of 860 women in STATA. (DTA)

S3 Minimal Dataset 3:Values used to build graphs. (XLSX)
