# Supplementary material for: Opportunities lost: Barriers to increasing the use of effective contraception in the Philippines
Source: PLoS One. 2019 Jul 25;14(7):e0218187. doi: 10.1371/journal.pone.0218187 (PMC6657820; doi:10.1371/journal.pone.0218187)
Supplement: S1 Questionnaire — (PDF) [file pone.0218187.s001.pdf]

RESPONDENT NUMBER:

Region	Province	City/Mun	Brgy	Fac. No	Respondent No.

As of July, 19 2017

COVER PAGE

FORM1. Interview Women of Reproductive Age Who Are Not Currently Pregnant or within 6 Weeks of Delivery, and Desire Delaying or Limiting Childbearing.

[Fill one number for each woman contacted in the order they were contacted at the health facility; if done over several days, continue unique sequence numbers]

Identification of interview place		
Region		
Province		
City/Municipality		
Barangay		
Health facility name		
Home address **(for home visit only)**		
Latitude and longitude (Use the coordinate of GPS in a mobile phone)	Latitude	Longitude
Interview Record		
Date of interview **(dd/mm/yyyy)**		
Interviewer's name **(Last Name, First Name, Middle Initial)**		
Health facility level where interview took place	**Public Sector**<br>1. Government hospital<br>2. Rural health unit/Urban health center/Barangay health center<br>3. Barangay health station<br>4. Barangay supply/Service point office/BHW<br>5. Other public (specify) ___________<br><br>**Other places**<br>6. Puericulture center<br>7. Others (specify) _____________	
**FOR LARGE HOSPITALS**<br>**Reason for visiting the health facility**	1. Reproductive health clinic for postnatal care<br>2. Reproductive health clinic not related to postnatal care<br>3. Receiving vaccination or routine check-up for **child <5 years old**<br>4. Seeking medical advice or treatment for sickness or injury of child **<5 years old**<br>5. Seeking medical advice or treatment for sickness or injury of herself<br>6. Others (specify)_________	

RESPONDENT NUMBER:

Region	Province	City/ Mun	Brgy	Facility no.	Respondent No.

SCREENING FORM

[**CONSIDER ALL THE WOMEN AT THE HEALTH FACILITY AS OUR TARGET. THEN WE SCREEN THOSE WOMEN THROUGH Q001-010.]**

State: "We would like to start by asking a few questions that determine if you are eligible for the survey."

			Answer	
001	How old were you on your last birthday?	Age in completed years		18-49 years ->002<br>Others -> 010
002	Are you pregnant now?<br>(Stop the interview)	**1.** Yes<br>**2.** No<br>3. Unsure		1 ->010<br>2 ->003<br>3 ->003
003	What is the name of your last baby?<br>Record name	1. Name: ____________<br>2. No previous baby		1 ->004<br>2 ->006
004	In what month and year was NAME born?<br>If less than 6 weeks. Stop the interview.	Month : __ __<br>Year : __ __ __ __		Age >6 weeks ->005<br>Age<6 weeks ->010
005	Has your menstrual period returned since the birth of ___________ (NAME)?	1. Yes<br>**2.** No<br>3.		1 ->006<br>2 ->006
006	Now, I have some questions about the future. Would you like to have (a/another) child, or would you prefer not to have any (more) children?	1. Have (a/another) child<br>**2.** No more/none<br>3. Cannot get pregnant (due to tubal ligation)<br>***4.*** Cannot get pregnant<br>5. Undecided / don't know		1 -> 007<br>2 -> 008<br>3 -> 101 (count this woman as a FP user)<br>4 -> 010<br>5 -> 010
007	Do you want (a/another) child soon?	**1.** Yes<br>**2.** No, want to wait<br>3. Don't know		1 ->010<br>2 ->008<br>3- >010
008	Are you or your husband/partner currently doing something or using any method to delay or avoid getting pregnant?	**1.** Yes<br>2. No		1 -> 009<br>2 -> 101 (Count this woman as a non-FP user)
009	Which method are you or your husband using?	1. Pill, Injectable, Patch, IUD, Implants, or Sterilization<br>2. Others		1 -> 101 (Count this woman as a FP user)<br>2 -> 101 (Count this woman as a non-FP user)
010	Thank the woman, indicate ineligibility for the survey and stop the interview. **[STAPLE THE SCREENING PAGE OF THE ELIGIBLE MOTHER IN HER QUESTIONNAIRE. PLACE THE SCREENING FORM OF THE INELIGIBLE WOMAN IN THE ENVELOPE MARKED "*INELIGIBLE RESPONDENTS*"**. Enter this woman into the list of "number of women contacted". Then find another woman to interview and use another screening sheet.			

RESPONDENT NUMBER:

Region	Province	City/ Mun	Brgy	Facility no.	Respondent No.

QUESTIONNAIRE

FORM1. Interview of women of reproductive age who are not currently pregnant or within 6 weeks of delivery, and desire delaying or limiting childbearing.

[READ THE INFORMED CONSENT FORM AND REQUEST THE MOTHER TO SIGN THE FORM]

**NO.**	**Section 1. Respondent background**			
101	In (month of interview) 2017, did you live in a city, in a town proper/ *Poblacion*, in an urban barangay or rural barangay, or abroad?	1. City (City)<br>2. Town Proper/*Poblacion*<br>3. Urban Barangay<br>4. Rural barangay<br>5. Abroad (*Abroad*)<br>6. Don't Know		->102
102	What is your marital status now?	1. Never married or never lived with a man<br>2. Currently married<br>3. Currently living with a man<br>4. Divorced/separated/widow and not currently living with a man		->103
103	What is your highest level of education attended, whether or not that level was completed?	1. No education<br>2. Elementary<br>3. High school<br>4. Vocational<br>5. College<br>6. Post-graduate		->104
104	How many children do you have who are still alive?	Number of children alive		->105
105	Women sometimes have pregnancies that do not result in a live born child. That is, a pregnancy can end early, in a miscarriage or the child can be born dead. Have you ever had a pregnancy that did not end in a live birth?	1. Yes<br>2. No		1 ->106<br>2 -> 107
106	In all, how many pregnancies have you had that did not end in a live born child?<br>**[WRITE DOWN NUMBER OF SPONTANEOUS ABORTION AND INDUCED ABORTION SEPARATELY]**	A. Number of pregnancy losses by spontaneous abortion (*nalaglag).*		->107
		B. Number of pregnancy losses by induced abortion (*inilaglag*)		
107	Are you covered by any health insurance, either as member or dependent?<br>**[WRITE DOWN ALL MENTIONED.]**	1. Not covered<br>2. Philhealth<br>3. Government Service Insurance System (GSIS)<br>4. Social Security System (SSS)<br>5. Private insurance company/Health (maintenance organization /Pre-need insurance plan company<br>6. LGU card with monthly fee<br>7. LGU card for free to the residents		->201

RESPONDENT NUMBER:

Region	Province	City/Mun	Brgy	Fac. No	Respondent No.

NO.	Section 2. Current use of FP						
201	REVIEW: Are you or your husband/partner currently doing something or using any method to delay or avoid getting pregnant?	1. Yes<br>2. No					1 ->202<br>2 ->207
202	Which method are you currently using? **[WRITE DOWN ALL MENTIONED SEPARATED BY COMMA.]**						->203
	LINE NUMBER	01	02	03	04	05	
203	Now I would like to ask you one by one about all methods you are **using now**.<br>[**RECORD ALL METHODS BEING USED NOW, ONE METHOD PER ONE COLUMN NUMBER.<br>IF THERE ARE MORE THAN 5 METHODS, USE ADDITIONAL QUESTIONNAIRE.]<br>*(show card)***						->204
204	What was your source of information about the method when you first started using it?						->205
205	Where did you obtain that method when you first started using it?						1-13->206<br>14-99>207
206	What was the purpose of your going to the health facility on the day you first received the contraceptive method**?**						Repeat until all methods were explained. Then ->207

Code: 202, 203

1. Female sterilization
2. Male sterilization
3. IUD
4. Injectable (e.g., DMPA)
5. Implants
6. Patch
7. Pill
8. Male Condom
9. Female condom
10. Diaphragm
11. Foam/Jelly/Cream
12. Mucus/Billings/Ovulation
13. Basal body temperature
14. Symptothermal
15. Standard days method
16. LAM
17. Calendar/Rhythm/ Periodic abstinence
18. Withdrawal
19. Herbal
20. Other modern method (specify) _____________

Code: 204, 205

Public Sector

1. Government hospital
2. Rural health unit/Urban health center/ Barangay health center
3. Barangay health station
4. Barangay supply/Service point office/BHW
5. Other public (specify) ________

Private medical sector

6. Private hospital/clinic
7. Pharmacy
8. Private doctor
9. Private nurse, midwife
10. NGO
11. Industry-based clinic
12. Other private (specify) _________

Other sources

13. Puericulture center
14. Store
15. Church
16. Friends/Relatives
17. Internet
18. Advertisement/T.V
19. School
20. Quack doctor
21. Own self
22. Husband/Partner
23. Seminar in company

Code 206

1. Prenatal care
2. Giving birth, while a woman is still in the facility
3. Reproductive health outpatient clinic for postnatal care
4. Reproductive health clinic not related to postnatal care
5. Receiving vaccination or routine check- up for child
6. Seeking medical advice or treatment for sickness or injury of child
7. Seeking medical advice or treatment for sickness or injury of herself
8. Adolescent clinic
9. Get contraceptive
10. Seminar

99. Not Applicable

RESPONDENT NUMBER:

Region	Province	City/ Mun	Brgy	Facility no.	Respondent No.

No.	Question	Response		Skip
207	If you **<u>are not</u>** using any method to delay or avoid getting pregnant now, have you or your sexual partner done something or used a method to delay or avoid getting pregnant in the past? If **<u>you are</u>** using a method to delay or avoid getting pregnant now, have you or your sexual partner ever used a different method to delay or avoid getting pregnant in the past?	1. Yes<br>2. No		1->208<br>2-> 301
208	Which methods have you used in the past? (**[WRITE DOWN ALL MENTIONED.]**			-> 209

	LINE NUMBER	01	02	03	04	05	
209	Now I would like to ask you one by one about all methods you have used in the past.<br>**[RECORD ALL METHODS, ONE METHOD PER ONE COLUMN NUMBER AT THE RIGHT.<br>IF THERE ARE MORE THAN 5 METHODS, USE ADDITIONAL QUESTIONNAIRE.]**						-> 210
210	What was the source of information of the method when you first started using it?						-> 211
211	Where did you obtain the family planning method when you first started using it?						1-13->212<br>14-99->213
212	Why did you visit the health facility where you first started using the family planning method?						-> 213
213	Why did you stop using the family planning method that you used in the past?						-> 301

Code: 208, 209

1. Female sterilization
2. Male sterilization
3. IUD
4. Injectable (e.g., DMPA)
5. Implants
6. Patch
7. Pill
8. Male Condom
9. Female condom
10. Diaphragm
11. Foam/Jelly/Cream
12. Mucus/Billings/Ovulation
13. Basal body temperature
14. Symptothermal
15. Standard days method
16. LAM
17. Calendar/Rhythm/Periodic abstinence
18. Withdrawal
19. Herbal
20. Other modern method (specify) _______________

Code: 210, 211

Public Sector

1. Government hospital
2. Rural health unit/Urban health center/ Barangay health center
3. Barangay health station
4. Barangay supply/Service point office/BHW
5. Other public (specify) _________

Private medical sector

6. Private hospital/clinic
7. Pharmacy
8. Private doctor
9. Private nurse, midwife
10. NGO
11. Industry-based clinic
12. Other private (specify) __________

Other sources

13. Puericulture center
14. Store
15. Church
16. Friends/Relatives
17. Internet
18 Advertisement/T.V
19 School
20 Quack Doctor
21 Self/Own
22 Husband/Partner
23 Seminar in Company

99-Not Applicable **(APPLIES TO Q. 211 ONLY)**

Code 212

1. Prenatal care
2. Giving birth, while a woman is still in the facility
3. Reproductive health outpatient clinic for postnatal care
4. Reproductive health clinic not related to postnatal care
5. Receiving vaccination or routine check- up for child
6. Seeking medical advice or treatment for sickness or injury of child
7. Seeking medical advice or treatment for sickness or injury of herself
8. Adolescent clinic
9. Get contraceptive
10. Seminar
99. Not Applicable

Code: 213

1. Side effects
2. Method not available at the facility
3. Concerns about risks of pregnancy
4. Could not afford to purchase
8. Husband/partner did not support or allow to use
9. Wanted to get pregnant
10. Got pregnant
11. Switched to other method
12. Partner working away from home
16. Afraid to get pregnant
17. Expired LAM Period
18. Already old to get pregnant
19. Removed accidentally
20. Just decided

RESPONDENT NUMBER:

Region	Province	City/ Mun	Brgy	Facility no.	Respondent No.

5. Health worker did not continue to provide the method.
6. Quality of care provided by the health facility or provider
7. Advice of friends, relatives, neighbors

13. Did not like the method
14. Forgot to take Regularly
15. Advice of Health Staff

21. Concerns for side effects

	**Section 3. FP Concerns and Today's FP counseling**							
301	Do you have any concerns about any type of family planning method?	1. Yes<br>2. No						1 ->302<br>2 ->305
	LINE NUMBER	01	02	03	04	05	06	
302	About which family planning methods do you have concerns***?***<br><br>**[WRITE ONE METHOD PER CELL. IF THERE ARE MORE THAN 6 METHODS WITH CONCERN, USE ANOTHER questionnaire**<br>1. Female sterilization<br>2. Male sterilization<br>3. IUD<br>4. Injectable<br>5. Implants<br>6. Patch<br>7. Pill<br>8. Condom<br>9. Calendar/Rhythm/Period<br>10. Withdrawal							-> 303
303	What are your concerns about that family planning method?<br>[**USE ONE LINE NUMBER FOR ONE METHOD. WRITE DOWN ALL MENTIONED CONCERNS PER EACH METHOD.]**							-> 304

1. Cause cancer in the uterus
2. Cause cysts in the uterus
3. Cause infection of the uterus
4. Cause frequent bleeding
5. Cause thyroid problems
6. Cause/worsen asthma
7. Cause/worsen lots of veins
8. Cause dry skin, skin disease
9. Cause weight
10. Cause weight loss
11. Cause bloated stomach
12. Cause headache
13. Cause irritability
14. Increase libido/turn into a maniac

IUD/Implants
22. Melt or move around inside the body and doctors will not be able to find.
23. Washed away/pushed out of body.
24. Painful to insert

IUD
25. Itchy on the vagina.
26. Entangled around the man's penis
27. Messy when inserted

Male Sterilization
28. Part of the man's testicles are cut off
29. It hurts the testicles

39. Cause of Death
40. Dizziness/vomiting
41. Easily forget to take
42. Cause hearing problem
43. Cause memory loss
44. Side effects
45. Cause liver disease
46. Cause kidney disease
47. Injection is painful
48. Painful during intercourse
49. Afraid of operation (tubal ligation
50. Cannot carry heavy things
51. Pills remain in the body
52. Cannot do normal things

RESPONDENT NUMBER:

Region	Province	City/ Mun	Brgy	Facility no.	Respondent No.

15. Cause loss/reduce of libido
16. Cause loss/reduce of sexual satisfaction
17. One will not have children anymore
18. Not fully effective, woman could still get pregnant
19. When it does not work, the baby is born with abnormalities
20. Results in mortal sin because it is against church teachings
21. It is not legally allowed to use
30. The man loses his manhood (*pagkalalaki*)
31. Delayed menstruation
32. Cause Heart Problem
33. Painful during menstruation
34. Do not like pap smear while taking pills
35. Cause hypertension
36. Cause hypotension
37. Cause Myoma
38. Delayed Menstruation due to Myoma
53. Quality control of the commodity
54. Cause cramps
55. Cause Edema
56. Cause pain in the hips
57. IUD doesn't fit
58. Cause for losing consciousness
59. Cause bad effect in breastfeeding
60. Cause of poor eyesight
61. Diarrhea

	LINE NUMBER	01	02	03	04	05	06	
304	Who told you or how did you find about your concerns about family planning methods?<br>**[REPEAT EACH CONCERN IN TURN. FOR EACH WRITE DOWN ALL SOURCES OF INFORMATION.]**<br>1. Health staff<br>2. BHW or health volunteers<br>3. Husband or partner did not want to use<br>4. Friend, neighbours, relatives<br>5. Church<br>6. Radio<br>7. Television<br>8. Newspaper or magazine<br>9. Online or internet<br>10. Own experience<br>11. Seminar							-> 305

[IF THE WOMAN HAS NOT HAD THE CONSULTATION GO TO SECTION 4 AND ASK QUESTIONS 305-321 AFTER THE CONSULTATION.]

RESPONDENT NUMBER:

Region	Province	City/Mun	Brgy	Fac. No	Respondent No.

305	Today, did any staff member at the health facility speak to you about family planning methods?	1. Yes<br>2. No		1 ->306<br>2 ->317
306	Did the health staff ask you about your concerns on family planning**?**	1. Yes<br>2. No		1 ->307<br>2 ->317
307	Do you feel the health staff understands your concerns?	1. Yes<br>2. No		-> 308
308	Did the health staff help you to find solutions to your concerns?	1. Yes<br>2. No		-> 309
309	Did the health staff offer you information how **different family planning methods** work?	1. Yes<br>2. No		1 ->310<br>2 ->312
310	Which methods did the health staff mention today? **[WRITE DOWN ALL MENTIONED SEPARATED BY COMMA.]**	1. Female sterilization<br>2. Male sterilization<br>3. IUD<br>4. Injectable (e.g., DMPA)<br>5. Implants<br>6. Patch<br>7. Pill<br>8. Male Condom<br>9. Female condom<br>10. Diaphragm<br>11. Foam/Jelly/Cream<br>12. Mucus/Billings/Ovulation<br>13. Basal body temperature<br>14. Symptothermal<br>15. Standard days method<br>16. LAM<br>17. Calendar/Rhythm/Periodic abstinence<br>18. Withdrawal<br>19. Herbal<br>20. Other modern method (specify) ______		-> 311
311	Did the health staff tell you about side-effects or problems you might have with any methods of family planning?	1.Yes<br>2.No		-> 312
312	Did the health worker offer you information how **your family planning method** (which you are now using) works?	1. Yes<br>2. No<br>99. N/A (not using a method now)		1 -> 313<br>2-> 313<br>3-> 315
313	Did the health staff explain about the side effects of your current method?	1. Yes<br>2. No		-> 314
314	Did the health staff ask you to describe how you use your current method?	1. Yes<br>2. No		-> 317
315	After receiving FP counselling will you begin using a family planning method today?	1. Yes<br>2. No		1 -> 317<br>2 -> 316
316	After receiving FP counselling, do you think you will use a contraceptive method anytime in the future?	1. Yes<br>2. No		1 -> 317<br>2 -> 321

317	Which contraceptive method would you prefer most to use? (**ONE METHOD ONLY**)	1. None<br>2. Female sterilization<br>3. Male sterilization<br>4. IUD<br>5. Injectable (e.g., DMPA)<br>6. Implants<br>7. Patch<br>8. Pill<br>9. Male Condom<br>10. Female condom<br>11. Diaphragm<br>12. Foam/Jelly/Cream<br>13. Mucus/Billings/Ovulation<br>14. Basal body temperature<br>15. Symptothermal<br>16. Standard days method<br>17. LAM<br>18. Calendar/Rhythm/Periodic abstinence<br>19. Withdrawal<br>20. Herbal<br>21. Other modern method (specify) ___		1-> 321<br>2-21-> 318
318	Why do you prefer the method?	1. Free<br>2. Always available<br>3. No side effects<br>4. Easy to use<br>5. Recommended by health staff<br>6. Recommended by friends, neighbours, relatives<br>7. Lifetime effectivity<br>8. 3 months effectivity<br>9. 3 years effectivity<br>10. 10 years effectivity<br>11. 100% effective for this person<br>12. Affordable price<br>13. Improves communication/understanding with husband<br>14. 6 months (LAM)<br>15. No side effects for her<br>16. Happy to gain weight<br>17. stops menstruation (less hassle)<br>18. Regularity of menstruation		1-2-> 401<br>3-7->319
319	Will you use the method?	1. Yes<br>2. No		1->401<br>2->320
320	Why do you think you will not use your preferred method?	1. Perceived to be expensive<br>2. No idea where and how to avail<br>3. Advised by the doctor not to use due to_______<br>4. Ligated<br>5. Side effects<br>6. Gain Weight<br>7. Husband disagrees<br>8. No menstruation yet<br>9. Sexually inactive<br>10. Painful<br>11. Undecided		-> 401

Region	Province	City/ Mun	Brgy	Facility no.	Respondent No.

321	Why do you think you will not use any contraceptive method anytime in the future	1. Afraid of side effects<br>2. Already old to get pregnant<br>3. Not supported by husband<br>4. No need<br>5. hard to get pregnant<br>6. Not enough information<br>7. Against religion<br>8. Want to get pregnant<br>9. No reason<br>10. Hard to use<br>11. Not legally use<br>12. Not yet thinking about it		-> 401

	**Section 4. Past Health Facility visit and FP Counseling**			
401	From January 1- December 31, 2016, have you visited a health facility for care for yourself or your children for any purpose?	1. Yes<br>2. No		1 -> 402<br>**2 -> END OF INTERVIEW**

RESPONDENT NUMBER:

Region	Province	City/Mun	Brgy	Fac. No	Respondent No.

	LINE NUMBER	01	02	03	04	05	06	
402	Now I would like to record all your facility visits from January 1 – December 31, 2016. Start with the latest visit you had. Why did you visit a health facility? **[AFTER WRITING THE FIRST VISIT IN LINE NUMBER 01, ASK Q403-410 FOR THAT VISIT. THEN ASK THE 2nd LATEST VISIT TO WRITE IN 402 LINE NUMBER 02, THEN ASK Q 403 AND Q404. REPEAT FOR ALL HEALTH FACILITY VISITS FROM JANUARY 1 - DECEMBER 31, 2016.] [IF THERE ARE MORE THAN 6, USE AN ADDITIONAL QUESTIONNAIRE.]**							-> 403
403	Where did you visit?							-> 404
404	At that visit, were you or your sexual partner already using any method to delay or avoid getting pregnant? 1=Yes 2=No							1 -> 405<br>2 -> 406
405	Which method(s) were you using? **[WRITE DOWN ALL MENTIONED.]**							->406

Code 402

1. Prenatal care
2. Giving birth, while a woman is still in the facility
3. Reproductive health outpatient clinic for postnatal care
4. Reproductive health clinic not related to postnatal care
5. Receiving vaccination or routine check-up for child
6. Seeking medical advice or treatment for sickness or injury of child
7. Seeking medical advice or treatment for sickness or injury of herself
8. Adolescent clinic
9. Get contraceptive
10. Seeking medical advice or treatment for sickness of husband

99. Not Applicable

Code: 403

Public Sector

1. Government hospital
2. Rural health unit/Urban health center/ Barangay health center
3. Barangay health station
4. Barangay supply/Service point office/BHW
5. Other public (specify) ________

Private medical sector

6. Private hospital/clinic
7. Pharmacy
8. Private doctor
9. Private nurse, midwife
10. NGO
11. Industry-based clinic
12. Other private (specify) __________

Other sources

13. Puericulture center
14. Store
15. Church
16. Friends/Relatives
17. Internet
18 Advertisement/T.V
19 School
20 Quack Doctor
21 Self/Own
22 Husband/Partner
23 Seminar in Company

99-Not Applicable

Code: 405

1. Female sterilization
2. Male sterilization
3. IUD
4. Injectable (e.g., DMPA)
5. Implants
6. Patch
7. Pill
8. Male Condom
9. Female condom
10. Diaphragm
11. Foam/Jelly/Cream
12. Mucus/Billings/Ovulation
13. Basal body temperature
14. Symptothermal
15. Standard days method
16. LAM
17. Calendar/Rhythm/Periodic abstinence
18. Withdrawal
19. Herbal
20. Other modern method (specify) ______________

RESPONDENT NUMBER:

Region	Province	City/ Mun	Brgy	Facility no.	Respondent No.

	LINE NUMBER	01	02	03	04	05	06	
406	At that visit, did any staff member at the health facility speak to you about family planning methods? 1-Yes 2-No							1-> 407<br>2-> 402 next column number
407	After that visit, did you start using any FP method or change from your previous method to a new method? 1=Yes 2=No							1 -> 409<br>2 -> 408
408	If you did not start a new method or change from your previous method, why?<br>1. No need<br>2. Possible side effects of new method<br>3. New method not available at the facility<br>4. Concerns about risk of pregnancy with new method<br>5. Not enough information<br>6. Could not afford to purchase<br>7. Advice of friends, relatives, neighbors not to start or change<br>8. Husband/partner did not support<br>9. No menstruation yet/Lactational Amenorrhea (LA)<br>10. Pregnant at that time<br>11. Advice by BHW not to use due to old age<br>12. Wants another baby<br>13. I am too old<br>14. Religion							
409	Which FP method did you start using after that visit or which new method did you change to?							-> 402 next column number
	1. Female sterilization<br>2. Male sterilization<br>3. IUD<br>4. Injectable (e.g., DMPA)<br>5. Implants<br>6. Patch<br>7. Pill<br>8. Male Condom<br>9. Female condom<br>10. Diaphragm<br>11. Foam/Jelly/Cream<br>12. Mucus/Billings/Ovulation<br>13. Basal body temperature<br>14. Symptothermal<br>15. Standard days method<br>16. LAM<br>17. Calendar/Rhythm/Periodic abstinence<br>18. Withdrawal<br>19. Herbal<br>20. Other modern method (specify) ____________							

END OF THE INTERVIEW
