# Supplementary material for: Opportunities lost: Barriers to increasing the use of effective contraception in the Philippines
Source: PLoS One. 2019 Jul 25;14(7):e0218187. doi: 10.1371/journal.pone.0218187 (PMC6657820; doi:10.1371/journal.pone.0218187)
Supplement: S2 Questionnaire — (PDF) [file pone.0218187.s002.pdf]

COVER PAGE

FORM1. Iinterviewwomen of reproductive age who are not currently pregnant or within 6 weeks of delivery, and desire delaying or limiting childbearing

Sequence number: _____________

[Fill one number for each woman contacted in the order they were contacted at the health facility; if done over several days, continue unique sequence numbers]

<table>
<tr><td colspan="3">Identification of interview place</td></tr>
<tr><td>Region</td><td colspan="2"></td></tr>
<tr><td>Province</td><td colspan="2"></td></tr>
<tr><td>CITY/MUNICIPALITY</td><td colspan="2"></td></tr>
<tr><td>BARANGAY</td><td colspan="2"></td></tr>
<tr><td>Health facility name</td><td colspan="2"></td></tr>
<tr><td>Home address (for home visit only)</td><td colspan="2"></td></tr>
<tr><td>Latitude and longitude
(Use the coordinate of GPS in a mobile phone)</td><td colspan="2"></td></tr>
<tr><td colspan="3">Interview Record</td></tr>
<tr><td>Date of interview</td><td colspan="2"></td></tr>
<tr><td>Interviewer’s name</td><td colspan="2"></td></tr>
<tr><td>Health facility level where interview took place</td><td>1. National hospital
2. Regional hospital/Public medical center
3. Provincial hospital
4. District hospital
5. Municipal hospital
6. Rural health unit (RHU)/urban health center(UHC)/Lying-in
7. Barangay health station (BHS)
8. Barangay supply/service point officer/BHW
9. Mobile clinic
10.Other (specify)</td><td></td></tr>
<tr><td>Clinic where interview took place (for large hospitals)</td><td>1. Postnatal health check after giving birth, after a woman left the facility
2. Receiving vaccination or routine check-up for child
3. Seeking medical advice or treatment for sickness or injury of child
4. Seeking medical advice or treatment for sickness or injury of herself
5. Adolescent clinic
6. Other (specify)</td><td></td></tr>
</table>

Sequence Number: _________________
[Write the same sequence number from Cover Page]

Instructions:
Read the information sheet. Answer questions. If the woman agrees to participate give the certificate of consent for her to sign. Then start the Screening Form.

Screening Form

State: "We would like to start by asking a few questions that determine if you are eligible for the survey." (Uumpisahankonapoangpagtatanong ng ilangmgakatanungan para alamin kung kayo po ay nararapatnainterbyuhin para sa survey naito.)
(Puunan ko napo ang mga hapot para po maaraman kung pwede po kamo interbyuhon para sa survey na ini)

00<br>1	How old were you on your last birthday? (Ilangtaonnapo kayo noonghuling birthday?)<br>-Pirang taon ka na po kan huring birthday mo?	Age in completed years<br>(Edad)		15-49 years ->002<br>Other -> 009
00<br>2	Are you pregnant now? (Nagdadalantao/Buntispo kayo ngayon?)<br>-Bados ka po ngonyan?	1. Yes (Oo) -Iyo<br>2. No (Hindi) -Dai<br>3. Unsure (Hindi SIgurado) -Dai sigurado		1 ->009<br>2 ->003<br>3 ->003
00<br>3	What is the name of your last baby? (Anopopangalang ng inyongpinakabatanganak?)<br>Record name<br>-Ano po an pangaran kan bunso mong aki?	1. Name: _____________<br>2. No previous baby		1 ->004<br>2 ->006
00<br>4	In what month and year was NAME born? (Anopoangbuwan at taonipinanganaksi __________)<br>-Anong bulan saka taon po pinangaki si _____<br>(probe: when is his or her birthday) (PROBE: Kailanpoang birthday ni _____________)<br>-Nuarin po an birthday ni _____?	Month (Buwan) - Bulan: __ __<br><br>Year (Taon): __ __ __ __		Age ≥6wks ->005<br>Age<6wks ->009
00<br>5	Has your menstrual period returned since the birth of NAME? (Bumaliknapoba ng inyongreglapagkatapos manganak kay _________?)<br>-Nagbalik na po an saimong regal pagka pangaki mo ki _____?	1. Yes -Iyo<br>2. No -Dai		1 ->006<br>2 ->006
00<br>6	Now I have some questions about the future. Would you like to have (a/another) child, or would you prefer not to have any (more) children? (Ngayonnamanpo, may ilanpoakongkatanungantungkolsamgadaratingnapanahon. Gusto pobaninyo pang magkaroonuling anak o masgugustuhinninyona di namagkaanak?	1. Have (a/another) child (Magkaroonulit ng anak) Magka igwa ulit nin aki<br>2. No more/none (Hindi na) Dai na		1 -> 007<br>2 ->008<br>3 ->009<br>4 ->009

	-Ngonyan po, may mga kahaputan po ako manungod sa maabot na panahon. Gusto pa po nindo na magka igwa nin aki o mas gusto po nindo na dai na magka aki?	3. Cannot get pregnant (Hindi napwedemanganak) Dai na pwede magka aki<br>4. Undecided / don't know (Hindi koalam) Dai ko aram		
007	Do you want (a/another) child soon? (Gusto pobaninyonamagkaanakagad?)<br>Gusto po nindo na magka aki na tulos?	1. Yes (Oo) Iyo<br>2. No, want to wait (Hindi pa, gusto ko pang maghintay) Dai pa, gusto ko pa maghalat<br>3. Don't know (Hindi koalam) Dai ko aram		1 ->009<br>2 ->008<br>3- >009
008	Are you or your husband/partner currently doing something or using any method to delay or avoid getting pregnant? (Kayo poba o anginyongasawa/partner ay may ginagamitnaparaan para madelay o maiwasanangmagbuntis?)<br>- Kamo po kan saimong agom/partner may ginagamit na paagi para maiwasan na mag bados ka po?	1. Yes (Oo) Iyo<br>2. No (Hindi) Dai		1 ->101<br>2 ->101<br>To achieve a total of 5 users and non-users (hospitals) and 3 users and 3 non-users (health centres).
009	Thank the woman, indicate ineligibility for the survey and stop the interview. Enter this woman into "number of women contacted". Then find another woman to interview. (Pasalamatanangkausap, sabihannataposnaang interview. Ilagayangpangalansalistahan ng mgababaenakinausap". Maghanapulit ng susunodnaiinterviewhin.<br>Pasalamatan an nakaulay, sabihan na tapos na an interbyu. Ilaag ang pangaran sa listahan kan mga babae na nakaulay. Maghanap na ulit nin sunod na iinterbyuhon.			

QUESTIONNAIRE

FORM1. Interviewof women of reproductive age who are not currently pregnant or within 6 weeks of delivery, and desire delaying or limiting childbearing.*(Interbyu ng mgababaenakasalukuyan ay hindibuntis o nakapanganaknanang lampas saanimnabuwan, at nagnanaisnaidelay o iniiwasanangmalimitna [agbubuntis.)*

Interbyu nin mga babae na dai bados o nakapangaki pa sana nin lampas sa anom na bulan, saka may gustong i-delay o iniiwasan muna an magbados

Sequence Number:
[Write the same sequence number from Cover Page]

NO.					
101	In (month of interview) 2016, did you live in a city, in a town proper/ poblacion, in the barrio or rural area, or abroad? (Ngayon pong buwan ng ________________, kayo poba ay tumirasa city o bayan, sa barangay o saibangbansa?) Ngonyan po na bulan nin _______, nag istar po kamo sa ciudad, bayan, barangay o sa ibang bansa?				1. City (City) *(Syudad)*<br>2. TOWN PROPERIPOBLACION (*Bayan/Poblacion*)<br>3. BARRIO/RURAL AREA (Barangay)<br>4. ABROAD (*Abroad*)<br>5. DON'T KNOW (Hindi alam.) Dai aram
102	What is your marital status now? (Kayo poba ay __________________ sakasalukuyan Ano po an estado mo sa ngonyan? May agom ka po o mayo?				1. Never married or never lived with a man (*walangasawa o hindinagkaroon ng kinakasama) Mayong agom o dai nagka igwa nin kaiba*
					2. Currently married (*Kasalukuyang may asawa) May agom*
					3. Currently living with a man (kasalukuyang may kinakasamanglalaki) Kasalukuyang may kasaro (ka live-in)
					3. Divorced/separated/widow and not currently living with a man (diborsyado/hiwalay/balo at kasalukuyangwalangkinakasamanglalaki) Diborsyado/Suway sa agom/Balo/Sa Presente mayong kasaro
103	What is your highest level of education attended, whether or not that level was completed?*(Anopoanginyongnataposnaantas ng pagaaral?)* *Ano po an saimong tinapos sa pagklase?*				1. No education (Wala) Mayo<br>2. Elementary (Elementarya)<br>3. High school (High School)<br>4. College (Kolehiyo)<br>5. Post-graduate (Masteral o Ph D)
104	How many children do you have who are still alive? (Ilanpoanginyongbuhaynaanak?) Pira po an mga buhay mong aki?				Number of children alive (Bilang ng anaknabuhay)
105	Did you or someone else do anything to end any of your past pregnancies? (Kayo poba or may ibangtao ay may ginawa para sapilitangtapusinanginyong [pagbubuntis?) May ginibo ka po o an ibang tao para mawara an saimong dinadara kaidto?				1. Yes (Oo) Iyo<br>2. No (Hindi) Dai
106	How many pregnancies did you or someone else do anything to end? *Pira po sa saimong mga*				Number of induced abortion *(Bilang ng sapilitangtinaposnapagbubuntis.)*

	pinagbados an sapiritang tinanggal mo po o kan ibang tao?	*Bilang kan sapiritang pagtapos/pagtanggal kan binabados*
107	Are you covered by any health insurance, either as member or dependent? (*Meronpobakayong health insurance bilangmiyembro o dependent.)?* *Igwa po kamo nin health insurance bilang miyembro o dependent?*	1. Not covered (*Walang insurance*) Mayong insurance<br>2. Philhealth<br>3. Government Service Insurance System (GSIS)<br>4. Social Security System (SSS)<br>5. Private insurance company/Health (maintenance organization /Pre-need insurance plan company (*Pribadong health insurance.)*<br>6. Other (Specify)(*Iba pa… pakisabi..)*
201	REVIEW: Are you or your husband/partner currently doing something or using any method to delay or avoid getting pregnant? (Kayo poba o anginyongasawa/kapartner ay may ginagawa o may ginanagamitnapamamaraan para ipagpaliban o maiwasanna kayo ay magbuntis?) Kamo po kan saimong agom/partner may ginigibo o may ginagamit po kamong paagi para ma-delay o maiwasan na dai magbados?	4. Yes (Iyo)<br>5. No (Dai)
202	Which method are you currently using? (Ano pong pamamaraan o method anginyongginagamit?) Ano po an mga paagi na saindong ginagamit?<br><br>WRITE DOWN ALL MENTIONED.	1. Female sterilization<br>2. Male sterilization<br>3. IUD<br>4. Injectable (e.g.DMPA)<br>5. Implants<br>6. Patch<br>7. Pill<br>8. Condom<br>9. Female condom<br>10. Diaphragm<br>11. Form/Jelly/Cream<br>12. Mucus/Billings/Ovulation<br>13. Basal body temperature<br>14. Symptothermal<br>15. Standard days method<br>16. LAM<br>17. Calendar/Rhythm/Periodic abstinence<br>18. Withdrawal<br>19. Other traditional method<br>20. Other modern method (specify)

	LINE NUMBER	01	02	03	04	05
203	Now I would like to ask you one by one about all methods you are using now.*(Ngayonnamanpo ay isa-isakongtatanungintungkolsalahat ng pamamaraannaginagamitninyongayon.)* *Ngonyan po hahaputon taka po saro-saro kan saindong mga ginagamit na paagi sa ngonyan.*					

	RECORD ALL METHODS BEING USED NOW, ONE METHOD PER ONE LINE NUMBER.<br>IF THERE ARE MORE THAN 5 METHODS, USE ADDITIONAL QUESTIONNAIRE.*(ISULAT LAHAT NG PAMAMARAAN NA GINAGAMIT SA KASALUKUYAN, ISANG PAMAMARAAN SA BAWAT LINYA. KUNG MAY HIGIT SA LIMANG PAMAMARAAN, GUMAMIT NG ISA PANG QUESTIONNAIRE)*<br>*Isurat gabos na paagi/method na ginagamit ngonyan, sarong paagi/method kada linya. Kung sobra sa limang paagi/method, mag gamit nin saro pang questionnaire*<br>1. Female sterilization<br>2. Male sterilization<br>3. IUD<br>4. Injectable (e.g.DMPA)<br>5. Implants<br>6. Patch<br>7. Pill<br>8. Condom<br>9. Female condom<br>10. Diaphragm<br>11. Form/Jelly/Cream<br>12. Mucus/Billings/Ovulation<br>13. Basal body temperature<br>14. Symptothermal<br>15. Standard days method<br>16. LAM<br>17. Calendar/Rhythm/Periodic abstinence<br>18. Withdrawal<br>19. Other traditional method<br>20. Other modern method (specify)					
204	Where did you obtain that method when you first started using it?*(Nang magumpisapo kayo nagumamit ng _______ san po kayo kumuhanito.)*<br>*Kan inot nindo ining method gamiton, sain po nindo ini nakua?*<br>1. National hospital<br>2. Regional hospital/Public medical center<br>3. Provincial hospital<br>4. District hospital<br>5. Municipal hospital<br>6. Rural health unit (RHU)/urban health center(UHC)/Lying-in<br>7. Barangay health station (BHS)<br>8. Barangay supply/service point officer/BHW<br>9. Mobile clinic					

	10.Other (specify. Private facility is included here)					
205	What was the purpose of your going to the health facility on the day you first received the contraceptive method?*(Anopoangdahilan at kayo ay nagpuntasa health center noongunakayongnakatanggap/gumamit ng*<br>______________<br>*Ano po an naging dahilan nindo kan pagduman sa health center kan aldaw na nakua nindo an contraceptive method?*<br>1. Prenatal care<br>2. Giving birth, while a women is still in the facility<br>3. Health check after giving birth, after a woman left the facility<br>4. Receiving vaccination or routine check up for child<br>5. Seeking medical advice or treatment for sickness or injury of **child**<br>6. Seeking medical advice or treatment for sickness or injury of **herself**<br>7. Adolescent clinic<br>8. Other (specify)					
206	If you <u>are not</u> using any method to delay or avoid getting pregnant now, have you or your sexual partner done something or used a method to delay or avoid getting pregnant in the past? *(Kung kayo po ay hindigumagamitnanganomangparaan para madelayangpagbubuntis, kayo poba o anginyongkapartner may ginagawa or ginagamitnapamamaraan para madelay or hindimagbuntisnoongunangpanahon?)*<br>Kun kamo po dai nag gagamit nin maski anong paagi para ma-delay o maiwasan an saimong pagbados ngonyan, kaidto po ba may ginamit po kamo kan saimong agom/partner?<br>If <u>you are</u> using a method to delay or avoid getting pregnant now, have you or your sexual partner ever used a different method to delay or avoid getting pregnant in the past?*(Kung kayo ay gumagamit ng method oparaan para mdelay or huwagmagbuntissangayon, kayo ba o anginyongkapartner ay gumamit ng ibangpamamaraan o method para madelay o hindimagbuntinoongunangpanahon?)*<br>Kun kamo po kan saimong agom/partner nag gagamit nin maski anong paagi para ma-delay o maiwasan an saimong pagbados ngonyan, kaidto po ba may ginamit din po kamo kan saimong agom/partner?	1. Yes (Iyo)<br>2. No (Dai)				
207	Which methods have you used in the past?*(Alin pong method opamamaraananginyongginamitnoongnakaraangpanahon?)*<br>Arin pong mga paagi an ginamiy nindo kaidto?	1. Female sterilization<br>2. Male sterilization<br>3. IUD<br>4. Injectable (e.g.DMPA)				

	WRITE DOWN ALL MENTIONED. (Isulatlahat ng pamamaraan o method.)<br>Isurat gabos na paagi o method	5. Implants<br>6. Patch<br>7. Pill<br>8. Condom<br>9. Female condom<br>10. Diaphragm<br>11. Form/Jelly/Cream<br>12. Mucus/Billings/Ovulation<br>13. Basal body temperature<br>14. Symptothermal<br>15. Standard days method<br>16. LAM<br>17. Calendar/Rhythm/Periodic abstinence<br>18. Withdrawal<br>19. Other traditional method<br>20. Other modern method (specify)					
	LINE NUMBER	01	02	03	04	05	06
208	Now I would like to ask you one by one about all methods you have used in the past. *(Ngayonnamanpo ay isa-isakongtatanungintungkolsalahat ng pamamaraannaginagamitninyonoongnakaraangpanahon).*<br>Ngonyan po, hahaputon taka po saro-saro kan mga paagi/methods na ginamit nindo kaidto?<br>RECORD ALL METHODS, ONE METHOD PER ONE LINE NUMBER. *(ISULAT LAHAT NG PAMAMARAAN NA GINAGAMIT NOONG NAKARAAN, ISANG PAMAMARAAN SA BAWAT LINYA.)*<br>Isurat gabos na paagi na ginamit kaidtong panahon, sarong paagi kada linya<br><br>IF THERE ARE MORE THAN 5 METHODS, USE ADDITIONAL QUESTIONNAIRE.*(KUNG MAY HIGIT SA LIMANG PAMAMARAAN, GUMAMIT NG ISA PANG QUESTIONNAIRE)*<br>Kun sobra sa lima ang paagi na ginamit, mag gamit nin saro pang questionnaire<br><br>1. Female sterilization<br>2. Male sterilization<br>3. IUD<br>4. Injectable (e.g.DMPA)<br>5. Implants<br>6. Patch<br>7. Pill<br>8. Condom<br>9. Female condom<br>10. Diaphragm<br>11. Form/Jelly/Cream<br>12. Mucus/Billings/Ovulation						

	13. Basal body temperature<br>14. Symptothermal<br>15. Standard days method<br>16. LAM<br>17. Calendar/Rhythm/Periodic abstinence<br>18. Withdrawal<br>19. Other traditional method<br>20. Other modern method (specify)						
209	Where did you obtain the family planning method when you first started using it?*(Nang magumpisapo kayo nagumamit ng ________ san po kayo kumuhanito.)*<br>*Sain po nindo nakua an family planning method kan inot na paggamit nindo kaini?*<br><br>1. National hospital<br>2. Regional hospital/Public medical center<br>3. Provincial hospital<br>4. District hospital<br>5. Municipal hospital<br>6. Rural health unit (RHU)/urban health center(UHC)/Lying-in<br>7. Barangay health station (BHS)<br>8. Barangay supply/service point officer/BHW<br>9. Mobile clinic<br>10.Other (specify. Private facility is included here.)						
210	Why did you visit the health facility where you first started using the family planning method?*(Anopoangdahilan at kayo ay nagpuntasa health center noongunakayongnakatanggap/gumamit ng* _________________<br>Ano po an dahilan kan pagduman nindo sa health center kan inot nindong nakua/ginamit an family planning method?<br>1. Prenatal care<br>2. Giving birth, while still in the facility<br>3. Health check after giving birth, after leaving the facility<br>4. Receiving vaccinations or routine check-ups for a child<br>5. Seeking medical advice or treatment for sickness or injury of a **child**<br>6. Seeking medical advice or treatment for sickness or injury of **herself**<br>7. Adolescent clinic<br>8. Other (specify)						
211	Why did you stop using the family planning method that you used in the past? (Bakitponinyoitinigilangpaggamit ng<br>_________________________<br>Tanu po ta nagpundo kamo maggamit kan family planning method na ginamit nindo kaidto?<br>1. Side effects						

	2. Method not available at the facility<br>3. Concerns about risks of pregnancy<br>4. Could not afford to purchase<br>5. Health worker did not continue to provide the method<br>6. Advice of friends, relatives, neighbors<br>7. Husband/partner did not support<br>8. Wanted to get pregnant<br>9. Other (specify):___________						

	**Section 3. FP Concerns and Today's FP counseling**							
	Do you have any health concerns about any type of family planning method?<br>May mga epekto/alalahanon po sa saindong salud/kalusugan an mga family planning method?	1. Yes Meron Igwa<br>2. No Wala Mayo						
	LINE NUMBER		01	02	03	04	05	06
	What are your health concerns about family planning methods? *(Ano-anopoanginyongmgaalalahaningipektoipektosakalusugan ng bawatpamamaraan?)*<br>Anu-ano po an mga epekto/alalahanon sa saindong salud/kalusugan kan kada paagi (method)?<br><br>Please tell me one by one.*(Pakisabiponinyoangbawatisa.)*<br>Pakisabi po sako saro-saro.<br><br>USE ONE LINE NUMBER FOR ONE CONCERN. WRITE DOWN ALL MENTIONED CONCERNS. (Isanglinyasabawatalalahanin. Isulatanglahatnaalalahanin.)<br>Sarong linya kada epekto/alalahanon. Isurat gabos na epekto/alalahanon<br><br>IF THERE ARE MORE THAN 6 CONCERNS, USE ADDITIONAL QUESTIONNAIRE.*KUNG MAY HIGIT SA ANIM NA PAMAMARAAN, GUMAMIT NG ISA PANG QUESTIONNAIRE) Kun sobra sa anom na paagi, mag gamit nin saro pang questionnaire*<br><br>1. Cause cancer in the uterus<br>2. Cause cysts in the uterus<br>3. Cause infection of the uterus<br>4. Cause frequent bleeding<br>5. Cause thyroidproblems<br>6. Cause/worse asthma<br>7. Cause/worse lots of veins<br>8. Cause dry skin, skin disease<br>9. Cause edema<br>10.Cause weight gain<br>11.Cause weight loss<br>12.Cause bloated stomach<br>13.Cause headache<br>14.Cause irritability<br>15.Increase libido/turn into a maniac<br>16.Cause loss/reduce of libido							

	17.Cause loss/reduce of sexual satisfaction<br>18.One will not have children anymore<br>19.Not fully effective, woman could still get pregnant<br>20.When it does not work, the baby is born with abnormalities<br>21.Results in mortal sin because it is against church teachings<br><br>**IUD/Implants**<br>22.Melt or move around inside the body and doctors will not be able to find<br>23.Washed away/pushed out of body<br>24.Painful to insert<br><br>**IUD**<br>25.Itchy on the vagina<br>26.Entangled around the man's penis<br>27.Messy when inserted<br><br>**Male sterilization**<br>28.Part of the man's testicles are cut off<br>29.It hurts the testicles<br>30.The man loses his manhood ("kapon")<br><br>31. Others (specify)						
	About which family planning methods do you have concerns? (Alin pong pagpaplano ng pamilyaangmeronkayongalalahanin o agam-agam?)<br>Arin po na paagi/method an igwa po kamo alalahanon?<br><br>REPEAT EACH CONCERN IN TURN. FOR EACH CONCERN, WRITE DOWN ALL METHODS CAUSING THAT CONCERN.*(ULITIN ANG BAWAT ALALAHANIN. SA BAWAT ALALAHANIN, ISULAT LAHAT NG PARAAN NG PAGPAPLANO NG PAMILYA.)*<br>Uliton an kada alalahanon. Sa kada alalahanon, isurat gabos na paagi/method na nagtatao nin alalahanon.<br>1. Female sterilization<br>2. Male sterilization<br>3. IUD<br>4. Injectable<br>5. Implants<br>6. Patch<br>7. Pill<br>8. Other modern method (specify)<br>9. Other method (specify)						

	Who told you or how did you find about your concerns about family planning methods?<br>*(Sino poangnagsabisainyo o paano kayo nagkaroon ng agam-agam o alalahanintungkolsapamaraan ng pagpaplano ng pamilya.)*<br>Sisay an nagsabi o panu kamo nag ka igwa nin alalahanon tungkol sa mga paagi kan family planning<br>REPEAT EACH CONCERN IN TURN. FOR EACH WRITE DOWN ALL SOUCES OF INFORMATION. (ULITIN ANG BAWAT ALALAHANIN. SA BAWAT PAMAMARAAN, ISULAT ANG BAWAT PINAGMULAN NG INPORMASYON)<br>Uliton ang kada alalahanon. Sa kada paagi, isurat an kinuanan nin impormasyon<br>1. Health staff<br>2. BHW or health volunteers<br>3. Husband or partner<br>4. Friend, neighbours, relatives<br>5. Church<br>6. Radio<br>7. Television<br>8. Newspaper or magazine<br>9. Online or internet<br>10. Others (specify)	
	Today, did any staff member at the health facility speak to you about family planning methods?*(Ngayonpo, meronpo bang tauhansa health center nakinausap kayo tingkolsapagpaplano ng pamilya?)*<br>Ngonyan po, igwa na po ban a staff hali sa health center an kinaulay kamo manungod sa pagpaplano nin pamilya?	1. Yes Meron Igwa<br>2. No Wala Mayo
	Did the health worker ask you about your concerns?*(Tinanongpoba kayo ng health worker tungkolsainyongmgaalalahanin?)*<br>Hinapot po kamo kan health worker manungod sa saindong mga alalahanon/concerns?	1. Yes<br>2. No
	Do you feel the health worker understands your concerns?<br>Sa hiling po nindo naintindihan kan health worker an saindong mga alalahanon/concerns?	1. Yes<br>2. No
	Did the health worker help you to find solutions to your concerns?<br>Nakatabang man po an health worker na masolusyonan an saindong mga alalahanon/concerns?	1. Yes<br>2. No
	Did the health worker offer you information how different family planning methods work?<br>Nasabihan o nainform po kamo kan health worker kun pano magwork an mga method?	1. Yes<br>2. No
	Which methods did health worker mention today?	

	Anong mga paagi/method ang nasabi kan health worker ngonyan?	1. Female sterilization<br>2. Male sterilization<br>3. IUD<br>4. Injectable (e.g.DMPA)<br>5. Implants<br>6. Patch<br>7. Pill<br>8. Condom<br>9. Female condom<br>10. Diaphragm<br>11. Form/Jelly/Cream<br>12. Mucus/Billings/Ovulation<br>13. Basal body temperature<br>14. Symptothermal<br>15. Standard days method<br>16. LAM<br>17. Calendar/Rhythm/Periodic abstinence<br>18. Withdrawal<br>19. Other traditional method<br>20. Other modern method (specify)
	Did the health worker tell you about side-effects or problems you might have with any methods of family planning?<br>Nasabi ba kan health worker saimo an mga posibleng side effects or problema na pwede mong makua sa mga paagi/method kan pagplano nin pamilya?	1. Yes<br>2. No
	Did the health worker offer you information how your family planning method works?<br>Nasabihan o nainform po kamo kan health worker kun pano magwork an mga method?	1. Yes<br>2. No<br>3. N/A (not using a method now)
	Did the health worker explain about the side effects of your current method?<br>Nasabi ba kan health worker saimo an mga posibleng side effects or problema na pwede mong makua sa presenteng paagi/method na ginagamit mo?	1. Yes<br>2. No
	Didthe health worker ask you to describe how you use your current method?<br>Naihapot saindo kan health worker kun pano nindo idedescribe an saindong paggamit kan presenting paagi/method na ginagamit nindo?	1. Yes<br>2. No
	After receiving FP counselling will you begin using a family planning method today?<br>Pagkatapos po kan counseling sa pagplano nin pamilya, mapuon po kamo tulos maggamit kan paagi kan pagplano nin pamilya?	1. Yes<br>2. No

	After receiving FP counselling will you begin using, do you think you will use a contraceptive method anytime in the future?<br>Pagkatapos po kan counseling sa pagplano nin pamilya, sa hiling po nindo magamit kamo nin contraceptive sa maabot na panahon?	1. Yes<br>2. No
	Which contraceptive method would you prefer to use?<br>Arin na contraceptive method an gusto nindong gamiton?	1. Female sterilization<br>2. Male sterilization<br>3. IUD<br>4. Injectable (e.g.DMPA)<br>5. Implants<br>6. Patch<br>7. Pill<br>8. Condom<br>9. Female condom<br>10. Diaphragm<br>11. Form/Jelly/Cream<br>12. Mucus/Billings/Ovulation<br>13. Basal body temperature<br>14. Symptothermal<br>15. Standard days method<br>16. LAM<br>17. Calendar/Rhythm/Periodic abstinence<br>18. Withdrawal<br>19. Other traditional method<br>20. Other modern method (specify)

	Section 4. Past Health facility visit and FP counseling<br>**Do not count today's visit.**						
401	Not including today, in the last 12 months, have you visited a health facility for care for yourself or your children for any purpose? (Kung hindiponatinisasamaangpagbisitaninyongayon, kayo poba ay nakabisitasaisang health clinic para sapangalagaananginyongkalusugan o para sainyongmgaanak at iba pang dahilan?<br>Dai ta po isasali an pagbisita nindo ngonyan, sa huring 12 bulan, nakabisita po kamo sa health center manungod sa saindong sadiring salud/kalusugan o kan saindong mga aki o maski anong dahilan?	1. Yes<br>2. No			1 -> 402<br>2 -> End of interview		
	LINE NUMBER	01	02	03	04	05	06
402	Now I would like to record all your facility visits for last 12 months. Start with the latest visit you had.<br>Why did you visit a health facility? (Ngayonpo ay itatanongkoanglahat ng pagpuntaninyosa health clinic simula January to December 2015.) Bakitpo kayo pumuntasa health clinic?<br>Ngonyan po ihahapot ko an gabos na pagbisita nindo sa health center puon January hanggang ngonyan. Tanu po kamo nagbisita/nagduman sa health center?<br>AFTER WRITING THE FIRST VISIT IN LINE NUMBER 01, ASK Q403-410 FOR THAT VISIT. THEN ASK THE 2nd LATEST VISIT TO WRITE IN 402 LINE NUMBER 02, THEN ASK Q 403 AND Q404.*(PAGKATAPOS ISULAT ANG UNANG PAGPUNTA SA HEALTH CLINIC SA UNANG LINYA, ITANONG ANG IKALAWA SA PINAKAHULING PAGPUNTA SA HEALTH CLINIC AT ISULAT SA Q402 SA BILANG 02, PAGKATAPOS ITANONG ANG Q403 AT 404).*<br>*(Pagkatapos isurat an inot na pagbisita sa health center sa inot na linya, ihapot an panduwa sa pinakhuring pagbisita sac enter tapos isurat sa Q402 sa numero 02, pagkatapos ihapot an Q403 saka 404)*<br>REPEAT FOR ALL HEALTH FACILITY VISITS FOR LAST 12 MONTHS. (*ULITIN PARA SA LAHAT NG PAGPUNTA SA HEALTH CLINIC SIMULA JANUARY 2016 – JANUARY 2017)*<br>Uliton sa gabos na pagduman sa health clinic puon January<br>IF THERE ARE MORE THAN 6, USE AN ADDITIONAL QUESTIONNAIRE.*(KUNG MAHIGIT SA ANIM, GUMAMIT NG ISA PANG QUESTIONNAIRE.)*<br>Kun sobra sa anom, mag gamit nin ibang questionnaire<br>1. Prenatal care<br>2. Giving birth, while a women is still in the facility<br>3. Health check after giving birth, after a woman left the facility<br>4. Receiving vaccination or routine check up for child						

	5. Seeking medical advice or treatment for sickness or injury of **child**<br>6. Seeking medical advice or treatment for sickness or injury of **herself**<br>7. Adolescent clinic<br>8. Other (specify)						
403	Where did you visit?*(Saanpo kayo nagpuntang health clinic?)*<br>Sain po kamo nagduman na health center?<br>1. National hospital<br>2. Regional hospital/Public medical center<br>3. Provincial hospital<br>4. District hospital<br>5. Municipal hospital<br>6. Rural health unit (RHU)/urban health center(UHC)/Lying-in<br>7. Barangay health station (BHS)<br>8. Barangay supply/service point officer/BHW<br>9. Mobile clinic<br>10.Other (specify. Private facility is included here.)						
404	At that visit, were you or your sexual partner already using any method to delay or avoid getting pregnant? (*Noongpagbisitaponinyo, kayo poba o anginyong partner aymayroonnangginanamitnapamaraan para madelay or para maiwasangmagbuntis)*<br>Kaidto pong pagduman nindo sa health center, may ginagamit nap o ba kamo na paagi/method para madelay o maiwasan an saimong pagbados?<br>1. Yes<br>2. No						
405	Which method(s) were you using?*(Alin pong pamamaraananginyongginangamit?)*<br>Arin pong mga paagi/method an ginagamit nindo?<br>WRITE DOWN ALL MENTIONED**(ISULAT LAHAT NG PAMAMARAAN)**<br><br>1. Female sterilization<br>2. Male sterilization<br>3. IUD<br>4. Injectable (e.g.DMPA)<br>5. Implants<br>6. Patch<br>7. Pill<br>8. Condom<br>9. Female condom						

	10. Diaphragm<br>11. Form/Jelly/Cream<br>12. Mucus/Billings/Ovulation<br>13. Basal body temperature<br>14. Symptothermal<br>15. Standard days method<br>16. LAM<br>17. Calendar/Rhythm/Periodic abstinence<br>18. Withdrawal<br>19. Other traditional method<br>20. Other modern method (specify)						
406	At that visit, did any staff member at the health facility speak to you about family planning methods?*(Sa inyo pong pagpuntasa health clinic, meronpo bang health staff nakumausapsainyotungkolsaparaan ng pagpaplano ng pamilya.)*<br>Kan pagduman po nindo sa health clinic, igwa po ba na health center staff na nagsabi saindo manungod sa pagplano kan pamilya?<br>1. Yes<br>2. No						
407	After that visit, did you start using any FP method or change from your previous method to a new method?*(Pagkatapospo ng inyongpagpuntasa clinic, nagpalitpobakayon ng family planning method?)*<br>Pagkatapos po nindo magduman sac enter, nagpuon na po kamo maggamit o nagribay po kamo nin paagi/method sa pagplano nin pamilya?<br>1. Yes<br>2. No						
408	If you did not start a new method or change from your previous method, why? (Kung hindinagpalit, bakit o anongdahilan?)<br>Kun dai kamo nagpuon o nagpalit nin paagi, ano ang dahilan nindo?<br>1. No need<br>2. Possible side effects of new method<br>3. New method not available at the facility<br>4. Concerns about risk of pregnancy with new method<br>5. Not enough information<br>6. Could not afford to purchase<br>7. Advice of friends, relatives, neighbours not to start or change<br>8. Husband/partner did not support<br>9. Other (specify):___________						
409	Which FP method did you start using after that visit or which new method did you change to?*(Pagkatapospo ng*						

	inyongpagpuntasa clinic, anong family planning method anginyonangginamit?)<br>Arin na paagi/method sa pagplano nin pamilya an ginamit nindo?<br>1. Female sterilization<br>2. Male sterilization<br>3. IUD<br>4. Injectable (e.g.DMPA)<br>5. Implants<br>6. Patch<br>7. Pill<br>8. Condom<br>9. Female condom<br>10. Diaphragm<br>11. Form/Jelly/Cream<br>12. Mucus/Billings/Ovulation<br>13. Basal body temperature<br>14. Symptothermal<br>15. Standard days method<br>16. LAM<br>17. Calendar/Rhythm/Periodic abstinence<br>18. Withdrawal<br>19. Other traditional method<br>20. Other modern method (specify)						

END OF THE INTERVIEW
