# Supplementary material for: Opportunities lost: Barriers to increasing the use of effective contraception in the Philippines
Source: PLoS One. 2019 Jul 25;14(7):e0218187. doi: 10.1371/journal.pone.0218187 (PMC6657820; doi:10.1371/journal.pone.0218187)
Supplement: S3 Questionnaire — (PDF) [file pone.0218187.s003.pdf]

COVER PAGE

FORM 1. Interview na las mujeres na hinde priñada o seis meses ya pasa ya pari y mujeres na no quiere anay man priñada

FORM1. Iinterview women of reproductive age who are not currently pregnant or within 6 weeks of delivery, and desire delaying or limiting childbearing

Numero:_____________

Sequence number: ______________

[Fill one number for each woman contacted in the order they were contacted at the health facility; if done over several days, continue unique sequence numbers]

Identificacion del lugar donde yan interview (Identification of interview place)		
Region (Region)		
Probinsya (Province)		
Ciudad/Municipio (CITY/MUNICIPALITY)		
BARANGAY		
Nombre de Salud oficina (Health facility name)		
Adres del Casa (Home address (for home visit only)		
Latitude and longitude (Use the coordinate of GPS in a mobile phone)		
Interview Record		
Petsa del Interview (Date of interview)		
Nombre con quen yan interview (Interviewer's name)		
Level del Facilidad del Oficina de Salud (Health facility level where interview took place)	1. National hospital<br>2. Regional hospital/Public medical center<br>3. Provincial hospital<br>4. District hospital<br>5. Municipal hospital<br>6. Rural health unit (RHU)/urban health center(UHC)/Lying-in<br>7. Barangay health station (BHS)<br>8. Barangay supply/service point officer/BHW<br>9. Mobile clinic<br>10.Other (specify)	
Clinic donde ya conducta el interview (este para na maga grande ospital) Clinic where interview took place (for large hospitals)	1. Postnatal health check after giving birth, after a woman left the facility<br>2. Receiving vaccination or routine check-up for child	

	3. Seeking medical advice or treatment for sickness or injury of child<br>4. Seeking medical advice or treatment for sickness or injury of **herself**<br>5. Adolescent clinic<br>6. Other (specify)	

Sequence Number: __________________

[Write the same sequence number from Cover Page]

Instruccion:

Le el informacion y contesta el maga question. Si el mujer quiere participa dale el certificate de consentimiento para ele pone di suyu signatura acabar puede ya prinsipia el Screening Form.

Instructions:
Read the information sheet. Answer questions. If the woman agrees to participate give the certificate of consent for her to sign. Then start the Screening Form.

Screening Form

State: “Antes came hay prinsipya tiene lang era came hay prigunta, basehan este di amon si eligible ba ustedes para na survey.

State: "We would like to start by asking a few questions that determine if you are eligible for the survey." (Uumpisahan ko na po ang pagtatanong ng ilang mga katanungan para alamin kung kayo po ay nararapat na interbyuhin para sa survey na ito.)

001	Quanto anyo tu del otro di tu yu complianyo?<br>How old were you on your last birthday? (Ilang taon napo kayo noong huling birthday?)	Age in completed years (Edad)		15-49 years ->002<br>Other -> 009
002	Prinyada ba uste ahora?<br>Are you pregnant now? (Nagdadalantao/Buntis po kayo ngayon?)	1. Yes (Oo)<br>2. No (Hindi)<br>3. Unsure (Hindi SIgurado)		1 ->009<br>2 ->003<br>3 ->003
003	Cosa el nombre del di uste anak menor?<br>What is the name of your last baby? (Ano po pangalang ng inyong pinakabatang anak?)<br>Record name	1. Name: _____________<br>2. No previous baby		1 ->004<br>2 ->006
004	Cosa mes y anyo uste ya pari con el di uste anak menor?<br>In what month and year was NAME born? (Ano po ang buwan at taon ipinanganak si __________)<br><br>(probe: when is his or her birthday) (PROBE: Kailan poang birthday ni _____________)	Month (Buwan): __ __<br><br>Year (Taon): __ __ __ __		Age ≥6 wks ->005<br>Age<6 wks ->009

005	Ya bira ba el di uste regla despues ya pari con el di uste anak menor?<br>Has your menstrual period returned since the birth of NAME? (Bumalik na po ba ng inyong regla pagkatapos manganak kay __________?)	1. Yes<br>2. No		1 ->006<br>2 ->006
006	Ahora tiene iyo question aserca na futuro. Quiere pa ba uste tiene anak y quiere pa ba ustedes omenta di ustedes anak?<br><br>Now I have some questions about the future. Would you like to have (a/another) child, or would you prefer not to have any (more) children? (Ngayon naman po, may ilan po akong katanungan tungkol sa mga darating na panahon. Gusto po ba ninyo pang magkaroon uli ng anak o mas gugustuhin ninyo na di na magkaanak?	1. Si quiere<br>Have (a/another) child (Magkaroon ulit ng anak)<br>2. Hinde, no quiere ya<br>No more/none (Hindi na)<br>3. Hinde na iyo pwde pa pari<br>Cannot get pregnant (Hindi na pwede manganak)<br>4. Nohay ideya<br>Undecided / don't know (Hindi ko alam)		1 -> 007<br>2 ->008<br>3 ->009<br>4 ->009
007	Quiere ba ustedes tiene anak dayun?<br>Do you want (a/another) child soon? (Gusto po ba ninyo na magkaanak agad?)	1. Si<br>Yes (Oo)<br>2. Hinde anay ahora<br>No, want to wait (Hindi pa, gusto ko pang maghintay)<br>3. Nohay ideya<br>Don't know (Hindi ko alam)		1 ->009<br>2 ->008<br>3- >009
008	Ustedes ba pati di uste marido ta hace manera para hinde man priñada?<br>Are you or your husband/partner currently doing something or using any method to delay or avoid getting pregnant? (Kayo po ba o ang inyong asawa/partner ay may ginagamit na paraan para madelay o maiwasan ang magbuntis?)	1. Si<br>Yes (Oo)<br>2. Hinde<br>No (Hindi)		1 -> 101<br>2 -> 101<br>To achieve a total of 5 users and non-users (hospitals) and 3 users and 3 non-users (health centres).
009	Rindi gracias con el mujer yan interview y pone di suyu nombre na lista del las mujeres con quien ya man interview y busca otro mujer con quien puede man interview.			

	Thank the woman, indicate ineligibility for the survey and stop the interview. Enter this woman into "number of women contacted". Then find another woman to interview. (Pasalamatan ang kausap, sabihan na tapos na ang interview. Ilagay ang pangalan sa listahan ng mga babae na kinausap". Maghanap ulit ng susunod na iinterviewhin.

QUESTIONNAIRE

FORM 1. Interview na las mujeres na hinde priñada o seis meses ya pasa ya pari y mujeres na no quiere anay man priñada

FORM1. Interview of women of reproductive age who are not currently pregnant or within 6 weeks of delivery, and desire delaying or limiting childbearing. *(Interbyu ng mga babae na kasalukuyan ay hindi buntis o nakapanganak na nang lampas sa anim na buwan, at nagnanais na idelay o iniiwasan ang malimit na [agbubuntis.)*

Sequence Number:
[Write the same sequence number from Cover Page]

NO.		
101	Este mes de ___________, ustedes ba ay ya queda na pueblo, ciudad, barangay o otro lugar?<br>In (month of interview) 2016, did you live in a city, in a town proper/ poblacion, in the barrio or rural area, or abroad? (Ngayon pong buwan ng ________________, kayo po ba ay tumira sa city o bayan, sa barangay o sa ibang bansa?)	1. Ciudad City (City) *(Syudad)*<br>2. City Proper TOWN PROPERIPOBLACION (*Bayan/Poblacion*)<br>3. BARRIO/RURAL AREA (Barangay)<br>4. ABROAD (*Abroad*)<br>5. Nuay ideya DON'T KNOW (Hindi alam.)
102	Uste ba ay casao o hinde casao ahora?<br>What is your marital status now? (Kayo po ba ay ___________________ sa kasalukuyan	1. Hinde casao<br>Never married or never lived with a man (*walang asawa o hindi nagkaroon ng kinakasama)*
		2. Casao<br>Currently married (*Kasalukuyang may asawa)*
		3. Live-in<br>Currently living with a man (kasalukuyang may kinakasamang lalaki)
		4. Separtao<br>Divorced/separated/widow and not currently living with a man (diborsyado/hiwalay/balo at kasalukuyang walang kinakasamang lalaki)
103	Cosa level na educacion etu ya acava?<br>What is your highest level of education attended, whether or not that level was completed? *(Ano po ang inyong natapos na antas ng pagaaral?)*	1. No education (Wala)<br>2. Elementary (Elementarya)<br>3. High school (High School)<br>4. College (Kolehiyo)<br>5. Post-graduate (Masteral o Ph D)
104	Cuanto di ustedes anak el vivo?	Number of children alive (Bilang ng anak na buhay)

	How many children do you have who are still alive? (Ilan po ang inyong buhay na anak?)	
105	Ustedes ba o tiene ba otro gente ya hace mehora para hinde na uste man priñada?<br>Did you or someone else do anything to end any of your past pregnancies? (Kayo po ba or may ibang tao ay may ginawa para sapilitang tapusin ang inyong [pagbubuntis?)	1. Si<br>Yes (Oo)<br>2. Hinde<br>No (Hindi)
106	Cuanto beses ya uste yan priñada pero ya nuay porsigi?<br>How many pregnancies did you or someone else do anything to end? *(Ilan po sa inyong pagbubuntis ang sapilitang tinapos ninyo or ng ibang tao?)*	Number of induced abortion *(Bilang ng sapilitang tinapos na pagbubuntis.)*
107	Tiene ba uste insurance de salud?<br>Are you covered by any health insurance, either as member or dependent? (*Meron po ba kayong health insurance bilang miyembro o dependent.)?*	1. Nohay Not covered (*Walang insurance*)<br>2. Philhealth<br>3. Government Service Insurance System (GSIS)<br>4. Social Security System (SSS)<br>*5.* Private insurance company/Health (maintenance organization /Pre-need insurance plan company (*Pribadong health insurance.)*<br>6. Other (Specify)(*Iba pa… pakisabi..)*
201	Di tu marido ba tiene ta usa o ta hace mehora para hine uste man priñada?<br>REVIEW: Are you or your husband/partner currently doing something or using any method to delay or avoid getting pregnant?<br>(Kayo po ba o ang inyong asawa/kapartner ay may ginagawa o may ginanagamit na pamamaraan para ipagpaliban o maiwasan na kayo ay magbuntis?)	3. Yes<br>4. No
202	Cosa mehora ustedes ta usa?<br>Which method are you currently using? (Ano pong pamamaraan o method ang inyong ginagamit?)<br><br>WRITE DOWN ALL MENTIONED.	1. Female sterilization<br>2. Male sterilization<br>3. IUD<br>4. Injectable (e.g.DMPA)<br>5. Implants<br>6. Patch<br>7. Pill<br>8. Condom<br>9. Female condom<br>10. Diaphragm<br>11. Form/Jelly/Cream<br>12. Mucus/Billings/Ovulation<br>13. Basal body temperature<br>14. Symptothermal<br>15. Standard days method<br>16. LAM

		17. Calendar/Rhythm/Periodic abstinence<br>18. Withdrawal<br>19. Other traditional method<br>20. Other modern method (specify)				
	LINE NUMBER	01	02	03	04	05
203	Ahora hay prigunta tambien came aserca na maga mehora o manera ustedes ya usa<br>Now I would like to ask you one by one about all methods you are using now. *(Ngayon naman po ay isa-isa kong tatanungin tungkol sa lahat ng pamamaraan na ginagamit ninyo ngayon.)*<br><br>RECORD ALL METHODS BEING USED NOW, ONE METHOD PER ONE LINE NUMBER.<br>IF THERE ARE MORE THAN 5 METHODS, USE ADDITIONAL QUESTIONNAIRE. *(ISULAT LAHAT NG PAMAMARAAN NA GINAGAMIT SA KASALUKUYAN, ISANG PAMAMARAAN SA BAWAT LINYA. KUNG MAY HIGIT SA LIMANG PAMAMARAAN, GUMAMIT NG ISA PANG QUESTIONNAIRE)*<br>1. Female sterilization<br>2. Male sterilization<br>3. IUD<br>4. Injectable (e.g.DMPA)<br>5. Implants<br>6. Patch<br>7. Pill<br>8. Condom<br>9. Female condom<br>10. Diaphragm<br>11. Form/Jelly/Cream<br>12. Mucus/Billings/Ovulation<br>13. Basal body temperature<br>14. Symptothermal<br>15. Standard days method<br>16. LAM<br>17. Calendar/Rhythm/Periodic abstinence<br>18. Withdrawal<br>19. Other traditional method<br>20. Other modern method (specify)					
204	Modo ya prinsipiya ustedes este usa donde ustedes este ya saca?<br>Where did you obtain that method when you first started using it? *(Nang magumpisa po kayo na gumamit ng ________ san po kayo kumuha nito.)*<br>1. National hospital<br>2. Regional hospital/Public medical center<br>3. Provincial hospital					

	4. District hospital<br>5. Municipal hospital<br>6. Rural health unit (RHU)/urban health center(UHC)/Lying-in<br>7. Barangay health station (BHS)<br>8. Barangay supply/service point officer/BHW<br>9. Mobile clinic<br>10.Other (specify. Private facility is included here)					
205	Cosa el rason cay ya ustedes na ofisina de salud?<br>What was the purpose of your going to the health facility on the day you first received the contraceptive method? *(Ano po ang dahilan at kayo ay nagpunta sa health center noong una kayong nakatanggap/gumamit ng*<br>_______________<br>1. Prenatal care<br>2. Giving birth, while a women is still in the facility<br>3. Health check after giving birth, after a woman left the facility<br>4. Receiving vaccination or routine check up for child<br>5. Seeking medical advice or treatment for sickness or injury of **child**<br>6. Seeking medical advice or treatment for sickness or injury of **herself**<br>7. Adolescent clinic<br>8. Other (specify)					
206	Si nohay ustedes cosa ta usa o ta hace mehora para hinde man priñada, tiene ba ustedes cosa ta usa y hace del una?<br>If you <u>are not</u> using any method to delay or avoid getting pregnant now, have you or your sexual partner done something or used a method to delay or avoid getting pregnant in the past? *(Kung kayo po ay hindi gumagamit nang ano mang paraan para madelay ang pagbubuntis, kayo po ba o ang inyong kapartner may ginagawa or ginagamit na pamamaraan para madelay or hindi magbuntis noong unang panahon?)*<br><br>Cosa otro mehora ustedes ta usa para hinde man priñada?<br>If <u>you are</u> using a method to delay or avoid getting pregnant now, have you or your sexual partner ever used a different method to delay or avoid getting pregnant in the past? *(Kung kayo ay gumagamit ng method o paraan para mdelay or huwag magbuntis sa ngayon, kayo ba o ang inyong kapartner ay gumamit ng ibang pamamaraan o method para madelay o hindi magbunti noong unang panahon?)*	1. Yes<br>2. No				

<table>
<tr>
<td>207</td>
<td>Cosa na este maga mehora ustedes ya usa del una maga tiempo?
Which methods have you used in the past? (Alin pong method o pamamaraan ang inyong ginamit noong nakaraang panahon?)

Iskribi aqui maga mehora
WRITE DOWN ALL MENTIONED. (Isulat lahat ng pamamaraan o method.)</td>
<td colspan="6">1. Female sterilization
2. Male sterilization
3. IUD
4. Injectable (e.g.DMPA)
5. Implants
6. Patch
7. Pill
8. Condom
9. Female condom
10. Diaphragm
11. Form/Jelly/Cream
12. Mucus/Billings/Ovulation
13. Basal body temperature
14. Symptothermal
15. Standard days method
16. LAM
17. Calendar/Rhythm/Periodic abstinence
18. Withdrawal
19. Other traditional method
20. Other modern method (specify)</td>
</tr>
<tr>
<td></td>
<td>LINE NUMBER</td>
<td>01</td>
<td>02</td>
<td>03</td>
<td>04</td>
<td>05</td>
<td>06</td>
</tr>
<tr>
<td>208</td>
<td>Ahora hay prigunta tambien iyo aserca na maga manera ustedes ya usa del una.
Now I would like to ask you one by one about all methods you have used in the past. (Ngayon naman po ay isa-isa kong tatanungin tungkol sa lahat ng pamamaraan na ginagamit ninyo noong nakaraang panahon).

RECORD ALL METHODS, ONE METHOD PER ONE LINE NUMBER. (ISULAT LAHAT NG PAMAMARAAN NA GINAGAMIT NOONG NAKARAAN, ISANG PAMAMARAAN SA BAWAT LINYA.)

IF THERE ARE MORE THAN 5 METHODS, USE ADDITIONAL QUESTIONNAIRE. (KUNG MAY HIGIT SA LIMANG PAMAMARAAN, GUMAMIT NG ISA PANG QUESTIONNAIRE)

1. Female sterilization
2. Male sterilization
3. IUD
4. Injectable (e.g.DMPA)
5. Implants
6. Patch
7. Pill
8. Condom
9. Female condom
10. Diaphragm
11. Form/Jelly/Cream</td>
<td></td>
<td></td>
<td></td>
<td></td>
<td></td>
<td></td>
</tr>
</table>

	12. Mucus/Billings/Ovulation<br>13. Basal body temperature<br>14. Symptothermal<br>15. Standard days method<br>16. LAM<br>17. Calendar/Rhythm/Periodic abstinence<br>18. Withdrawal<br>19. Other traditional method<br>20. Other modern method (specify)						
209	Aquel maga tiempo ya usa ustedes donde ustedes este ya saca?<br>Where did you obtain the family planning method when you first started using it? *(Nang magumpisa po kayo na gumamit ng ________ san po kayo kumuha nito.)*<br><br>1. National hospital<br>2. Regional hospital/Public medical center<br>3. Provincial hospital<br>4. District hospital<br>5. Municipal hospital<br>6. Rural health unit (RHU)/urban health center(UHC)/Lying-in<br>7. Barangay health station (BHS)<br>8. Barangay supply/service point officer/BHW<br>9. Mobile clinic<br>10.Other (specify. Private facility is included here.)						
210	Cosa el rason ya usa ustedes family planning?<br>Why did you visit the health facility where you first started using the family planning method? *(Ano po ang dahilan at kayo ay nagpunta sa health center noong una kayong nakatanggap/gumamit ng* __________________<br><br>1. Prenatal care<br>2. Giving birth, while still in the facility<br>3. Health check after giving birth, after leaving the facility<br>4. Receiving vaccinations or routine check-ups for a child<br>5. Seeking medical advice or treatment for sickness or injury of a **child**<br>6. Seeking medical advice or treatment for sickness or injury of **herself**<br>7. Adolescent clinic<br>8. Other (specify)						
211	Porque ustedes ya descansa usa mehora family planning?<br>Why did you stop using the family planning method that you used in the past? (Bakit po ninyo itinigil ang paggamit ng<br>__________________________<br><br>1. Side effects						

	2. Method not available at the facility<br>3. Concerns about risks of pregnancy<br>4. Could not afford to purchase<br>5. Health worker did not continue to provide the method<br>6. Advice of friends, relatives, neighbors<br>7. Husband/partner did not support<br>8. Wanted to get pregnant<br>9. Other (specify):___________						

	Section 3. FP Concerns and Today's FP counseling						
301	Tiene ba ustedes concierne aserca na maga diferente mehora de family planning?<br>Do you have any health concerns about any type of family planning method?	1. Yes<br>2. No					
	LINE NUMBER	01	02	03	04	05	06
302	Cosa cosa maga concierne de salud ustedes tiene aserca de family planning?<br>What are your health concerns about family planning methods*? (Ano-ano po ang inyong mga alalahaning ipekto ipekto sa kalusugan ng bawat pamamaraan?)*<br>Please tell me one by one. *(Pakisabi po ninyo ang bawat isa.)*<br><br>USE ONE LINE NUMBER FOR ONE CONCERN. WRITE DOWN ALL MENTIONED CONCERNS. (Isang linya sa bawat alalahanin. Isulat ang lahat na alalahanin.)<br>IF THERE ARE MORE THAN 6 CONCERNS, USE ADDITIONAL QUESTIONNAIRE. *KUNG MAY HIGIT SA ANIM NA PAMAMARAAN, GUMAMIT NG ISA PANG QUESTIONNAIRE)*<br>1. Cause cancer in the uterus<br>2. Cause cysts in the uterus<br>3. Cause infection of the uterus<br>4. Cause frequent bleeding<br>5. Cause thyroid problems<br>6. Cause/worse asthma<br>7. Cause/worse lots of veins<br>8. Cause dry skin, skin disease<br>9. Cause edema<br>10. Cause weight gain<br>11. Cause weight loss<br>12. Cause bloated stomach<br>13. Cause headache<br>14. Cause irritability<br>15. Increase libido/turn into a maniac<br>16. Cause loss/reduce of libido<br>17. Cause loss/reduce of sexual satisfaction<br>18. One will not have children anymore<br>19. Not fully effective, woman could still get pregnant<br>20. When it does not work, the baby is born with abnormalities<br>21. Results in mortal sin because it is against church teachings<br><br>**IUD/Implants**						

	22. Melt or move around inside the body and doctors will not be able to find<br>23. Washed away/pushed out of body<br>24. Painful to insert<br><br>**IUD**<br>25. Itchy on the vagina<br>26. Entangled around the man's penis<br>27. Messy when inserted<br><br>**Male sterilization**<br>28. Part of the man's testicles are cut off<br>29. It hurts the testicles<br>30. The man loses his manhood ("kapon")<br><br>31. Others (specify)						
303	Cosa na maga family planning tiene ustedes concierne o tan alang-alang?<br>About which family planning methods do you have concerns? (Alin pong pagpaplano ng pamilya ang meron kayong alalahanin o agam-agam?)<br><br>REPEAT EACH CONCERN IN TURN. FOR EACH CONCERN, WRITE DOWN ALL METHODS CAUSING THAT CONCERN. *(ULITIN ANG BAWAT ALALAHANIN. SA BAWAT ALALAHANIN, ISULAT LAHAT NG PARAAN NG PAGPAPLANO NG PAMILYA.)*<br><br>1. Female sterilization<br>2. Male sterilization<br>3. IUD<br>4. Injectable<br>5. Implants<br>6. Patch<br>7. Pill<br>8. Other modern method (specify)<br>9. Other method (specify)						
304	Quien con ustedes ya informa aserca de este family planning?<br>Who told you or how did you find about your concerns about family planning methods? *(Sino po ang nagsabi sa inyo o paano kayo nagkaroon ng agam-agam o alalahanin tungkol sa pamaraan ng pagpaplano ng pamilya.)*						

	REPEAT EACH CONCERN IN TURN. FOR EACH WRITE DOWN ALL SOUCES OF INFORMATION. (ULITIN ANG BAWAT ALALAHANIN. SA BAWAT PAMAMARAAN, ISULAT ANG BAWAT PINAGMULAN NG INPORMASYON)<br>1. Health staff<br>2. BHW or health volunteers<br>3. Marido Husband or partner<br>4. Amigo/amiga, vecinos, Friend, neighbours, relatives<br>5. Iglesia Church<br>6. Radio<br>7. Television<br>8. Newspaper or magazine<br>9. Online or internet<br>10. Others (specify)	
305	Este tiempo, tiene ba de maga empleyado de ofisina de salud ya informa con ustedes aserca de este mehora de family planning?<br>Today, did any staff member at the health facility speak to you about family planning methods? *(Ngayon po, meron po bang tauhan sa health center na kinausap kayo tingkol sa pagpaplano ng pamilya?)*	1. Yes<br>2. No
306	Ya prigunta ba el maga empleyado de ofisina de salud aserca del di ustedes concierne ?<br>Did the health worker ask you about your concerns? *(Tinanong po ba kayo ng health worker tungkol sa inyong mga alalahanin?)*	1. Yes<br>2. No
307	Ta sinti ba ustedes cay ta intende con ustedes maga empleyado de ofisina de salud aserca del di ustedes concierne ?<br>Do you feel the health worker understands your concerns?	1. Yes<br>2. No
308	Tiene ba sila ya dale con ustedes solucion aserca del di ustedes concierne ?<br>Did the health worker help you to find solutions to your concerns?	1. Yes<br>2. No
309	Ya informa ba con ustedes el maga empleyado de ofisina de salud aserca del maga diferente mehora del family planning?<br>Did the health worker offer you information how different family planning methods work?	1. Yes<br>2. No
310	Cosa mehora el maga empleyado de ofisina de salud ya menciona aserca ahora?	1. Female sterilization

	Which methods did health worker mention today?	2. Male sterilization<br>3. IUD<br>4. Injectable (e.g.DMPA)<br>5. Implants<br>6. Patch<br>7. Pill<br>8. Condom<br>9. Female condom<br>10. Diaphragm<br>11. Form/Jelly/Cream<br>12. Mucus/Billings/Ovulation<br>13. Basal body temperature<br>14. Symptothermal<br>15. Standard days method<br>16. LAM<br>17. Calendar/Rhythm/Periodic abstinence<br>18. Withdrawal<br>19. Other traditional method<br>20. Other modern method (specify)
311	Ya esplica ba con ustedes maga empleyado de ofisina de salud el maga efecto o problema na maga mehora de family planning?<br>Did the health worker tell you about side-effects or problems you might have with any methods of family planning?	1. Yes<br>2. No
312	Ya esplica ba con ustedes maga empleyado de ofisina de salud que modo ta usa maga mehora de family planning?<br>Did the health worker offer you information how your family planning method works?	1. Yes<br>2. No<br>3. N/A (not using a method now)
313	Ya esplica ba con ustedes maga empleyado de ofisina de salud el maga efecto o problema na maga mehora de family planning ya usa ustedes?<br>Did the health worker explain about the side effects of your current method?	1. Yes<br>2. No
314	Ya prigunta ba con ustedes maga empleyado de ofisina de salud si quemodo ustedes ya usa el maga mehora de family planning?<br>Did the health worker ask you to describe how you use your current method?	1. Yes<br>2. No
315	Acabar ustedes resibi este conselo de FP, hay usa ya ba ustedes ahora este maga mehora de family planning?<br>After receiving FP counselling will you begin using a family planning method today?	1. Yes<br>2. No

316	Acabar ustedes resibi este conselo de FP, hay usa ya ba ustedes este llama contraceptivo na otro tiempo?<br>After receiving FP counselling will you begin using, do you think you will use a contraceptive method anytime in the future?	1. Yes<br>2. No
317	Cosa clase contraceptivo ustedes ay ta pensa usa?<br>Which contraceptive method would you prefer to use?	1. Female sterilization<br>2. Male sterilization<br>3. IUD<br>4. Injectable (e.g.DMPA)<br>5. Implants<br>6. Patch<br>7. Pill<br>8. Condom<br>9. Female condom<br>10. Diaphragm<br>11. Form/Jelly/Cream<br>12. Mucus/Billings/Ovulation<br>13. Basal body temperature<br>14. Symptothermal<br>15. Standard days method<br>16. LAM<br>17. Calendar/Rhythm/Periodic abstinence<br>18. Withdrawal<br>19. Other traditional method<br>20. Other modern method (specify)

	Section 4. Past Health facility visit and FP counseling<br>**Do not count today's visit.**						
401	Puero este tiempo, ustedes ba ay ya bisita na clinica para manda chequia y di ustedes maga anak?<br>Not including today, in the last 12 months, have you visited a health facility for care for yourself or your children for any purpose? (Kung hindi po natin isasama ang pagbisita ninyo ngayon, kayo po ba ay nakabisita sa isang health clinic para sa pangalagaan ang inyong kalusugan o para sa inyong mga anak at iba pang dahilan?	1. Yes<br>2. No			1 -> 402<br>2 -> End of interview		
	LINE NUMBER	01	02	03	04	05	06
402	Cosa el rason rason ya bisita ustedes na clinica?<br>Now I would like to record all your facility visits for last 12 months. Start with the latest visit you had.<br>Why did you visit a health facility? (Ngayon po ay itatanong ko ang lahat ng pagpunta ninyo sa health clinic simula January to December 2015.) Bakit po kayo pumunta sa health clinic?<br><br>AFTER WRITING THE FIRST VISIT IN LINE NUMBER 01, ASK Q403-410 FOR THAT VISIT. THEN ASK THE 2nd LATEST VISIT TO WRITE IN 402 LINE NUMBER 02, THEN ASK Q 403 AND Q404. *(PAGKATAPOS ISULAT ANG UNANG PAGPUNTA SA HEALTH CLINIC SA UNANG LINYA, ITANONG ANG IKALAWA SA PINAKAHULING PAGPUNTA SA HEALTH CLINIC AT ISULAT SA Q402 SA BILANG 02, PAGKATAPOS ITANONG ANG Q403 AT 404).*<br>REPEAT FOR ALL HEALTH FACILITY VISITS FOR LAST 12 MONTHS. (*ULITIN PARA SA LAHAT NG PAGPUNTA SA HEALTH CLINIC SIMULA JANUARY 2016 – JANUARY 2017)*<br>IF THERE ARE MORE THAN 6, USE AN ADDITIONAL QUESTIONNAIRE. *(KUNG MAHIGIT SA ANIM, GUMAMIT NG ISA PANG QUESTIONNAIRE.)*<br><br>1. Prenatal care<br>2. Giving birth, while a women is still in the facility<br>3. Health check after giving birth, after a woman left the facility<br>4. Receiving vaccination or routine check up for child<br>5. Seeking medical advice or treatment for sickness or injury of **child**<br>6. Seeking medical advice or treatment for sickness or injury of **herself**<br>7. Adolescent clinic<br>8. Other (specify)						
403	Donde ustedes ofisina de salud ya bisita?						

	Where did you visit? *(Saan po kayo nagpuntang health clinic?)*<br><br>1. National hospital<br>2. Regional hospital/Public medical center<br>3. Provincial hospital<br>4. District hospital<br>5. Municipal hospital<br>6. Rural health unit (RHU)/urban health center(UHC)/Lying-in<br>7. Barangay health station (BHS)<br>8. Barangay supply/service point officer/BHW<br>9. Mobile clinic<br>10.Other (specify. Private facility is included here.)						
404	Modo ustedes ya bisita na clinica, ustedes ba hay ta usa ya con el maga mehora de family planning para hinde man priñada?<br>At that visit, were you or your sexual partner already using any method to delay or avoid getting pregnant? (*Noong pagbisita po ninyo, kayo po ba o ang inyong partner ay mayroon nang ginanamit na pamaraan para madelay or para maiwasang magbuntis)*<br><br>1. Yes<br>2. No						
405	Cosa del maga mehora de family planning ustedes ya usa?<br>Which method(s) were you using? *(Alin pong pamamaraan ang inyong ginangamit?)*<br><br>WRITE DOWN ALL MENTIONED **(ISULAT LAHAT NG PAMAMARAAN)**<br><br>1. Female sterilization<br>2. Male sterilization<br>3. IUD<br>4. Injectable (e.g.DMPA)<br>5. Implants<br>6. Patch<br>7. Pill<br>8. Condom<br>9. Female condom<br>10. Diaphragm<br>11. Form/Jelly/Cream<br>12. Mucus/Billings/Ovulation<br>13. Basal body temperature<br>14. Symptothermal						

	15. Standard days method<br>16. LAM<br>17. Calendar/Rhythm/Periodic abstinence<br>18. Withdrawal<br>19. Other traditional method<br>20. Other modern method (specify)						
406	El tiempo ustedes ya bisita na clinica, tiene ba del maga empleyado del ofisina del salud ya informa con ustedes maga diferente mehora de family planning?<br>At that visit, did any staff member at the health facility speak to you about family planning methods? *(Sa inyo pong pagpunta sa health clinic, meron po bang health staff na kumausap sa inyo tungkol sa paraan ng pagpaplano ng pamilya.)*<br><br>1. Yes<br>2. No						
407	Acabar ustedes ya bisita na clinica, ya cambia ba el di ustedes mehora de family planning?<br>After that visit, did you start using any FP method or change from your previous method to a new method? *(Pagkatapos po ng inyong pagpunta sa clinic, nagpalit po ba kayon ng family planning method?)*<br><br>1. Yes<br>2. No						
408	Si nohay ustedes usa cosa manera o cambia cosa el rason?<br>If you did not start a new method or change from your previous method, why? (Kung hindi nagpalit, bakit o anong dahilan?)<br><br>1. No need<br>2. Possible side effects of new method<br>3. New method not available at the facility<br>4. Concerns about risk of pregnancy with new method<br>5. Not enough information<br>6. Could not afford to purchase<br>7. Advice of friends, relatives, neighbours not to start or change<br>8. Husband/partner did not support<br>9. Other (specify):___________						
409	Acabar ustedes ya bisita na clinica, cosa del maga mehora de family planning ustedes ya usa?<br>Which FP method did you start using after that visit or which new method did you change to? *(Pagkatapos po ng inyong pagpunta sa clinic, anong family planning method ang inyo nang ginamit?)*						

	1. Female sterilization<br>2. Male sterilization<br>3. IUD<br>4. Injectable (e.g.DMPA)<br>5. Implants<br>6. Patch<br>7. Pill<br>8. Condom<br>9. Female condom<br>10. Diaphragm<br>11. Form/Jelly/Cream<br>12. Mucus/Billings/Ovulation<br>13. Basal body temperature<br>14. Symptothermal<br>15. Standard days method<br>16. LAM<br>17. Calendar/Rhythm/Periodic abstinence<br>18. Withdrawal<br>19. Other traditional method<br>20. Other modern method (specify)						

END OF THE INTERVIEW
