# Supplementary material for: Opportunities lost: Barriers to increasing the use of effective contraception in the Philippines
Source: PLoS One. 2019 Jul 25;14(7):e0218187. doi: 10.1371/journal.pone.0218187 (PMC6657820; doi:10.1371/journal.pone.0218187)
Supplement: S4 Questionnaire — (PDF) [file pone.0218187.s004.pdf]

COVER PAGE

FORM1. Interview women of reproductive age who are not currently pregnant or within 6 weeks of delivery, and desire delaying or limiting childbearing

Sequence number: _____________

[Fill one number for each woman contacted in the order they were contacted at the health facility; if done over several days, continue unique sequence numbers]

Identification of interview place		
Region		
Province		
CITY/MUNICIPALITY		
BARANGAY		
Health facility name		
Home address (for home visit only)		
Latitude and longitude<br>(Use the coordinate of GPS in a mobile phone)		
Interview Record		
Date of interview		
Interviewer's name		
Health facility level where interview took place	1. National hospital<br>2. Regional hospital/Public medical center<br>3. Provincial hospital<br>4. District hospital<br>5. Municipal hospital<br>6. Rural health unit (RHU)/urban health center(UHC)/Lying-in<br>7. Barangay health station (BHS)<br>8. Barangay supply/service point officer/BHW<br>9. Mobile clinic<br>10. Other (specify)	
Clinic where interview took place (for large hospitals)	1. Postnatal health check after giving birth, after a woman left the facility ***(Check-up kalpasan nga nagganak ken awan jay babae jay health facility)***<br>2. Receiving vaccination or routine check-up for child ***(Makaala iti bakuna para ti babassit pay nga anak)***<br>3. Seeking medical advice or treatment for sickness or injury of child ***(Agpaagas wenno agpakita iti doctor para ti masaksakit wenno nadunor nga anak)***	

	4. Seeking medical advice or treatment for sickness or injury of **herself** ***(Agpaagas wenno agpakita iti doktor para ti bagbagi)***<br>5. Adolescent clinic<br>6. Other (specify) ***Sabali pay***	

Sequence Number: _________________
[Write the same sequence number from Cover Page]

Instructions:
Read the information sheet. Answer questions. If the woman agrees to participate give the certificate of consent for her to sign. Then start the Screening Form.

Screening Form

State: "We would like to start by asking a few questions that determine if you are eligible for the survey."

001	How old were you on your last birthday?<br><br>***Mano ti tawen mo idi napalabas nga birthday mo?***	Age in completed years		15-49 years ->002<br>Other -> 009
002	Are you pregnant now?<br><br>***Masikog ka kadi tatta?***	1. Yes<br>2. No<br>3. Unsure<br><br>***1. Wen***<br>***2. Haan***<br>*3.* ***Haan nga sigurado***		1 ->009<br>2 ->003<br>3 ->003
003	What is the name of your last baby?<br><br>***Anya ti nagan na ti buridek nga anak mo?***<br><br>Record name	1. Name: ____________<br>2. No previous baby (***Awan ti naudi nga anak)***		1 ->004<br>2 ->006
004	In what month and year was NAME born?<br><br>***Kaano nga naiyanak diyay anak mo nga buridek? Anya nga bulan ken tawen?***<br><br>(probe: when is his or her birthday)	Month: __ __<br><br>Year: __ __ __ __		Age ≥6 wks ->005<br>Age<6 wks ->009
005	Has your menstrual period returned since the birth of NAME?<br><br>***Nagsubli kadi iti reglam manipud idi inyanak mo diyay buridek mo?***	1. Yes<br>2. No<br><br>***1. Wen***<br>*2.* ***Haan***		1 ->006<br>2 ->006

006	Now I have some questions about the future. Would you like to have (a/another) child, or would you prefer not to have any (more) children?<br><br>***Kayat mo pay kadi iti aganak, wenno kayat mo pay iti adu nga anak?***	1. Have (a/another) child<br>2. No more/none<br>3. Cannot get pregnant<br>4. Undecided / don't know<br><br>**1.** ***Kayat pay iti mesa nga anak***<br>**2.** ***Haan***<br>**3.** ***Haan nga makasikog***<br>4. ***Haan nga sigurado***		1 -> 007<br>2 ->008<br>3 ->009<br>4 ->009
007	Do you want (a/another) child soon?<br><br>***Kayat mo kadi iti aganak to manen?***	1. Yes<br>2. No, want to wait<br>3. Don't know<br><br>**1.** ***Wen***<br>**2.** ***Haan, kayat ko pay iti aguray***<br>3. ***Haan ko ammo***		1 ->009<br>2 ->008<br>3- >009
008	Are you or your husband/partner currently doing something or using any method to delay or avoid getting pregnant?<br><br>***Adda kadi ub-ubraen na diyay asawam tapno haan ka agsikog wenno haan nga agsikog insigida?***	1. Yes<br>2. No<br><br>***1. Wen***<br>2. ***Haan***		1 -> 101<br>2 -> 101<br>To achieve a total of 5 users and non-users (hospitals) and 3 users and 3 non-users (health centres).
009	Thank the woman, indicate ineligibility for the survey and stop the interview. Enter this woman into "number of women contacted". Then find another woman to interview.			

QUESTIONNAIRE

FORM1. Interview of women of reproductive age who are not currently pregnant or within 6 weeks of delivery, and desire delaying or limiting childbearing

Sequence Number:
[Write the same sequence number from Cover Page]

NO.	Section 1. Respondent background			
101	In (month of interview) 2016, did you live in a city, in a town proper/ poblacion, in the barrio or rural area, or abroad?<br><br>***Idi na-interview ka, nag-giyanam idi? Ayan na diyay balay yo? Nag giyan ka ba iti ili wenno poblacion? Barrio wenno taga abroad ka?***	1. City<br>2. TOWN PROPERIPOBLACION<br>3. BARRIO/RURAL AREA<br>4. ABROAD<br>5. DON'T KNOW ***(Haan ko ammo)***		->102
102	What is your marital status now?<br><br>***Naikasar ka kadi?***	1. Never married or never lived with a man<br>2. Currently married<br>3. Currently living with a man<br>4. Divorced/separated/widow and not currently living with a man<br><br>**1.** ***Haan nga naikasar wenno naki-live in***<br>**2.** ***Naikasar***<br>**3.** ***Madama nga maki-live-in.***<br>4. ***Dibursiyada/Nagsina/ Biyuda ken awan kadkadwaaen na nga lalaki***		-> 103
103	What is your highest level of education attended, whether or not that level was completed?<br><br>***Anya iti kangatwan nga nag-adalam?***	1. No education<br>2. Elementary<br>3. High school<br>4. College<br>5. Post-graduate<br><br>**1.** ***Haan nga nakaadal***<br>**2.** ***Elementary***<br>**3.** ***High School***		->104

		**4. *College***<br>5. ***Post-graduate***		
104	How many children do you have who are still alive?<br><br>***Mano iti anak mo tatta nga sibibiyag?***	Number of children alive		->106
105	Did you or someone else do anything to end any of your past pregnancies?<br><br>***Adda kadi inubram wenno inpaaramid mo iti sabali tapno maagasan ka?***	1. Yes<br>2. No<br><br>**1. *Wen***<br>2. ***Haan***		1 ->107<br>2 -> 108
106	How many pregnancies did you or someone else do anything to end?<br><br>***Mano nga pagsikog iti inpaagas mo wenno insardeg mo?***	Number of induced abortion		->108
107	Are you covered by any health insurance, either as member or dependent?<br><br>***Ada kadi ti health insurance mo wenno miyembro ka? Dependent ka kadi iti mesa nga health insurance?***	1. Not covered<br>2. Philhealth<br>3. Government Service Insurance System<br>4. Social Security System<br>5. Private insurance company/Health maintenance organization /Pre-need insurance plan company<br>6. Other (Specify)<br><br>**1. *Awan insurance na***<br>**2. *Philhealth***<br>**3. *GSIS***<br>**4. *SSS***<br>**5. *Private insurance company***<br>6. ***Sabali***		->201

NO.	Section 2. Current use of FP			
201	REVIEW: Are you or your husband/partner currently doing something or using any	1. Yes<br>2. No<br><br>***1. Wen***		1 -> 202<br>2 -> 206

	method to delay or avoid getting pregnant?<br><br>***Adda kadi ub-ubraen na diyay asawam tatta tapno haan ka nga agsikog insigida?***	*2.* ***Haan***					
202	Which method are you currently using?<br><br>***Anya dagijay methods nga us-usaren yo tatta?***<br><br>WRITE DOWN ALL MENTIONED.	1. Female sterilization<br>2. Male sterilization<br>3. IUD<br>4. Injectable (e.g.DMPA)<br>5. Implants<br>6. Patch<br>7. Pill<br>8. Condom<br>9. Female condom<br>10. Diaphragm<br>11. Form/Jelly/Cream<br>12. Mucus/Billings/Ovulation<br>13. Basal body temperature<br>14. Symptothermal<br>15. Standard days method<br>16. LAM<br>17. Calendar/Rhythm/Periodic abstinence<br>18. Withdrawal<br>19. Other traditional method<br>20. Other modern method (specify)					-> 203
	LINE NUMBER	01	02	03	04	05	
203	Now I would like to ask you one by one about all methods you are using now.<br><br>***Damagen ka mesa mesa manipud dagiti us-usarem tatta.***<br><br>RECORD ALL METHODS BEING USED NOW, ONE METHOD PER ONE LINE NUMBER.<br>IF THERE ARE MORE THAN 5 METHODS, USE ADDITIONAL QUESTIONNAIRE.<br><br>1. Female sterilization<br>2. Male sterilization<br>3. IUD						->204

	4. Injectable (e.g.DMPA)<br>5. Implants<br>6. Patch<br>7. Pill<br>8. Condom<br>9. Female condom<br>10. Diaphragm<br>11. Form/Jelly/Cream<br>12. Mucus/Billings/Ovulation<br>13. Basal body temperature<br>14. Symptothermal<br>15. Standard days method<br>16. LAM<br>17. Calendar/Rhythm/Periodic abstinence<br>18. Withdrawal<br>19. Other traditional method<br>20. Other modern method (specify)						
204	Where did you obtain that method when you first started using it?<br><br>***Idi nangrugi ka nga nag-usar iti family planning method, nagalam dagitoy?***<br><br>1. National hospital<br>2. Regional hospital/Public medical center<br>3. Provincial hospital<br>4. District hospital<br>5. Municipal hospital<br>6. Rural health unit (RHU)/urban health center(UHC)/Lying-in<br>7. Barangay health station (BHS)<br>8. Barangay supply/service point officer/BHW<br>9. Mobile clinic<br>10. Other (specify. Private facility is included here)						-> 205
205	What was the purpose of your going to the health facility on						-> 203. Repeat until all methods

	the day you first received the contraceptive method?<br><br>***Anya ti rason na nu apay nga napan ka diyay health facility idi nagrugi ka nga mangusar iti contraceptive method?***<br><br>1. Prenatal care ***(Check-up sakbay nga aganak)***<br>2. Giving birth, while a women is still in the facility ***(Manganak pay lang jay babae ken adidjay pa lang health facility)***<br>3. Health check after giving birth, after a woman left the facility ***(Check-up kalpasan nga nagannak jay babe ngem awan suna jay health facility)***<br>4. Receiving vaccination or routine check up for child ***(Makaala iti bakuna para ti babassit pay nga anak)***<br>5. Seeking medical advice or treatment for sickness or injury of **child** ***(Agpaagas wenno agpakita iti doctor para ti masaksakit wenno nadunor nga anak)***<br>6. Seeking medical advice or treatment for sickness or injury of **herself** ***(Agpaagas wenno agpakita iti doktor para ti bagbagi)***<br>7. Adolescent clinic<br>8. Other (specify) ***Sabali pay***						were explained.<br><br>Then ->206
206	If you <u>are not</u> using any method to delay or avoid getting pregnant now, have you or your sexual partner done something or used a method to delay or avoid getting pregnant in the past?	1. Yes<br>2. No<br><br>***1. Wen***<br>2. ***Haan***					1->207<br>2-> 301

	Nu haan ka tatta nga ag-us-usar iti uray anya tapno haan ka nga agsikog insigida, adda kadi inusar mo idi?<br><br>If you are using a method to delay or avoid getting pregnant now, have you or your sexual partner ever used a different method to delay or avoid getting pregnant in the past?<br><br>***Nu ag-us-usar ka tatta iti contraceptive method, nag-usar ka ba idi?***						
207	Which methods have you used in the past?<br><br>***Anya dagitoy nga inus-usar mo idi?***<br><br>WRITE DOWN ALL MENTIONED.	1. Female sterilization<br>2. Male sterilization<br>3. IUD<br>4. Injectable (e.g.DMPA)<br>5. Implants<br>6. Patch<br>7. Pill<br>8. Condom<br>9. Female condom<br>10. Diaphragm<br>11. Form/Jelly/Cream<br>12. Mucus/Billings/Ovulation<br>13. Basal body temperature<br>14. Symptothermal<br>15. Standard days method<br>16. LAM<br>17. Calendar/Rhythm/Periodic abstinence<br>18. Withdrawal<br>19. Other traditional method<br>20. Other modern method (specify)					-> 208
	LINE NUMBER	01	02	03	04	05	
208	Now I would like to ask you one by one about all methods you have used in the past<br><br>***Damagen ka dagiti inus-usar mo idi.***						->209

	RECORD ALL METHODS, ONE METHOD PER ONE LINE NUMBER.<br>IF THERE ARE MORE THAN 5 METHODS, USE ADDITIONAL QUESTIONNAIRE.<br><br>1. Female sterilization<br>2. Male sterilization<br>3. IUD<br>4. Injectable (e.g.DMPA)<br>5. Implants<br>6. Patch<br>7. Pill<br>8. Condom<br>9. Female condom<br>10. Diaphragm<br>11. Form/Jelly/Cream<br>12. Mucus/Billings/Ovulation<br>13. Basal body temperature<br>14. Symptothermal<br>15. Standard days method<br>16. LAM<br>17. Calendar/Rhythm/Periodic abstinence<br>18. Withdrawal<br>19. Other traditional method<br>20. Other modern method (specify)						
209	Where did you obtain the family planning method when you first started using it?<br><br>***Nagalam dagiti family planning method nga inusar mo idi?***<br><br>1. National hospital<br>2. Regional hospital/Public medical center<br>3. Provincial hospital<br>4. District hospital<br>5. Municipal hospital<br>6. Rural health unit (RHU)/urban health center(UHC)/Lying-in<br>7. Barangay health station (BHS)						->210

	8. Barangay supply/service point officer/BHW<br>9. Mobile clinic<br>10. Other (specify. Private facility is included here.)						
210	Why did you visit the health facility where you first started using the family planning method?<br><br>***Apay nga napan ka diyay health facility idi nangrugi ka nga ag-usar iti family planning method?***<br><br>1. Prenatal care ***(Check-up sakbay nga aganak)***<br>2. Giving birth, while still in the facility ***(Manganak pay lang jay babae ken adidjay pa lang health facility)***<br>3. Health check after giving birth, after leaving the facility ***(Check-up kalpasan nga nagannak jay babe ngem awan suna jay health facility)***<br>4. Receiving vaccinations or routine check-ups for a child ***(Agpabakuna ken check up para iti bassit nga anak)***<br>5. Seeking medical advice or treatment for sickness or injury of a **child** ***(Agpaagas wenno agpakita iti doctor para ti masaksakit wenno nadunor nga anak)***<br>6. Seeking medical advice or treatment for sickness or injury of **herself** ***(Agpaagas wenno agpakita iti doktor para ti bagbagi)***<br>7. Adolescent clinic<br>8. Other (specify) ***Sabali pay***						-> 211

211	Why did you stop using the family planning method that you used in the past?<br><br>***Apay nga insardeng mo nga usaren dagijay family planning method nga inusar mo idi?***<br><br>1. Side effects<br>2. Method not available at the facility ***(Awan iti available dijay facility)***<br>3. Concerns about risks of pregnancy ***(Adu ti damdamag ken baka delikado iti panagsikog)***<br>4. Could not afford to purchase ***(Haan ko kaya nga gumatang)***<br>5. Health worker did not continue to provide the method ***(Haan nga nangited nagijay health workers)***<br>6. Advice of friends, relatives, neighbors ***(Isu iti kuna dagijay gagayem)***<br>***7.*** Husband/partner did not support ***(Haan nga sinupotaan jay asawak)***<br>8. Wanted to get pregnant ***(Kayat ko iti agsikog)***<br>9. Other (specify):__________						->212

	**Section 3. FP Concerns and Today's FP counseling**							
301	Do you have any health concerns about any type of family planning method?<br><br>***Adda kadi iti kayat mo nga maamwan gapu iti klase iti family planning method?***	1. Yes<br>2. No<br><br>***1. Wen***<br>2. ***Haan***						1 ->302<br>2 ->305
	LINE NUMBER	01	02	03	04	05	06	
302	What are your health concerns about family planning methods? Please tell me one by one.<br><br>***Anya dagiti damdamag mo wenno kayat mo nga maamwan gapu iti family planning methods? Ibagam kanyak mesa mesa.***<br><br>USE ONE LINE NUMBER FOR ONE CONCERN. WRITE DOWN ALL MENTIONED CONCERNS. IF THERE ARE MORE THAN 6 CONCERNS, USE ADDITIONAL QUESTIONNAIRE.<br><br>1. Cause cancer in the uterus ***(Maka-cancer jay uterus)***<br>2. Cause cysts in the uterus ***(Maka-cyst dijay uterus)***<br>3. Cause infection of the uterus ***(Baka agka-infection dijay uterus)***<br>4. Cause frequent bleeding ***(Baka agdara)***<br>5. Cause thyroid problems ***(Baka agkaproblema iti thyroid)***<br>6. Cause/worse asthma ***(Baka kumaro iti angkit)***<br>7. Cause/worse lots of veins<br>8. Cause dry skin, skin disease ***(Baka agsakit diyay kudil)***							-> 303

	9. Cause edema ***(Baka lumteg)***<br>10. Cause weight gain ***(Baka dumagsen ka)***<br>11. Cause weight loss ***(Baka lumag-an ka)***<br>12. Cause bloated stomach ***(Baka agpadakkel iti tian)***<br>13. Cause headache ***(Sakit iti ulo)***<br>14. Cause irritability ***(Baka aggagatel)***<br>15. Increase libido/turn into a maniac ***(Baka mangpaado iti libido wenno agbalin ka nga manyakis)***<br>16. Cause loss/reduce of libido ***(Mangpaadu wenno mangpabassit iti libido)***<br>17. Cause loss/reduce of sexual satisfaction ***(Baka mangpabassit iti sexual satisfaction)***<br>18. One will not have children anymore ***(Baka haan kan to nga agkaanaken)***<br>19. Not fully effective, woman could still get pregnant ***(Haan unay nga epektibo, agsikog latta jay babae)***<br>20. When it does not work, the baby is born with abnormalities ***(Baka maiyanak nga ada abnormalidad jay anak na)***<br>21. Results in mortal sin because it is against church teachings ***(Detoy ket mesa nga basol. Haan nga kayat isi simbaan iti kastoy)***<br><br>**IUD/Implants**<br>22. Melt or move around inside the body and doctors will not be able to find ***(Baka sumrek iti bagi***							

	ken haan nga mabirukan iti doktor)<br>23. Washed away/pushed out of body ***(Baka iruwar iti bagi)***<br>24. Painful to insert ***(Nasakit nga isirrek)***<br><br>**IUD**<br>25. Itchy on the vagina ***(Panaggagatel iti oki)***<br>26. Entangled around the man's penis ***(Baka sumab-it iti boto ti lalaki)***<br>27. Messy when inserted ***(Nagulo nu isirrek)***<br><br>**Male sterilization**<br>28. Part of the man's testicles are cut off<br>29. It hurts the testicles ***(Nasakit)***<br>30. The man loses his manhood ("kapon")<br><br>31. Others (specify)							
303	About which family planning methods do you have concerns?<br><br>***Anya dagiay family planning methods iti kayat mo nga damagen ken mamwan pay?***<br><br>REPEAT EACH CONCERN IN TURN. FOR EACH CONCERN, WRITE DOWN ALL METHODS CAUSING THAT CONCERN.<br><br>1. Female sterilization<br>2. Male sterilization<br>3. IUD<br>4. Injectable<br>5. Implants<br>6. Patch<br>7. Pill<br>8. Other modern method (specify)							-> 304

	9. Other method (specify)							
304	Who told you or how did you find about your concerns about family planning methods?<br><br>***Sino iti nangibaga wenno kasanom nga naamwan dagiti kayat mo sa maamwan gapu iti family planning methods?***<br><br>REPEAT EACH CONCERN IN TURN. FOR EACH WRITE DOWN ALL SOUCES OF INFORMATION.<br><br>1. Health staff<br>2. BHW or health volunteers<br>3. Husband or partner<br>4. Friend, neighbours, relatives<br>5. Church<br>6. Radio<br>7. Television<br>8. Newspaper or magazine<br>9. Online or internet<br>10. Others (specify)							-> 305
305	Today, did any staff member at the health facility speak to you about family planning methods?<br><br>***Adda kadi staff member dijay health facility na nagsarita kanyam tatta gapu iti family planning methods?***	1. Yes<br>2. No<br><br>***1. Wen***<br>*2.* ***Haan***						1 ->306<br>2 ->401
306	Did the health worker ask you about your concerns?<br><br>***Dinamag da ka kadi dagiti health workers gapu dagiti kayat mo nga maamwan?***	1. Yes<br>2. No<br><br>***3. Wen***<br>***Haan***						1 ->307<br>2 -> 309
307	Do you feel the health worker understands your concerns?<br><br>***Naawatan na kadi dejay health worker dagijay kayat mo nga damagen wenno amwen?***	1. Yes<br>2. No<br><br>***1. Wen***<br>2. ***Haan***						->308

308	Did the health worker help you to find solutions to your concerns?<br><br>***Tinulungan na ka kadi dejay health worker nga biruken iti sungbat kadagijay damdamag mo?***	1. Yes<br>2. No<br><br>***1. Wen***<br>2. ***Haan***		->309
309	Did the health worker offer you information how different family planning methods work?<br><br>***Inbaga na kadi dejay health worker nu kasano nga usaren dagijay family planning methods?***	1. Yes<br>2. No<br><br>***1. Wen***<br>*2.* ***Haan***		1 ->310<br>2 ->312
310	Which methods did health worker mention today?<br><br>***Anya nga methods wenno anya nga klase iti family planning iti inbaga na dejay health worker?***	1. Female sterilization<br>2. Male sterilization<br>3. IUD<br>4. Injectable (e.g.DMPA)<br>5. Implants<br>6. Patch<br>7. Pill<br>8. Condom<br>9. Female condom<br>10. Diaphragm<br>11. Form/Jelly/Cream<br>12. Mucus/Billings/Ovulation<br>13. Basal body temperature<br>14. Symptothermal<br>15. Standard days method<br>16. LAM<br>17. Calendar/Rhythm/Periodic abstinence<br>18. Withdrawal<br>19. Other traditional method<br>20. Other modern method (specify)		- >311
311	Did the health worker tell you about side-effects or problems you might have with any methods of family planning?	1. Yes<br>2. No<br><br>***1. Wen***<br>*2.* ***Haan***		-> 312

	Inbaga ma kadi dejay health worker nga mabalin nga adda side effect wenno agbalin nga agkaproblema ka gapu iti panag-usar mo iti family planning methods?			
312	Did the health worker offer you information how your family planning method works?<br><br>***Inbaga na kadi dejay health worker nu kasano nga usaren dagijay family planning methods?***	1. Yes<br>2. No<br>3. N/A (not using a method now)<br><br>***1. Wen***<br>***2. Haan***<br>***3. Haan nga agus-usar iti uray anya***		1 -> 313<br>2-> 313<br>3-> 315
313	Did the health worker explain about the side effects of your current method?<br><br>***In-explikar na kadi dejay health worker iti side effects na jay us-usarem tatta nga klase iti family planning method?***	1. Yes<br>2. No<br><br>***1. Wen***<br>*2.* ***Haan***		-> 314
314	Did the health worker ask you to describe how you use your current method?<br><br>***Dinamag na ka ba dejay health worker nga nu kasanom nga us-usaren dagiti family planning method?***	1. Yes<br>2. No<br><br>***1. Wen***<br>2. ***Haan***		-> 401
315	After receiving FP counselling will you begin using a family planning method today?<br><br>***Nirugyam kadi iti agusar iti family planning method tatta nga nalpas iti Family Planning counseling?***	1. Yes<br>2. No<br><br>***1. Wen***<br>2. ***Haan***		1 -> 317<br>2 -> 316
316	After receiving FP counselling will you begin using, do you think you will use a	1. Yes<br>2. No<br><br>***1. Wen***		1-> 317<br>2-> 401

	contraceptive method anytime in the future?<br><br>***Tatta nga nalpas iti family planning counseling, rugyam kadi iti agusar iti contraceptive method wenno agusar kan to pay lang?***	2. ***Haan***		
317	Which contraceptive method would you prefer to use?<br><br>***Anya nga contraceptive method iti kayat mo nga usaren?***	1. Female sterilization<br>2. Male sterilization<br>3. IUD<br>4. Injectable (e.g.DMPA)<br>5. Implants<br>6. Patch<br>7. Pill<br>8. Condom<br>9. Female condom<br>10. Diaphragm<br>11. Form/Jelly/Cream<br>12. Mucus/Billings/Ovulation<br>13. Basal body temperature<br>14. Symptothermal<br>15. Standard days method<br>16. LAM<br>17. Calendar/Rhythm/Periodic abstinence<br>18. Withdrawal<br>19. Other traditional method<br>20. Other modern method (specify)		-> 401

	Section 4. Past Health facility visit and FP counseling<br>**Do not count today's visit.**							
401	Not including today, in the last 12 months, have you visited a health facility for care for yourself or your children for any purpose?<br><br>***Idi napalabas nga 12 nga bulan, napan ka kadi nagpasyar iti health facility tapno agpa check-up para ti sarilim wenno para iti anak mo?***	1. Yes<br>2. No<br><br>***1. Wen***<br>2. ***Haan***						1 -> 402<br>2 -> End of interview
	LINE NUMBER	01	02	03	04	05	06	
402	Now I would like to record all your facility visits for last 12 months. Start with the latest visit you had.<br>Why did you visit a health facility?<br><br>***Kayat ko i-record amin nga panagpasyar mo iti health facility idi napalabas ng 12 nga bulan . Rugyan ta idi naudi na papan mo iti health facility.***<br><br>***Apay nga napan ka iti mesa nga health facility?***<br><br>AFTER WRITING THE FIRST VISIT IN LINE NUMBER 01, ASK Q403-410 FOR THAT VISIT. THEN ASK THE 2nd LATEST VISIT TO WRITE IN 402 LINE NUMBER 02, THEN ASK Q 403 AND Q404.<br>REPEAT FOR ALL HEALTH FACILITY VISITS FOR LAST 12 MONTHS.<br>IF THERE ARE MORE THAN 6, USE AN ADDITIONAL QUESTIONNAIRE.<br><br>1. Prenatal care ***(Check-up sakbay nga aganak)***<br>2. Giving birth, while a women is still in the facility							-> 403

	(Manganak pay lang jay babae ken adidjay pa lang health facility)<br>3. Health check after giving birth, after a woman left the facility ***(Check-up kalpasan nga nagannak jay babe ngem awan suna jay health facility)***<br>4. Receiving vaccination or routine check up for child ***(Makaala iti bakuna para ti babassit pay nga anak)***<br>5. Seeking medical advice or treatment for sickness or injury of **child** ***(Agpaagas wenno agpakita iti doctor para ti masaksakit wenno nadunor nga anak)***<br>6. Seeking medical advice or treatment for sickness or injury of **herself** ***(Agpaagas wenno agpakita iti doktor para ti bagbagi)***<br>7. Adolescent clinic<br>8. Other (specify) ***Sabali pay***							
403	Where did you visit?<br><br>***Napanam nga health facility?***<br><br>1. National hospital<br>2. Regional hospital/Public medical center<br>3. Provincial hospital<br>4. District hospital<br>5. Municipal hospital<br>6. Rural health unit (RHU)/urban health center(UHC)/Lying-in<br>7. Barangay health station (BHS)<br>8. Barangay supply/service point officer/BHW<br>9. Mobile clinic<br>10. Other (specify. Private facility is included here.)							-> 404

404	At that visit, were you or your sexual partner already using any method to delay or avoid getting pregnant?<br><br>***Idi napan ka bimmisita iti mesa nga health facility, ag-us-usar ka kadin iti contraceptive method tapno haan ka nga agsikog insigida?***<br><br>1. Yes<br>2. No<br><br>***1. Wen***<br>***2. Haan***							1 ->405<br><br>2 -> 406
405	Which method(s) were you using?<br><br>***Anya dagiti methods iti us-usarem wenno ub-ubraem?***<br><br>WRITE DOWN ALL MENTIONED<br><br>1. Female sterilization<br>2. Male sterilization<br>3. IUD<br>4. Injectable (e.g.DMPA)<br>5. Implants<br>6. Patch<br>7. Pill<br>8. Condom<br>9. Female condom<br>10. Diaphragm<br>11. Form/Jelly/Cream<br>12. Mucus/Billings/Ovulation<br>13. Basal body temperature<br>14. Symptothermal<br>15. Standard days method<br>16. LAM<br>17. Calendar/Rhythm/Periodic abstinence<br>18. Withdrawal<br>19. Other traditional method<br>20. Other modern method (specify)							->406

406	At that visit, did any staff member at the health facility speak to you about family planning methods?<br><br>***Idi napan ka bimmisita, adda kadi health staff member dijay health facility nga nangsarita kanyam gapu iti family planning methods?***<br><br>1. Yes<br>2. No<br><br>***1. Wen***<br>*2.* ***Haan***							1-> 407<br>2-> 402 next line number
407	After that visit, did you start using any FP method or change from your previous method to a new method?<br><br>***Idi nalpas iti visit, nangrugi ka kadin nga nangusar iti Family Planning method wenno nagsabali ka iti method nga inusar?***<br>1. Yes<br>2. No<br><br>***1. Wen***<br>*2.* ***Haan***							1 -> 409<br>2 ->408
408	If you did not start a new method or change from your previous method, why?<br><br>***Apay nga dim sinuktan iti method nga us-usarem manipud iti dati?***<br><br>**1.** No need **(*Haan nga masapol)***<br>2. Possible side effects of new method **(*Baka adda iti side effects na)***<br>3. New method not available at the facility ***(Dijay method ket awan dijay facility)***							

	4. Concerns about risk of pregnancy with new method ***(Adu ti damdamag ko gapu iti kabarbaro nga method)***<br><br>***5.*** Not enough information ***(Awan unay iti ammo da)***<br><br>6. Could not afford to purchase ***(Haan ko kaya nga gumatang)***<br><br>7. Advice of friends, relatives, neighbours not to start or change ***(Isu iti kuna dagijay gagayem, haan kano pay rugyan wenno haan nga suktan)***<br><br>8. Husband/partner did not support ***(Haan nga sinuportaran jay asawa)***<br><br>9. Other (specify):__________							
409	Which FP method did you start using after that visit or which new method did you change to?<br><br>***Anya nga family planning method iti nirugyam nga usaren kalpasan nga napan ka dijay health facility?***<br><br>***Anya nga method iti sinuktam nga usaren?***<br><br>1. Female sterilization<br>2. Male sterilization<br>3. IUD<br>4. Injectable (e.g.DMPA)<br>5. Implants<br>6. Patch<br>7. Pill<br>8. Condom<br>9. Female condom							-> 402 next line number

	10. Diaphragm<br>11. Form/Jelly/Cream<br>12. Mucus/Billings/Ovulation<br>13. Basal body temperature<br>14. Symptothermal<br>15. Standard days method<br>16. LAM<br>17. Calendar/Rhythm/Periodic abstinence<br>18. Withdrawal<br>19. Other traditional method<br>20. Other modern method (specify)							

END OF THE INTERVIEW
