# Supplementary material for: Opportunities lost: Barriers to increasing the use of effective contraception in the Philippines
Source: PLoS One. 2019 Jul 25;14(7):e0218187. doi: 10.1371/journal.pone.0218187 (PMC6657820; doi:10.1371/journal.pone.0218187)
Supplement: S5 Questionnaire — (PDF) [file pone.0218187.s005.pdf]

COVER PAGE

FORM1. Iinterview women of reproductive age who are not currently pregnant or within 6 weeks of delivery, and desire delaying or limiting childbearing

Sequence number: _____________

[Fill one number for each woman contacted in the order they were contacted at the health facility; if done over several days, continue unique sequence numbers]

Identification of interview place	
Region /*Rehiyon*	
Province /*Probinsya*	
CITY/MUNICIPALITY /*SIYUDAD/MUNISIPALIDAD*	
BARANGAY /*Barrio*	
Health facility name/ *Ngalan sang Health Facility*	
Home address (for home visit only)	
Latitude and longitude<br>(Use the coordinate of GPS in a mobile phone)	
**Interview Record**	
Date of interview /*Petsa kang Interbyu*	
Interviewer's name /*Ngalan sang naga-interbyu*	
Health facility level where interview took place<br><br>*Health facility kung diin ginhiwat ang pag interbyu?*	1. National hospital /National nga Ospital<br>2. Regional hospital/Public medical center<br>3. Provincial hospital/Probinsyal nga Ospital<br>4. District hospital/District Ospital<br>5. Municipal hospital/ Munisipal Ospital<br>6. Rural health unit (RHU)/urban health center(UHC)/Lying-in<br>7. Barangay health station (BHS)<br>8. Barangay supply/service point officer/BHW<br>9. Mobile clinic<br>10.Other (specify)
Clinic where interview took place (for large hospitals)<br><br>*Klinika kung diin ginhiwat ang pag-interbyu(Para sa dalagku nga Ospital)*	1. Postnatal health check after giving birth, after a woman left the facility<br>2. Receiving vaccination or routine check-up for child<br>3. Seeking medical advice or treatment for sickness or injury of child<br>4. Seeking medical advice or treatment for sickness or injury of **herself**<br>5. Adolescent clinic<br>6. Other (specify)

Sequence Number: _________________
[Write the same sequence number from Cover Page]

Instructions:
Read the information sheet. Answer questions. If the woman agrees to participate give the certificate of consent for her to sign. Then start the Screening Form.

Basaha ang Information Sheet. Sabton ang mga pamangakit
Screening Form

State: "We would like to start by asking a few questions that determine if you are eligible for the survey."

001	How old were you on your last birthday?<br>*Pila ang imo edad sa ulihi mo nga kaadlawan?*	Age in completed years<br><br>*Edad sa natapos nga tinuig*		15-49 years ->002<br>Other -> 009
002	Are you pregnant now?<br>*Ikaw bala nagabusong subong?*	1. Yes - *Hou*<br>2. No - *Hindi*<br>3. Unsure- *Naga duha-duha*		1 ->009<br>2 ->003<br>3 ->003
003	What is the name of your last baby?<br><br>*Ano ang ngalan sang agot mo nga bata?*<br><br>Record name	1. Name<br>*Ngalan*____________<br>2. No previous baby<br>*Wala pa sang bata*		1 ->004<br>2 ->006
004	In what month and year was NAME born?<br><br>*Sa ano nga bulan kag tuig si _____ nabata*<br>(probe: when is his or her birthday<br>*Sang san-o iya nga kaadlawan*)	Month/*Bulan*: __ __<br><br>Year/ *Tuig*: __ __ __ __		Age ≥6 wks ->005<br>Age<6 wks ->009<br><br>Edad ≥ 6 ka semana ->005<br><br>Edad < 6 ka semana ->005
005	Has your menstrual period returned since the birth of NAME?<br>*Nakabalik na bala ang imo regla pagkatapos sang pagbata mo kay ________*	1. Yes/*Hou*<br>2. No/*Hindi*		1 ->006<br>2 ->006
006	Now I have some questions about the future. Would you like to have (a/another) child, or would you prefer not to have any (more) children?	1. Have (a/another) child<br>*Magpadugang sang bata*<br>2. No more/none<br>*Wala na/Hindi na*<br>3. Cannot get pregnant		1 -> 007<br>2 ->008<br>3 ->009<br>4 ->009

	May ara ako pamangkot na buwasdamlag, Gusto mo pa bala sang dugang nga bata ukon hindi mo na gusto?	*Hindi maka busong*<br>4. Undecided / don't know<br>*Wala kabalu/Duhaduha*		
007	Do you want (a/another) child soon?<br><br>*Gusto mo bala bala sang dugang nga bata?*	1. Yes- *Hou*<br>2. No, want to wait<br>*Hindi, Gusto maghulat*<br>3. Don't know<br>*Wala kabalu*		1 ->009<br>2 ->008<br>3- >009
008	Are you or your husband/partner currently doing something or using any method to delay or avoid getting pregnant?<br><br>*Ikaw bala sang imo bana/kaupod subong naga-obra sang isa ka bagay ukon naga gamit sang paagi para atrasuhon or malikaw ang pagbusong?*	1. Yes -*Hou*<br>2. No -*Hindi*		1 -> 101<br>2 -> 101<br>To achieve a total of 5 users and non-users (hospitals) and 3 users and 3 non-users (health centres).
009	Thank the woman, indicate ineligibility for the survey and stop the interview. Enter this woman into "number of women contacted". Then find another woman to interview.			

QUESTIONNAIRE

FORM1. Interview of women of reproductive age who are not currently pregnant or within 6 weeks of delivery, and desire delaying or limiting childbearing

Sequence Number:
[Write the same sequence number from Cover Page]

NO.	Section 1. Respondent background		
101	In (month of interview) 2016, did you live in a city, in a town proper/ poblacion, in the barrio or rural area, or abroad?<br><br>*In (Bulan sang Interbyu), 2016 ikaw bala nakatinir sa siyudad, sa banwa/poblacion, sa barrio ukon sa rural nga lugar, ukon sa iban nga pungsod?*	1. City<br>2. TOWN PROPERIPOBLACION<br>3. BARRIO/RURAL AREA<br>4. ABROAD<br>5. DON'T KNOW	->102
102	What is your marital status now?<br><br>*Ano ang imo estado subong?*	1. Never married or never lived with a man<br>2. Currently married<br>3. Currently living with a man<br>4. Divorced/separated/widow and not currently living with a man	->103
103	What is your highest level of education attended, whether or not that level was completed?<br><br>*Ano ang imo pinakataas nga edukasyon nga natapos, ano ang imo na kompleto?*	1. No education<br>2. Elementary<br>3. High school<br>4. College<br>5. Post-graduate	->104
104	How many children do you have who are still alive?<br><br>*Pila ang imo bata nga buhi pa asta subong?*	Number of children alive<br><br>*Numero sang kabataan nga buhi?*	->106
105	Did you or someone else do anything to end any of your past pregnancies?<br><br>*Ikaw bala ukon isa ka tawo nagaobra sang is aka bagay para matapos ang nagligad mo nga pagbusong?*	1. Yes<br>2. No	1 ->107<br>2 -> 108

106	How many pregnancies did you or someone else do anything to end?<br><br>Pila ka pagbusong ang imo ukon isa ka tawo naghimo sang paagi nga matapos?	Number of induced abortion	->108
107	Are you covered by any health insurance, either as member or dependent?<br><br>*Ikaw bala nasakop sang is aka "health insurance" bilang miymbero ukon sinaguran?*	1. Not covered<br>2. Philhealth<br>3. Government Service Insurance System<br>4. Social Security System<br>5. Private insurance company/Health maintenance organization /Pre-need insurance plan company<br>6. Other (Specify)	->201

NO.	Section 2. Current use of FP			
201	REVIEW: Are you or your husband/partner currently doing something or using any method to delay or avoid getting pregnant?<br><br>*Ikaw bala sang imo bana/kaupod subong naga-obra sang paagi para mapa ulihi ukon malikawan ang magbusong?*	1. Yes<br>2. No		1 -> 202<br>2 -> 206
202	Which method are you currently using?<br><br>*Ano nga paagi subong ang gina-gamit mo?*<br><br>WRITE DOWN ALL MENTIONED.	1. Female sterilization<br>2. Male sterilization<br>3. IUD<br>4. Injectable (e.g.DMPA)<br>5. Implants<br>6. Patch<br>7. Pill<br>8. Condom<br>9. Female condom<br>10. Diaphragm<br>11. Form/Jelly/Cream<br>12. Mucus/Billings/Ovulation<br>13. Basal body temperature<br>14. Symptothermal<br>15. Standard days method<br>16. LAM<br>17. Calendar/Rhythm/Periodic abstinence<br>18. Withdrawal		-> 203

		19. Other traditional method<br>20. Other modern method (specify)					
	LINE NUMBER	01	02	03	04	05	
203	Now I would like to ask you one by one about all methods you are using now.<br><br>*Subong, ako mamangkot sa imo isa-isa sang mga paagi ng imo gingamit.*<br><br>RECORD ALL METHODS BEING USED NOW, ONE METHOD PER ONE LINE NUMBER.<br>IF THERE ARE MORE THAN 5 METHODS, USE ADDITIONAL QUESTIONNAIRE.<br><br>*Isulat ang paagi nga ginagamit sa isa ka numero. Kung sobra sa lim (5) ka maagi, mag gamit sang dugang nga "questionnaire"*<br><br>1. Female sterilization<br>2. Male sterilization<br>3. IUD<br>4. Injectable (e.g.DMPA)<br>5. Implants<br>6. Patch<br>7. Pill<br>8. Condom<br>9. Female condom<br>10. Diaphragm<br>11. Form/Jelly/Cream<br>12. Mucus/Billings/Ovulation<br>13. Basal body temperature<br>14. Symptothermal<br>15. Standard days method<br>16. LAM<br>17. Calendar/Rhythm/Periodic abstinence<br>18. Withdrawal<br>19. Other traditional method<br>20. Other modern method (specify)						-> 204
204	Where did you obtain that method when you first started using it?						-> 205

	Sa diin mo nakuha ang paagi sang una mo nga gamit sini?<br><br>1. National hospital<br>2. Regional hospital/Public medical center<br>3. Provincial hospital<br>4. District hospital<br>5. Municipal hospital<br>6. Rural health unit (RHU)/urban health center(UHC)/Lying-in<br>7. Barangay health station (BHS)<br>8. Barangay supply/service point officer/BHW<br>9. Mobile clinic<br>10. Other (specify. Private facility is included here)						
205	What was the purpose of your going to the health facility on the day you first received the contraceptive method?<br><br>*Ano ang imo katuyoan sang imo pagkadto sa health facility sa adlw nga imo nabaton ang paagi sang pangontra sa pagbusong?*<br><br>1. Prenatal care<br>2. Giving birth, while a women is still in the facility<br>3. Health check after giving birth, after a woman left the facility<br>4. Receiving vaccination or routine check up for child<br>5. Seeking medical advice or treatment for sickness or injury of **child**<br>6. Seeking medical advice or treatment for sickness or injury of **herself**<br>7. Adolescent clinic<br>8. Other (specify)						-> 203. Repeat until all methods were explained.<br><br>Then ->206
206	If you <u>are not</u> using any method to delay or avoid getting pregnant now, have you or your sexual partner done		1.<br>2.	Yes<br>No			1->207<br>2-> 301

	something or used a method to delay or avoid getting pregnant in the past?<br><br>*Kung ikaw wala naga-gamit sang pagi para mapa atras ukon malikawan ng pagbusong subong, ikaw bala sang imo kalaguyo nakaobra kon para mapaatras ukon malikawan ang pagbusong sang una?*<br><br>If you are using a method to delay or avoid getting pregnant now, have you or your sexual partner ever used a different method to delay or avoid getting pregnant in the past?<br><br>*Kung ikaw _naga-gamit sang pagi para mapa atras ukon malikawan ng pagbusong subong, ikaw bala sang imo kalaguyo nakaobra kon para mapaatras ukon malikawan ang pagbusong sang nagligad?*						
207	Which methods have you used in the past?<br><br>Ano nga paagi ang imo nagamit sang nagligad?<br><br>WRITE DOWN ALL MENTIONED.<br><br>*Isulat ang mga nalista:*	1. Female sterilization<br>2. Male sterilization<br>3. IUD<br>4. Injectable (e.g.DMPA)<br>5. Implants<br>6. Patch<br>7. Pill<br>8. Condom<br>9. Female condom<br>10. Diaphragm<br>11. Form/Jelly/Cream<br>12. Mucus/Billings/Ovulation<br>13. Basal body temperature<br>14. Symptothermal<br>15. Standard days method<br>16. LAM<br>17. Calendar/Rhythm/Periodi c abstinence<br>18. Withdrawal<br>19. Other traditional method<br>20. Other modern method (specify)					-> 208
	LINE NUMBER	01	02	03	04	05	

208	Now I would like to ask you one by one about all methods you have used in the past<br><br>*Subong, ako mamangkot isa-isa sang tanan nga paagi nga imo gingamit sang nagligad*<br><br>RECORD ALL METHODS, ONE METHOD PER ONE LINE NUMBER.<br>IF THERE ARE MORE THAN 5 METHODS, USE ADDITIONAL QUESTIONNAIRE.<br><br>Isulat ang tanan nga paagi, isa ka paagi sa isa ka linya sang numero Kung sobra sa lima (5) mag gamit sang dugang nga questionnaire.<br><br>1. Female sterilization<br>2. Male sterilization<br>3. IUD<br>4. Injectable (e.g.DMPA)<br>5. Implants<br>6. Patch<br>7. Pill<br>8. Condom<br>9. Female condom<br>10. Diaphragm<br>11. Form/Jelly/Cream<br>12. Mucus/Billings/Ovulation<br>13. Basal body temperature<br>14. Symptothermal<br>15. Standard days method<br>16. LAM<br>17. Calendar/Rhythm/Periodic abstinence<br>18. Withdrawal<br>19. Other traditional method<br>20. Other modern method (specify)						-> 209
209	Where did you obtain the family planning method when you first started using it?<br><br>*Diin ikaw nakakuha sang paagi sang pagplano sang pamilya kang una mo ini nga gingamit?*						-> 210

	1. National hospital<br>2. Regional hospital/Public medical center<br>3. Provincial hospital<br>4. District hospital<br>5. Municipal hospital<br>6. Rural health unit (RHU)/urban health center(UHC)/Lying-in<br>7. Barangay health station (BHS)<br>8. Barangay supply/service point officer/BHW<br>9. Mobile clinic<br>10.Other (specify. Private facility is included here.)						
210	Why did you visit the health facility where you first started using the family planning method?<br><br>*Ngaa ikaw nag bisita sang "health facility sang una mo gingamit ang mga pamaagi sa pagplano sang pamilya?*<br><br>1. Prenatal care<br>2. Giving birth, while still in the facility<br>3. Health check after giving birth, after leaving the facility<br>4. Receiving vaccinations or routine check-ups for a child<br>5. Seeking medical advice or treatment for sickness or injury of a **child**<br>6. Seeking medical advice or treatment for sickness or injury of **herself**<br>7. Adolescent clinic<br>8. Other (specify)						-> 211
211	Why did you stop using the family planning method that you used in the past?<br><br>*Ngaa imo gin –tapna(untat) ang paggamit sang pagplano sang*						-> 212

	pamilya nga gingamit mo sang nagligad?<br><br>1. Side effects<br>*Epekto*<br>2. Method not available at the facility<br>Hindi makuha sa Health Facility<br>3. Concerns about risks of pregnancy<br>*Naga-ulikid sang risgo sang pagbusong*<br>4. Could not afford to purchase<br>*Hindi makasarang magbakal*<br>*5.* Health worker did not continue to provide the method<br>*Wala ginpadayon ang paghatag sang health worker sang pamaagi?*<br>6. Advice of friends, relatives, neighbors<br>*Laygay sang mga abyan, pamilya, tupad-balay*<br>7. Husband/partner did not support<br>*Bana/Kaupiod wala naga suporta*<br>8. Wanted to get pregnant<br>*Gusto magbusong*<br>9. Other<br>*Iban pa*<br>(specify):__________						

	**Section 3. FP Concerns and Today's FP counseling**							
301	Do you have any health concerns about any type of family planning method?<br>*My ara ka bala pag-ulikid sa ikaayong lawas tungod sa pamaagi sang pagplano sang pamilya?*	1. Yes<br>2. No						1 ->302<br>2 ->305
	LINE NUMBER	01	02	03	04	05	06	
302	What are your health concerns about family planning methods?<br>Please tell me one by one.<br><br>*Ano ang imo mga pag-ulikid sa ikaayong lawas tungod s pamaagi sang pagplano sang pamilya?*<br><br>USE ONE LINE NUMBER FOR ONE CONCERN. WRITE DOWN ALL MENTIONED CONCERNS.<br>IF THERE ARE MORE THAN 6 CONCERNS, USE ADDITIONAL QUESTIONNAIRE.<br><br>1. Cause cancer in the uterus<br>*Kabangdanan sang Cancer sa Uterus*<br><br>2. Cause cysts in the uterus<br>*Kabangdanan sng cysts sa Uterus*<br><br>3. Cause infection of the uterus<br>*Kabangdanan sang inpeksyun sa uterus*<br><br>4. Cause frequent bleeding<br>*Kabangdanan sang sige nga pagdurugo*<br><br>5. Cause thyroid problems<br>*Kabangdanan sang mga Problema sa Uterus*<br>6. Cause/worse asthma<br>*Kabangdaan/Makapalala sang Asthma*							-> 303

	7. Cause/worse lots of veins<br>*Kabangdanan sang pagdamo sang ugat*<br><br>8. Cause dry skin, skin disease<br>*Kabangdanan sng pag mala sang panit, sakit sa panit*<br><br>9. Cause edema<br>*Kabandanan sng Edema*<br><br>10. Cause weight gain<br>*Kabangdanan sang pagbug-at sang timbang*<br><br>11. Cause weight loss<br>*Kabangdanan sang Pagbuhin sang timbang*<br><br>12. Cause bloated stomach<br>*Kabangdann sang pagdaku sang tiyan*<br><br>13. Cause headache<br>*Kabangdanan sang sakit sang ulo*<br><br>14. Cause irritability<br>*Kabangdanan sang Pagka-Iritable*<br><br>15. Increase libido/turn into a maniac<br>*Pagdugang sang libido/ naga maniac*<br><br>16. Cause loss/reduce of libido<br>*Kabandanan sangpagdula/pagbuhin sang libido*<br><br>17. Cause loss/reduce of sexual satisfaction<br>*Kabangdanan sang pagdula/pagbuhin sang kaayawan sa pakipaghilawas*							

	18. One will not have children anymore<br>*Indi na makapadugang sang kabataan*<br><br>19. Not fully effective, woman could still get pregnant<br>*Hindi epektibo, ang babaye makabusong pa liwat*<br><br>20. When it does not work, the baby is born with abnormalities<br>*Kung hindi mag work, ang bata ma guwa nga may abnormalidad.*<br><br>21. Results in mortal sin because it is against church teachings<br><br>*Resulta sa mortal nga sala tugod batok sa gnatudlo sang simbahan*<br><br>**IUD/Implants**<br>22. Melt or move around inside the body and doctors will not be able to find<br>*Nagatunaw ukon nagahulag sa sulod sang lawas kag ang mga doctor indi makakita.*<br><br>23. Washed away/pushed out of body<br>Ma dula ukon matulod paguwa sa lawas.<br><br>24. Painful to insert<br>*Masaki tang pagsulod*<br><br>**IUD**<br>25. Itchy on the vagina<br>Makatol sa kinatawo sang babaye.<br><br>26. Entangled around the man’s penis<br>“ Mag lubid sa kinatawo sang lalaki.							

	27. Messy when inserted<br>*Kagamo ang pagpasulod*<br><br>**Male sterilization**<br>28. Part of the man's testicles are cut off<br>*Ang parti sang testicle ginautod*<br><br>29. It hurts the testicles<br>*Masakit sa testicle*<br><br>*30.* The man loses his manhood<br>*Nagakadula ang pagka lalaki* ("kapon")<br><br>31. Others (specify)							
303	About which family planning methods do you have concerns?<br><br>*Ano ang pamaagi sang pagplano sang pamilya ikaw may pag-ulikid?*<br><br>REPEAT EACH CONCERN IN TURN. FOR EACH CONCERN, WRITE DOWN ALL METHODS CAUSING THAT CONCERN.<br><br>1. Female sterilization<br>2. Male sterilization<br>3. IUD<br>4. Injectable<br>5. Implants<br>6. Patch<br>7. Pill<br>8. Other modern method (specify)<br>9. Other method (specify)							-> 304
304	Who told you or how did you find about your concerns about family planning methods?<br><br>*Sino nagsugid sa imo ukon paano mo nabal-an ang tungod sa paagi sa pagplano sang pamilya?*							-> 305

	REPEAT EACH CONCERN IN TURN.<br>FOR EACH WRITE DOWN ALL SOUCES OF INFORMATION.<br><br>*Liwaton ang isa ka concern. Isulat ang ginhalinan sang Impormasyon.*<br><br>1. Health staff<br>2. BHW or health volunteers<br>3. Husband or partner<br>4. Friend, neighbours, relatives<br>5. Church<br>6. Radio<br>7. Television<br>8. Newspaper or magazine<br>9. Online or internet<br>10. Others (specify)							
305	Today, did any staff member at the health facility speak to you about family planning methods?<br><br>*Subong nga adlaw, may ara bala sang Health Center nga naghambal sa imo tungod sa mga paagi sa pagplano sang pamilya?*	1. Yes *(Hou)*<br>2. No (Hindi)						1 ->306<br>2 ->401
306	Did the health worker ask you about your concerns?<br>*Ang Health worker bala nagpamangkot sang imo*	1. Yes *(Hou)*<br>2. No (Hindi)						1 ->306<br>2 ->309
307	Do you feel the health worker understands your concerns?<br><br>*Naintindihan bala sang health worker ang imo gna-ulikdan?*	1. Yes<br>2. No						->308
308	Did the health worker help you to find solutions to your concerns?<br><br>*Ang health worker bla nakabulig sang pangita sulosyon sa imo gna- ulikdan?*	1. Yes<br>2. No						->308
309	Did the health worker offer you information how different family planning methods work?	1. Yes<br>2. No						1 ->310<br>2 ->312

	Ang health worker bala nag hatag sa imo sang impormasyon paano naga obra ang iba iba nga pamaagi sang pagplano sang pamilya?			
310	Which methods did health worker mention today?<br><br>*Ano nga pamaagi ang na hambal sang health worker sa imo?*	1. Female sterilization<br>2. Male sterilization<br>3. IUD<br>4. Injectable (e.g.DMPA)<br>5. Implants<br>6. Patch<br>7. Pill<br>8. Condom<br>9. Female condom<br>10. Diaphragm<br>11. Form/Jelly/Cream<br>12. Mucus/Billings/Ovulation<br>13. Basal body temperature<br>14. Symptothermal<br>15. Standard days method<br>16. LAM<br>17. Calendar/Rhythm/Periodic abstinence<br>18. Withdrawal<br>19. Other traditional method<br>20. Other modern method (specify)		- >311
311	Did the health worker tell you about side-effects or problems you might have with any methods of family planning?<br><br>*Ang health worker bala naghambal sa imo sang epekto ukon maging problema sa paggamit sang paagi sang pagplano sang pamilya?*	1. Yes<br>2. No		- >312
312	Did the health worker offer you information how your family planning method works*?*<br>*Ang health worker bala naghatag sa imo sang impormasyon paano mag obra ang mga pamaagi sang pagplano sang pamilya?*	1. Yes<br>2. No<br>3. N/A (not using a method now)		1 -> 313<br>2-> 313<br>3-> 315
313	Did the health worker explain about the side effects of your current method?	1. Yes<br>2. No		-> 314

	Ang health worker bala nag eksplikar sang mga epekto sang gnagamit mo nga pamaagi subong?			
314	Did the health worker ask you to describe how you use your current method?<br><br>*Ang health worker bala namangkot sa imo kung paano mo ginagamit ang pamaagi?*	1. Yes<br>2. No		> 401
315	After receiving FP counselling will you begin using a family planning method today?<br><br>*Pagkatapos bala sang Family Planning n counseling, ikaw bala maga umpisa gamit sang pamaagi sang pagplano sang pamilya subong?*	1. Yes<br>2. No		1 -> 317<br>2 -> 316
316	After receiving FP counselling will you begin using, do you think you will use a contraceptive method anytime in the future?<br>*Pagkatapos mo mabaton ang FP Counselling, ikaw bala maga umpisa gamit subong ukon ikaw magagamit sang contraceptive sa sunod nga mga tiyempo?*	1. Yes<br>2. No		1 -> 317<br>2 -> 401
317	Which contraceptive method would you prefer to use?<br><br>*Ano nga contraceptive ang imo nga pillion?*	1. Female sterilization<br>2. Male sterilization<br>3. IUD<br>4. Injectable (e.g.DMPA)<br>5. Implants<br>6. Patch<br>7. Pill<br>8. Condom<br>9. Female condom<br>10. Diaphragm<br>11. Form/Jelly/Cream<br>12. Mucus/Billings/Ovulation<br>13. Basal body temperature<br>14. Symptothermal<br>15. Standard days method<br>16. LAM<br>17. Calendar/Rhythm/Periodic abstinence		-> 401

		18. Withdrawal<br>19. Other traditional method<br>20. Other modern method (specify)		

	Section 4. Past Health facility visit and FP counseling<br>Mga nagligagad nga pagbisita sa Health Facility ukon FP Counseling<br>**Do not count today's visit.**							
401	Not including today, in the last 12 months, have you visited a health facility for care for yourself or your children for any purpose?<br><br>*Wala naupod subong nga adlaw, nakabisita ka bala sa health facility ukon naga ulikid ka bala sa imo kaugalingon ukon imo kabataan?*	1. Yes<br>2. No						1 -> 402<br>2 -> End of interview
	LINE NUMBER	01	02	03	04	05	06	
402	Now I would like to record all your facility visits for last 12 months. Start with the latest visit you had.<br>Why did you visit a health facility?<br><br>*Subong, imo isulat ang mga pagbisita mo sa health facilty sa nagligad nga dose (12) ka bulan.*<br>*Umpisahan sa pinaka-ulihi mo nga pagbisita*<br><br>AFTER WRITING THE FIRST VISIT IN LINE NUMBER 01, ASK Q403-410 FOR THAT VISIT. THEN ASK THE 2nd LATEST VISIT TO WRITE IN 402 LINE NUMBER 02, THEN ASK Q 403 AND Q404. REPEAT FOR ALL HEALTH FACILITY VISITS FOR LAST 12 MONTHS.<br>IF THERE ARE MORE THAN 6, USE AN ADDITIONAL QUESTIONNAIRE.<br><br>1. Prenatal care<br>2. Giving birth, while a women is still in the facility<br>3. Health check after giving birth, after a woman left the facility<br>4. Receiving vaccination or routine check up for child<br>5. Seeking medical advice or treatment for sickness or injury of **child**							-> 403

	6. Seeking medical advice or treatment for sickness or injury of **herself**<br>7. Adolescent clinic<br>8. Other (specify)							
403	Where did you visit?<br>*Diin ikaw nagbisita?*<br><br>1. National hospital<br>2. Regional hospital/Public medical center<br>3. Provincial hospital<br>4. District hospital<br>5. Municipal hospital<br>6. Rural health unit (RHU)/urban health center(UHC)/Lying-in<br>7. Barangay health station (BHS)<br>8. Barangay supply/service point officer/BHW<br>9. Mobile clinic<br>10. Other (specify. Private facility is included here.)							-> 404
404	At that visit, were you or your sexual partner already using any method to delay or avoid getting pregnant?<br><br>*Sa imo pagbisita, kamo bala sang imo sexual partner, naga gamit sang pamaagi nga mapaatras ukon malikawan ang pagbusong?*<br><br>1. Yes (*Hou)*<br>2. No *(Hindi)*							1 ->405<br>2 -> 406
405	Which method(s) were you using?<br><br>*Ano nga mga pamaagi ang imo ginagamit?*<br><br>WRITE DOWN ALL MENTIONED<br><br>1. Female sterilization<br>2. Male sterilization<br>3. IUD<br>4. Injectable (e.g.DMPA)<br>5. Implants							->406

	6. Patch<br>7. Pill<br>8. Condom<br>9. Female condom<br>10. Diaphragm<br>11. Form/Jelly/Cream<br>12. Mucus/Billings/Ovulation<br>13. Basal body temperature<br>14. Symptothermal<br>15. Standard days method<br>16. LAM<br>17. Calendar/Rhythm/Periodic abstinence<br>18. Withdrawal<br>19. Other traditional method<br>20. Other modern method (specify)							
406	At that visit, did any staff member at the health facility speak to you about family planning methods?<br><br>*Sa pagbisita, may ara miyembro sang health facility naghambal sa imo tungod sa pamaagi sang pagplano sng pamilya?*<br>1. Yes *Hou*<br>2. No *Hindi*							1-> 407<br>2-> 402 next line number
407	After that visit, did you start using any FP method or change from your previous method to a new method?<br><br>*Pagkatapos sang pagbisitam nag-umpisa ka bala gamit sang pamaagi snag pagplano sang pamilya or nag bago sang daan sa bago nga pamaagi?*<br>1. Yes *Hou*<br>2. No *Hindi*							1 -> 409<br>2 ->408
408	If you did not start a new method or change from your previous method, why?<br><br>*Kung wala ka nag umpisa gamit sang pamaagi, or nagbago sa dati nga pamaagi, ngaa?*<br><br>1. No need							

	2. Possible side effects of new method<br>3. New method not available at the facility<br>4. Concerns about risk of pregnancy with new method<br>5. Not enough information<br>6. Could not afford to purchase<br>7. Advice of friends, relatives, neighbours not to start or change<br>8. Husband/partner did not support<br>9. Other (specify):__________							
409	Which FP method did you start using after that visit or which new method did you change to?<br><br>*Ano ang bago nga pamaagi ang imo gin umpisahan gamit pagkatapos sang pagbisita ukon sa ano nga pamaagi ikaw nagsaylo?*<br><br>1. Female sterilization<br>2. Male sterilization<br>3. IUD<br>4. Injectable (e.g.DMPA)<br>5. Implants<br>6. Patch<br>7. Pill<br>8. Condom<br>9. Female condom<br>10. Diaphragm<br>11. Form/Jelly/Cream<br>12. Mucus/Billings/Ovulation<br>13. Basal body temperature<br>14. Symptothermal<br>15. Standard days method<br>16. LAM<br>17. Calendar/Rhythm/Periodic abstinence<br>18. Withdrawal<br>19. Other traditional method<br>20. Other modern method (specify)							-> 402 next line number

END OF THE INTERVIEW

(Katapusan sang pag Interbyu)
