# Supplementary material for: Opportunities lost: Barriers to increasing the use of effective contraception in the Philippines
Source: PLoS One. 2019 Jul 25;14(7):e0218187. doi: 10.1371/journal.pone.0218187 (PMC6657820; doi:10.1371/journal.pone.0218187)
Supplement: S6 Questionnaire — (PDF) [file pone.0218187.s006.pdf]

FACILITY NUMBER:

Region	Province	City/Mun	Brgy	Fac. No

As of February 8, 2017

COVER PAGE

FORM1. Interview women of reproductive age who are not currently pregnant or within 6 weeks of delivery, and desire delaying or limiting childbearing.

[Fill one number for each woman contacted in the order they were contacted at the health facility; if done over several days, continue unique sequence numbers]

Identification of interview place		
Region		
Province		
City/Municipality		
Barangay		
Health facility name		
Home address (for home visit only)		
Latitude and longitude<br>(Use the coordinate of GPS in a mobile phone)		
Interview Record		
Date of interview		
Interviewer's name		
Health facility level where interview took place	1. National hospital<br>2. Regional hospital/Public medical center<br>3. Provincial hospital<br>4. District hospital<br>5. Municipal hospital<br>6. Rural health unit (RHU)/urban health center(UHC)/Lying-in<br>7. Barangay health station (BHS)<br>8. Barangay supply/service point officer/BHW<br>9. Mobile clinic<br>10.Others (specify)	
Clinic where interview took place **(FOR LARGE HOSPITALS)**	1. Reproductive health clinic for postnatal care<br>2. Reproductive health clinic not related to postnatal care<br>3. Receiving vaccination or routine check-up for child<br>4. Seeking medical advice or treatment for sickness or injury of child<br>5. Seeking medical advice or treatment for sickness or injury of herself<br>6. Other (specify)	

REPONDENT NUMBER:

Region	Province	City/Mun	Brgy	Facility no.	Respondent No.

SCREENING FORM

[CONSIDER ALL THE WOMEN AT THE HEALTH FACILITY AS OUR TARGET. THEN WE SCREEN THOSE WOMEN THROUGH Q001-009.]

State: "We would like to start by asking a few questions that determine if you are eligible for the survey."

001	How old were you on your last birthday?<br><br>Antnae edad ka ko myaipos a birthday nga odi na olan a kinimbawataan rka?	Age in completed years	Answer	18-49 years ->002<br>Other -> 009
002	Are you pregnant now?<br>(Stop the interview)<br><br>Baka maogat imanto?	1. Yes<br>2. No<br>3. Unsure		1 ->009<br>2 ->003<br>3 ->003
003	What is the name of your last baby<br><br>Antonaa e ngaran o myaipos a inimbawata aka?<br>Record name	1. Name: (ngaran)_____________<br>2. No previous baby (da wata iyan sa myaipos)		1 ->004<br>2 ->006
004	In what month and year was NAME born?<br><br>Antonaa a olan-olan ago ragon e kinimbawataan ka ki (Name)?<br>If less than 6 weeks . Stop the interview.<br><br>(probe: when is his or her birthday)	Month: __ __<br><br>Year: __ __ __ __		Age ≥6 wks ->005<br>Age<6 wks ->009
005	Has your menstrual period returned since the birth of NAME?<br><br>Ba rka myakauma rka so basa puon ko mimbawata aka si (Name)?	1. Yes<br>2. No		1 ->006<br>2 ->006
006	Now I have some questions about the future. Would you like to have (a/another) child, or would you prefer not to have any (more) children?<br><br>Imanto na paka-isaan nakn ska. Sa gya mga pkaori a gawii, kabaya kapn mbawata paruman? O dikadn kabaya mbawata paruman?	1. Have (a/another) child (makambawata paruman)<br>2. No more/none (di/didn)<br>3. Cannot get pregnant (didn kaogat paroman)<br>4. Undecided / don't know (dipn paka-decide)		1 -> 007<br>2 ->008<br>3 ->009<br>4 ->009
007	Do you want (a/another) child soon?<br><br>Baka kabaya mbawata paroman sa magaan dn aya?	1. Yes (oway)<br>2. No, want to wait (di pasi)		1 ->009<br>2 ->008<br>3- >009

REPONDENT NUMBER:

Region	Province	City/Mun	Brgy	Facility no.	Respondent No.

		3. Don't know (di pn katawan)		
008	Are you or your husband/partner currently doing something or using any method to delay or avoid getting pregnant?<br><br>Bakanu pagusar imanto ki karumangka sa mga okit a para dika pasi maogat?	1. Yes (oway)<br>2. No (di)		1 -> 101<br>2 -> 101<br>To achieve a total of 5 users and non-users (hospitals) and 3 users and 3 non-users (health centres).
009	Thank the woman, indicate ineligibility for the survey and stop the interview. **[FILE THE SCREENING PAGE IN THE ENVELOPE MARKED "*SCREENING FORM*"**. Enter this woman into "number of women contacted". Then find another woman to interview and use another screening sheet.<br><br>Salamat. Panalamatan nakn ska ka ko kya-iyog ka a manumbag para ko mga paka-I'sa akn.			

REPONDENT NUMBER:

Region	Province	City/Mun	Brgy	Facility no.	Respondent No.

QUESTIONNAIRE

FORM1. Interview of women of reproductive age who are not currently pregnant or within 6 weeks of delivery, and desire delaying or limiting childbearing.

NO.	Section 1. Respondent background			
101	In (month of interview) 2017, did you live in a city, in a town proper/ *Poblacion*, in the barrio or rural area, or abroad?<br><br>Sangkaya a ragon a 2017 (olan o interview), andakano mababaling? Sa city, sa poblacion, sa baryo, sa ingud o sa abroad?	1. City (City)<br>2. Town Proper/*Poblacion*<br>3. Barrio/Rural Area<br>4. Abroad (*Abroad*)<br>5. Don't Know (di katawan)		->102
102	What is your marital status now?<br><br>Baka myakabangon sa walay?	1. Never married or never lived with a man (dadn mapangaruma)<br>2. Currently married (and a karuma niyan)<br>3. Currently living with a man (adn a apda iyan a mama)<br>4. Divorced/separated/widow and not currently living with a man (bitowan'n, balo, da apda iyan a mama)		->103
103	What is your highest level of education attended, whether or not that level was completed?<br><br>Antnae pinakamatas a kyapageswelaan ka, myapasad ka aya o da?	1. No education (da myapasad iyan/da makapageskwela)<br>2. Elementary (elementary)<br>3. High school (highschool)<br>4. College (college)<br>5. Post-graduate (master)		->104
104	How many children do you have who are still alive?<br><br>Pira katao e sisaya a wata aka?	Number of children alive<br><br>(Kadakl o wata a myaoyag)		->105
105	Women sometimes have pregnancies that do not result in a live born child. That is, a pregnancy can end early, in a miscarriage or the child can be born dead. Have you ever had a pregnancy that did not end in a live birth?<br>Egira na pkasagadan o mga bae a mga oogat a di kaoyag so ikaoogat iran a wata. Gyanan so, ikaoogat a pkabobos, odi na da kaoyagi ko kinimbawata anon. Ba adn a a inikaogat ka a da maoyag?	1. Yes (oway, adn)<br>2. No (da)		1 ->106<br>2 -> 107

REPONDENT NUMBER:

Region	Province	City/Mun	Brgy	Facility no.	Respondent No.

106	In all, how many pregnancies have you had that did not end in a live born child?<br><br>Mga pira katao e inikaogat ka a da maoyag?<br>**[WRITE DOWN NUMBER OF SPONTANEOUS ABORTION AND INDUCED ABORTION SEPARATELY] (suratn ka so kadakl o myabobos ago so myatibaba a kyabobos iyan)**	A. Number of pregnancy losses by nalaglag (spontaneous abortion) (kadakl o ikaoogat a myabobos)<br><br>B. Number of pregnancy losses by (induced abortion) (kadalk o ikaoogat a myatibaba a kyabobos iyan?)		->107
107	Are you covered by any health insurance, either as member or dependent?<br><br>Ba adn a health insurance iyo? Datar opama o philheath, odi na ko pamakapoon sa goberno datar a gsis, sss?<br>**[WRITE DOWN ALL MENTIONED.]**	1. Not covered **(da)**<br>2. Philhealth<br>3. Government Service Insurance System (GSIS)<br>4. Social Security System (SSS)<br>5. Private insurance company/Health (maintenance organization /Pre-need insurance plan company<br>6. Other (Specify)		->201
**NO.**	**Section 2. Current use of FP**			
201	REVIEW: Are you or your husband/partner currently doing something or using any method to delay or avoid getting pregnant?<br>Bakanu imanto pagosar ki karomangka sa okit a dingka pasi kaogat?	1. Yes (oway)<br>2. No (di)		1 ->202<br>2 -> 206
202	Which method are you currently using?<br>Antna e okit a pagusarn iyo?<br>**[WRITE DOWN ALL MENTIONED.]**	1. Female sterilization (kapakilaygit o babay)<br>2. Male sterilization (kapakilaygit o mama)<br>3. IUD<br>4. Injectable (e.g., DMPA) (kapaki-injection)<br>5. Implants<br>6. Patch<br>7. Pill (kapanginom sa pills)<br>8. Male Condom (kaosar o mama sa condom)<br>9. Female condom (kaosar o babay sa condom)<br>10. Diaphragm<br>11. Foam/Jelly/Cream<br>12. Mucus/Billings/Ovulation<br>13. Basal body temperature<br>14. Symptothermal<br>15. Standard days method		->203

REPONDENT NUMBER:

Region	Province	City/Mun	Brgy	Facility no.	Respondent No.

		16. LAM<br>17. Calendar/Rhythm/Periodic abstinence<br>18. Withdrawal<br>19. Other traditional method<br>20. Other modern method (specify)					
	LINE NUMBER	01	02	03	04	05	
203	Now I would like to ask you one by one about all methods you are using now.<br>Imanto na pnggaga-isa-an nakn rka maka-isa so mga okit a pagusarn nka para s aka-control.<br>**[RECORD ALL METHODS BEING USED NOW, ONE METHOD PER ONE LINE NUMBER.<br>IF THERE ARE MORE THAN 5 METHODS, USE ADDITIONAL QUESTIONNAIRE.]**<br>***(show card)***<br>1. Female sterilization<br>2. Male sterilization<br>3. IUD<br>4. Injectable (e.g., DMPA)<br>5. Implants<br>6. Patch<br>7. Pill<br>8. Male Condom<br>9. Female condom<br>10. Diaphragm<br>11. Foam/Jelly/Cream<br>12. Mucus/Billings/Ovulation<br>13. Basal body temperature<br>14. Symptothermal<br>15. Standard days method<br>16. LAM<br>17. Calendar/Rhythm/Periodic abstinence<br>18. Withdrawal<br>19. Other traditional method<br>20. Other modern method (specify)						->204
204	Where did you obtain that method when you first started using it?<br>Anda nga paganay a myakowa gyanan a okit a pagosarn nka sa ka-control?<br>**Public Sector**<br>1. Government hospital<br>2. Rural health unit/Urban health center<br>3. Barangay health station						->205

	4. Barangay supply/Service point office/BHW<br>5. Other public<br>**Private medical sector**<br>6. Private hospital/clinic<br>7. Pharmacy<br>8. Private doctor<br>9. Private nurse, midwife<br>10. NGO<br>11. Industry-based clinic<br>12. Other private<br>**Other source**<br>13. Puericulture center<br>14. Store<br>15. Church<br>16. Friends/Relatives						
205	What was the purpose of your going to the health facility on the day you first received the contraceptive method**?**<br><br>**Antonaa oto e purpose a kyasong ka sa clinic/center ko gawii a kyapakaresib ka mambo sa mga pagosarn nka sa ka-control?**<br><br>1. Prenatal care (kapaki-check upko mga paganay a olan o kaogat)<br>**2.** Giving birth, while a woman is still in the facility (kambawata, kapapantagan a sisa clinic/center/ospital)<br>3. Reproductive health outpatient clinic for postnatal care (kapaki-check up ko myakambawata)<br>4. Reproductive health clinic not related to postnatal care (Kapaki-check up pero knaba mipantag ko kambawata)<br>**5.** Receiving vaccination or routine check- up for child (Kapakibakona ko wata odi na so kalalayaman a kapaki-check-up'n ko wata)<br>6. Seeking medical advice or treatment for sickness or injury of child **(Kakowa sa adbais ko sakit odi na so mga pd a pkagdam o wata)**<br>*7.* Seeking medical advice or treatment for sickness or injury of						-> 203. Repeat until all methods were explained<br>.<br><br>Then ->206

REPONDENT NUMBER:

Region	Province	City/Mun	Brgy	Facility no.	Respondent No.

	herself **(kakowa sa adbais ko kapakapia o sakit odi na so mga pd a pkagdam ka sa ginawangka)**<br>8. Adolescent clinic (para ko mga pmamangoda ago so pragaraga a mga wata)<br>9. Other (specify) (oba adn pn a sabagi a rason)						
206	If you <u>are not</u> using any method to delay or avoid getting pregnant now, have you or your sexual partner done something or used a method to delay or avoid getting pregnant in the past?<br><br>Odi ka pagosar imanto sa okit a dingka kaogat, na antonae pd a mga okit a psowaan iyo odi na inosar iyo ki karomangka para di ka pasi maogat?<br><br>If <u>you are</u> using a method to delay or avoid getting pregnant now, have you or your sexual partner ever used a different method to delay or avoid getting pregnant in the past?<br><br>O pagusar ka imanto sa okit a dingka kaogat, na antnae pd a mga okit a psuwaan iyo odi na inosar iyo ki karomangka para di ka pasi maogat?				1. Yes<br>2. No		1->207<br>2-> 301
207	Which methods have you used in the past?<br>**Antonaa a mga okit-okit a inosar ka a ka-control sa myanga-iipos a gawii?**<br>**[WRITE DOWN ALL MENTIONED.]**	1. Female sterilization<br>2. Male sterilization<br>3. IUD<br>4. Injectable (e.g., DMPA)<br>5. Implants<br>6. Patch<br>7. Pill<br>8. Male Condom<br>9. Female condom<br>10. Diaphragm<br>11. Foam/Jelly/Cream<br>12. Mucus/Billings/Ovulation<br>13. Basal body temperature<br>14. Symptothermal<br>15. Standard days method<br>16. LAM<br>17. Calendar/Rhythm/Periodic abstinence<br>18. Withdrawal<br>19. Other traditional method<br>20. Other modern method (specify)					-> 208
	LINE NUMBER	01	02	03	04	05	

REPONDENT NUMBER:

Region	Province	City/Mun	Brgy	Facility no.	Respondent No.

208	Now I would like to ask you one by one about all methods you have used in the past.<br>Imanto na pnggaga-isa-an nakn rka maka-isa so mga okit a ka-control a inosar ka sa myanga-iipos a gawii para di ka pasi maogat?<br><br>**[RECORD ALL METHODS, ONE METHOD PER ONE COLUMN NUMBER AT THE RIGHT.<br>IF THERE ARE MORE THAN 5 METHODS, USE ADDITIONAL QUESTIONNAIRE.]**<br><br>1. Female sterilization<br>2. Male sterilization<br>3. IUD<br>4. Injectable (e.g., DMPA)<br>5. Implants<br>6. Patch<br>7. Pill<br>8. Male Condom<br>9. Female condom<br>10. Diaphragm<br>11. Foam/Jelly/Cream<br>12. Mucus/Billings/Ovulation<br>13. Basal body temperature<br>14. Symptothermal<br>15. Standard days method<br>16. LAM<br>17. Calendar/Rhythm/Periodic abstinence<br>18. Withdrawal<br>19. Other traditional method<br>20. Other modern method (specify)						-> 209
209	Where did you obtain the family planning method when you first started using it?<br><br>Anda nga kyatokawan gyae a mga okit family planning ko paganay a kyaosar kawn?<br><br>Public Sector<br>1. Government hospital<br>2. Rural health unit/Urban health center<br>3. Barangay health station<br>4. Barangay supply/Service point office/BHW<br>5. Other public<br><br>Private medical sector<br>6. Private hospital/clinic						-> 210

REPONDENT NUMBER:

Region	Province	City/Mun	Brgy	Facility no.	Respondent No.

	7. Pharmacy<br>8. Private doctor<br>9. Private nurse, midwife<br>10. NGO<br>11. Industry-based clinic<br>12. Other private<br><br>Other source<br>13. Puericulture center<br>14. Store<br>15. Church<br>16. Friends/Relatives<br>(dimbawataan) (botika)						
210	Why did you visit the health facility where you first started using the family planning method?<br><br>**Antonaa oto e purpose a kyasong ka sa clinic/center ko paganay mambo a kyaosar ka sa okit a family planning?**<br><br>1. Prenatal care<br>2. Giving birth, while a women is still in the facility<br>3. Reproductive health outpatient clinic for postnatal care<br>4. Reproductive health clinic not related to postnatal care<br>5. Receiving vaccination or routine check-up for child<br>6. Seeking medical advice or treatment for sickness or injury of child<br>7. Seeking medical advice or treatment for sickness or injury of herself<br>8. Adolescent clinic<br>9. Other (specify)						-> 211
211	Why did you stop using the family planning method that you used in the past?<br><br>Inongka nggn'ki o targi so myanga-iipos a inosar ka okit a family planning?<br>1. Side effects (so mga rarata a side epek iyan)<br>*2.* Method not available at the facility (di available sa center/clinic)<br>3. Concerns about risks of pregnancy (p'kawan ko mga pakal'k-l'k a epek iyan ko maogat)						-> 212

REPONDENT NUMBER:

Region	Province	City/Mun	Brgy	Facility no.	Respondent No.

	4. Could not afford to purchase (di kalotang)<br>*5.* Health worker did not continue to provide the method. (dadn tarosn o di mamgay sa supply)<br>6. Quality of care provided by the health facility or provider. (di mapia kurang so quality ago serbisyo o diron di mamgay)<br>*7.* Advice of friends, relatives, neighbors (thoma o mga ginawae, tonganay ago mga siringan)<br>8. Husband/partner did not support or allow to use (di makaaayon si mister sa kapagosara-on)<br>9. Wanted to get pregnant (kabaya pn maogat paroman)<br>10. Other (specify):___________							
	**Section 3. FP Concerns and Today's FP counseling**							
301	Do you have any health concerns about any type of family planning method?<br><br>Ba adn a mga concern nka ko mga pitibarangan a klase o okit a kapag family planning?	1. Yes<br>2. No						1 ->302<br>2 ->305
	LINE NUMBER	01	02	03	04	05	06	
302	What are your health concerns about family planning methods?<br><br>Antnaa so mga concernka sangkae a family planning ko para kambolawasan tanu?<br>Please tell me one by one.<br>Nggaga-isangka so mga concern ka.<br><br>**[USE ONE LINE NUMBER FOR ONE CONCERN. WRITE DOWN ALL MENTIONED CONCERNS.**<br>**IF THERE ARE MORE THAN 6 CONCERNS, USE ADDITIONAL QUESTIONNAIRE.]**<br><br>1. Cause cancer in the uterus (Paka-adn sa cancer ko matris)<br>2. Cause cysts in the uterus (Paka-adn sa cysts ko matris)<br>3. Cause infection of the uterus (pka-impeksyon so matris)<br>*4.* Cause frequent bleeding (pkabundas a rugo)							-> 303

REPONDENT NUMBER:

Region	Province	City/Mun	Brgy	Facility no.	Respondent No.

	5. Cause thyroid problems (pka-adn a problema sa bak'rng)<br>6. Cause/worse asthma (pka-adn a asma/kapakala o asma)<br>7. Cause/worsen lots of veins (pkabinasa niyan so mga ogat)<br>8. Cause dry skin, skin disease (pkamaraan so sapo, pka-adn so mga sakit sa sapo)<br>9. Cause weight<br>10. Cause weight loss (pakababa so timbang o lawas)<br>*11.* Cause bloated stomach (maka-ala so tiyan)<br>*12.* Cause headache (pka-adn so sakit a ulo)<br>*13.* Cause irritability (makaggdam sa rarangit)<br>*14.* Increase libido/turn into a maniac (pakala so kababaya/dimbaloy a manyak)<br>15. Cause loss/reduce of libido (kapkhada/phakaito so kababaya)<br>16. Cause loss/reduce of sexual satisfaction (pkada/phakaito so kababaya ko kapakikaruma)<br>17. One will not have children anymore (so isa na pkabaog)<br>18. Not fully effective, woman could still get pregnant (da kabisa niyan ka pkaogat bo sa babay)<br>19. When it does not work, the baby is born with abnormalities (egira da kabisa niyan na makaliyo odi na mimbawata so wata na di normal, adn a mga kurangon odi na mga abnormalities iyan)<br>20. Results in mortal sin because it is against church teachings (kapkadusa o manusiya)<br>21. It is not legally allowed to use (inisapar oba pagusara)<br><br>IUD/Implants<br>22. Melt or move around inside the body and doctors will not be able to find. (pkatonag odi na pkadadag sa sold a lawas a ukit a dirun kakhatuona o doctor)<br>23. Washed away/pushed out of body. (pakaliyo sa lawas)<br>24. Painful to insert (masakit egira ipsukaron)						

REPONDENT NUMBER:

Region	Province	City/Mun	Brgy	Facility no.	Respondent No.

	IUD<br>25.Itchy on the vagina. (pakagatl so poki)<br>26.Entangled around the man's penis (pkisadang ko oten)<br>27.Messy when inserted (marsik egira a misosold-on)<br><br>Male Sterilization<br>28.Part of the man's testicles are cut off (adn a putoln ko oten o mama)<br>29.It hurts the testicles (pkasakitan so oten)<br>30.The man loses his manhood ("kapon") (pkada so kamamamae)<br>31. Others (specify) (so mga sabagi pn)							
303	About which family planning methods do you have concerns***?***<br><br>Antnae ko mga okit o family planning e and a concern kawn?<br>**[REPEAT EACH CONCERN IN TURN. FOR EACH CONCERN, WRITE DOWN ALL METHODS CAUSING THAT CONCERN.]**<br><br>1. Female sterilization<br>2. Male sterilization<br>3. IUD<br>4. Injectable<br>5. Implants<br>6. Patch<br>7. Pill<br>8. Other modern method (specify)<br>9. Other method (specify)							-> 304
304	Who told you or how did you find about your concerns about family planning methods?<br><br>Antawaa e myakatharo rkawn? Odi na andamanaya e kyatokawingka sa gyae a mga concern nka ko family planning?<br>**[REPEAT EACH CONCERN IN TURN. FOR EACH WRITE DOWN ALL SOURCES OF INFORMATION.]**<br><br>1. Health staff (gumagalbk sa clinic/center)							-> 305

REPONDENT NUMBER:

Region	Province	City/Mun	Brgy	Facility no.	Respondent No.

	2. BHW or health volunteers (BHW Volunteers)<br>3. Husband or partner did not want to use (di makaayonsi mister/karua sa kapagusara-on)<br>4. Friend, neighbours, relatives (siko mga ginawae, siringan, tunganay)<br>5. Church (sa masget)<br>6. Radio (sa radyo)<br>7. Television (sa TB)<br>8. Newspaper or magazine (sa news/magasin)<br>9. Online or internet (sa internet)<br>10. Others (specify) (ko mga sabagi pn a okit)							

305	Today, did any staff member at the health facility speak to you about family planning methods?<br>Imanto na ba adn a gomagalbk sa clinic/center a myakatharo rka sangkae a mga okit a kapag-family planning?	1. Yes<br>2. No		1 ->306<br>2 ->401
306	Did the health worker ask you about your concerns**?**<br>**Ba kaniyan pyaka-isaan ko mga concern ka?**	1. Yes<br>2. No		1 ->307<br>2 ->409
307	Do you feel the health worker understands your concerns?<br>Sasabotn niyan mangaday so mga concern ka?	1. Yes<br>2. No		->308
308	Did the health worker help you to find solutions to your concerns?<br>Inugupan ka niyan mangiloba sa okit odi na solusyon sangkanan a concern ka?	1. Yes<br>2. No		->309
309	Did the health worker offer you information how different family planning methods work?<br>Ba and a mga inipamgay niyan rka a mga pamagadanadan ko kapag-family planning?	1. Yes<br>2. No		1 ->310<br>2 ->312
310	Which methods did health worker mention today?<br>Antnae mga okit okit o kapag-family planning a mya-aloy niyan rka?	1. Female sterilization<br>2. Male sterilization<br>3. IUD<br>4. Injectable (e.g., DMPA)<br>5. Implants<br>6. Patch<br>7. Pill<br>8. Male Condom<br>9. Female condom<br>10. Diaphragm<br>11. Foam/Jelly/Cream<br>12. Mucus/Billings/Ovulation		->311

REPONDENT NUMBER:

Region	Province	City/Mun	Brgy	Facility no.	Respondent No.

		13. Basal body temperature<br>14. Symptothermal<br>15. Standard days method<br>16. LAM<br>17. Calendar/Rhythm/ Periodic abstinence<br>18. Withdrawal<br>19. Other traditional method<br>20. Other modern method (specify)		
311	Did the health worker tell you about side-effects or problems you might have with any methods of family planning?<br>Ba niyan rka myaaloy gyuto a gumagalbk sa center/clinic so mga di mapia a epek odi na mga kabaloy a problema ko kapag-family planning?	1. Yes<br>2. No		-> 312
312	Did the health worker offer you information how your family planning method works?<br>Ba rka myaaloy gyuto a gumagalbk sa clinic/center so mga pamaganadan odi na mga nda-o o kapag-family planning?	1. Yes<br>2. No<br>3. N/A (not using a method now)		1 -> 313<br>2-> 313<br>3-> 315
313	Did the health worker explain about the side effects of your current method?<br>Ba rka myanggogod o gumagalbk sa clinic/center so mga di mapia a epek o kapag-family planning?	1. Yes<br>2. No		-> 314
314	Did the health worker ask you to describe how you use your current method?<br>Ba rka inipaka-isa o gumagalbk sa clinc/center so okit a pagusarn nka imato para ko kapag-family planning?	1. Yes<br>2. No		-> 401
315	After receiving FP counselling will you begin using a family planning method today?<br><br>Siko kyapakaresib ka sa mga ilmo/nda-o mipantag ko kapag-family planning, na baka kabaya mag family planning poon imanto?	1. Yes<br>2. No		1 -> 317<br>2 -> 316
316	After receiving FP counselling will you begin using, do you think you will use a contraceptive method anytime in the future?<br>Siko kyapakaresib ka sa mga ilmo/nda-o mipantag ko kapag-family planning, na bangka pkapikir imanto a pagusar kadn sa mga okit a ka-control sa gya mga pkaori a gawii?	1. Yes<br>2. No		1 -> 317<br>2 -> 316
317	Which contraceptive method would you prefer to use?			-> 401

REPONDENT NUMBER:

Region	Province	City/Mun	Brgy	Facility no.	Respondent No.

	Antna okit a ka-control e kabaya aka a pagosarn?	1. Female sterilization<br>2. Male sterilization<br>3. IUD<br>4. Injectable (e.g.DMPA)<br>5. Implants<br>6. Patch<br>7. Pill<br>8. Male Condom<br>9. Female condom<br>10. Diaphragm<br>11. Foam/Jelly/Cream<br>12. Mucus/Billings/Ovulation<br>13. Basal body temperature<br>14. Symptothermal<br>15. Standard days method<br>16. LAM<br>17. Calendar/Rhythm/ Periodic abstinence<br>18. Withdrawal<br>19. Other traditional method<br>20. Other modern method (specify)		

	Section 4. Past Health facility visit and FP counseling								
401	From January 1- December 31, 2016, have you visited a health facility for care for yourself or your children for any purpose?<br><br>Poon ko January 1 taman ko December 2016, na ba adn a sabap a myakasong ka sa clinic/center/ospital para ko ginawangka odi na ko mga wata aka?	1. Yes<br>2. No			1 -> 402<br>**2 -> END OF INTERVIEW**				
	LINE NUMBER	01	02	03	04	05	06		
402	Now I would like to record all your facility visits from January 1 – December 31, 2016.<br>Imanto na o kapakay na iprecord akn so mga gawii a kyapakasong ka sa clinic/center/ospital siko myaipos a sapolo ago dowa (12) a olan, January 1 taman ko December 31, 2016.<br>Start with the latest visit you had.<br>Poon ko pinakabago a kyapakasong ka ruo.<br>Why did you visit a health facility?							-> 403	

REPONDENT NUMBER:

Region	Province	City/Mun	Brgy	Facility no.	Respondent No.

	Antnae sabap a kyasong ka ruo sa clinic/center/ospital?<br><br>**[AFTER WRITING THE FIRST VISIT IN LINE NUMBER 01, ASK Q403-410 FOR THAT VISIT. THEN ASK THE 2nd LATEST VISIT TO WRITE IN 402 LINE NUMBER 02, THEN ASK Q 403 AND Q404.**<br>**REPEAT FOR ALL HEALTH FACILITY VISITS FROM JANUARY 1 - DECEMBER 31, 2016.]**<br><br>**[IF THERE ARE MORE THAN 6, USE AN ADDITIONAL QUESTIONNAIRE.]**<br><br>1. Prenatal care (Kapaki check-up ko mga paganay a olan o maogat)<br>*2.* Giving birth, while a woman is still in the facility (kambawata, egira so babay na sisa dimbawataan)<br>*3.* Reproductive health outpatient clinic for postnatal care (kapaki check-up egira myakambawata)<br>*4.* Reproductive health clinic not related to postnatal care (kapaki check-up a knaba mipantag ko kyambawata)<br>*5.* Receiving vaccination or routine check- up for child (kapakibakuna ko wata odi na so kalalayaman a kapaki chek-up'n ko wata)<br>*6.* Seeking medical advice or treatment for sickness or injury of child (Kakowa sa adbais ko sakit odi na mga pd a pkagdam o wata)<br>*7.* Seeking medical advice or treatment for sickness or injury of herself (kakowa sa adbais ko kapakapia o sakitago so pd a pkagdam ka sa ginawangka)<br>*8.* Adolescent clinic (para ko pmamangoda/pragaraga a clinic)<br>*9.* Other (specify) (so sabagi pn a rason)							
403	Where did you visit? And aka myamisita a clinic/center/ospital?<br>1. National hospital<br>2. Regional hospital/Public medical center<br>3. Provincial hospital<br>4. District hospital<br>5. Municipal hospital<br>6. Rural health unit (RHU)/urban health center (UHC)/Lying-in<br>7. Barangay health station (BHS)<br>8. Barangay supply/service point officer/BHW<br>9. Mobile clinic<br>10.Other (specify. Private facility is included here.)							-> 404
404	At that visit, were you or your sexual partner already using any method to delay or avoid getting pregnant?							1 -> 405<br>2 -> 406

REPONDENT NUMBER:

Region	Province	City/Mun	Brgy	Facility no.	Respondent No.

	Siko kyapamisita nga sa clinic/center/ospital na bakanodn pagosar ki karumangka sa mga okit a para di ka pasi maogat?<br>1. Yes (oway)<br>2. No (di)							
405	Which method(s) were you using?<br>Antnae klase niyan a mga okit-okit e agusarn iyo?<br>**[WRITE DOWN ALL MENTIONED.]**<br><br>1. Female sterilization (kapakilaygit o babay)<br>2. Male sterilization (kapakilaygit o mama)<br>3. IUD<br>4. Injectable (e.g., DMPA)<br>5. Implants<br>6. Patch<br>7. Pill<br>8. Male Condom<br>9. Female condom<br>10. Diaphragm<br>11. Foam/Jelly/Cream<br>12. Mucus/Billings/Ovulation<br>13. Basal body temperature<br>14. Symptothermal<br>15. Standard days method<br>16. LAM<br>17. Calendar/Rhythm/Periodic abstinence<br>18. Withdrawal<br>19. Other traditional method<br>20. Other modern method (specify)							->406
406	At that visit, did any staff member at the health facility speak to you about family planning methods?<br>1. Ko maipos a kyamapimistangka sa clinic/center/ospital, na ba and a staff odi na gomagalbk ruo a myakapagistorya rka mipantag sa gyae a family planning?Yes<br>2. No							1-> 407<br>2-> 402 next line number
407	After that visit, did you start using any FP method or change from your previous method to a new method?<br>Ko myaipos a kyapamisitangka sa ospital/clinic/center, na b aka myagpoon mag family planning? Odi na ba and a mga byago nga ko myanga-oona a pagusarn ka?<br>1. Yes<br>2. No							1 -> 409<br>2 ->408
408	If you did not start a new method or change from your previous method, why?							

REPONDENT NUMBER:

Region	Province	City/Mun	Brgy	Facility no.	Respondent No.

	Angkainoto, ino da ka osar sab ago a kpag-family planning? Odi na ino ka da sambi sa okit-okit a kapag-family planning?<br><br>1. No need (didn kailangan)<br>*2.* Possible side effects of new method (And a mga possible a mga rarata a side epek iyan)<br>*3.* New method not available at the facility (so mga bago a okit a kapag-family planning na da available on.<br>4. Concerns about risk of pregnancy with new method (pkawan ko mga pakal'k-l'k a epek iyan ko maogat)<br>*5.* Not enough information (kurang pn so mga nda-o odi na mga impormasyon mipantag sa gyae a kapag-family planning)<br>6. Could not afford to purchase (di kalutang)<br>*7.* Advice of friends, relatives, neighbours not to start or change (inithoma o mga ginawae, tunganay, siringan a didn tpngan so kapag-family planning)<br>8. Husband/partner did not support (di-makaayon so karomangka)<br>9. Others (specify): ___________(o ba adn pn a mga sabagi a arason)							
409	Which FP method did you start using after that visit or which new method did you change to?<br>Antana okit-okit ko kapag-family planning e inosar ka kayko makapoon ka sa clinic/center/ospital. Odin a antnae mya byago nga ko pagusarn ka?<br>1. Female sterilization<br>2. Male sterilization<br>3. IUD<br>4. Injectable (e.g., DMPA)<br>5. Implants<br>6. Patch<br>7. Pill<br>8. Male Condom<br>9. Female condom<br>10. Diaphragm<br>11. Foam/Jelly/Cream<br>12. Mucus/Billings/Ovulation<br>13. Basal body temperature<br>14. Symptothermal<br>15. Standard days method<br>16. LAM<br>17. Calendar/Rhythm/Periodic abstinence<br>18. Withdrawal<br>19. Other traditional method<br>20. Other modern method (specify)							-> 402 next line number

REPONDENT NUMBER:

Region	Province	City/Mun	Brgy	Facility no.	Respondent No.

END OF THE INTERVIEW
