# Supplementary material for: Opportunities lost: Barriers to increasing the use of effective contraception in the Philippines
Source: PLoS One. 2019 Jul 25;14(7):e0218187. doi: 10.1371/journal.pone.0218187 (PMC6657820; doi:10.1371/journal.pone.0218187)
Supplement: S7 Questionnaire — (PDF) [file pone.0218187.s007.pdf]

REPONDENT NUMBER:

Region	Province	City/Mun	Brgy	No

COVER PAGE

FORM1. Interview women of reproductive age who are not currently pregnant or within 6 weeks of delivery, and desire delaying or limiting childbearing **(Interbyu ng mga kababaihan na nasa edad na maaring magkaanak pero hindi buntis sa ngayon o kapapanganak lang sa loob ng anim na buwan at nais na madelay o limitahan ang magbuntis.)**

Sequence number: _____________Region/Province/Municipality/Barangay Number

[Fill one number for each woman contacted in the order they were contacted at the health facility; if done over several days, continue unique sequence numbers]

Identification of interview place		
Region		
Province		
CITY/MUNICIPALITY		
BARANGAY		
Health facility name		
Home address (for home visit only)		
Latitude and longitude<br>(Use the coordinate of GPS in a mobile phone)		
Interview Record		
Date of interview		
Interviewer's name		
Health facility level where interview took place	1. National hospital<br>2. Regional hospital/Public medical center<br>3. Provincial hospital<br>4. District hospital<br>5. Municipal hospital<br>6. Rural health unit (RHU)/urban health center(UHC)/Lying-in<br>7. Barangay health station (BHS)<br>8. Barangay supply/service point officer/BHW<br>9. Mobile clinic<br>10. Others (specify)	
Clinic where interview took place **(FOR LARGE HOSPITALS)**	1. Reproductive health clinic for postnatal care<br>2. Reproductive health clinic not related to postnatal care<br>3. Receiving vaccination or routine check-up for child<br>4. Seeking medical advice or treatment for sickness or injury of child<br>5. Seeking medical advice or treatment for sickness or injury of **herself**<br>6. Other (specify)	

REPONDENT NUMBER:

Region	Province	City/Mun	Brgy	No

Sequence Number: _________________
[Write the same sequence number from Cover Page]

Instructions:
Read the information sheet. Answer questions. If the woman agrees to participate give the certificate of consent for her to sign. Then start the Screening Form.

Screening Form (Can we consider all the women visiting and as the list frame because since we don't know if she is qualified or not.)

State: "We would like to start by asking a few questions that determine if you are eligible for the survey." (Nais ko pong magtanong ng ilang mga katanungan para alamin kung kayo po ay angkop na interbyuhin para sa survey na ito.)

001	How old were you on your last birthday? (**Ilang taon napo kayo noong huling birthday?)**	Age in completed years **(Edad)**		18-49 years ->002<br>Other -> 009
002	Are you pregnant now? **(Kayo po ba ay nagdadalantao/Buntis po kayo ngayon?) (Stop the interview)**	**1.** Yes **(Oo)**<br>**2.** No **(Hindi)**<br>3. Unsure **(Hindi SIgurado)**		1 ->009<br>2 ->003<br>3 ->003
003	What is the name of your last baby? **(Ano po ang pangalang ng inyong pinakabatang anak?)** Record name	1. Name: ____________ **(Pangalan)**<br>2. No previous baby		1 ->004<br>2 ->006
004	In what month and year was NAME born? (Ano po ang buwan at taon ipinanganak si _________) If less than 6 mos. Stop the interview.<br><br>(probe: when is his or her birthday) **(PROBE*: Kailan po ang birthday ni* ___________**)	Month **(Buwan):** __ __<br><br>Year **(Taon):** __ __ __ __		Age ≥6 wks ->005<br>Age<6 wks ->009
005	Has your menstrual period returned since the birth of NAME? (**Bumalik na po ba ng inyong regla pagkatapos manganak kay _________?)**	1. Yes **(Oo)**<br>**2.** No **(Hindi)**		1 ->006<br>2 ->006
006	Now I have some questions about the future. Would you like to have (a/another) child, or would you prefer not to have any (more) children? **(Ngayon naman po, may ilan po akong katanungan tungkol sa mga darating na panahon. Gusto po ba ninyo**	1. Have (a/another) child **(Magkaroon ulit ng anak)**<br>**2.** No more/none **(Hindi na)**<br>**3.** Cannot get pregnant **(Hindi na pwede manganak)**		1 -> 007<br>2 ->008<br>3 ->009<br>4 ->009

	**pang magkaroon uli ng anak o mas gugustuhin ninyo na di na magkaanak?**	4. Undecided / don't know **(Hindi ko alam)**		
007	Do you want (a/another) child soon? **(Gusto po ba ninyo na magkaanak na agad?)**	**1.** Yes **(Oo)**<br>**2.** No, want to wait **(Hindi pa, gusto ko pang maghintay)**<br>3. Don't know **(Hindi ko alam)**		1 ->009<br>2 ->008<br>3- >009
008	Are you or your husband/partner currently doing something or using any method to delay or avoid getting pregnant? **(Kayo po ba o ang inyong asawa/partner ay may ginagamit na paraan para madelay o maiwasan ang magbuntis?)**	**1.** Yes **(Oo)**<br>2. No **(Hindi)**		1 -> 101<br>2 -> 101<br>To achieve a total of 5 users and non-users (hospitals) and 3 users and 3 non-users (health centres).
009	Thank the woman, indicate ineligibility for the survey and stop the interview. Enter this woman into "number of women contacted". Then find another woman to interview. **(PASALAMATAN ANG KAUSAP, SABIHAN NA TAPOS NA ANG INTERVIEW. ILAGAY ANG PANGALAN SA LISTAHAN NG MGA BABAE NA KINAUSAP". MAGHANAP ULIT NG SUSUNOD NA IINTERBYUHIN.**			

REPONDENT NUMBER:

Region	Province	City/Mun	Brgy	No

QUESTIONNAIRE

FORM1. Interview of women of reproductive age who are not currently pregnant or within 6 weeks of delivery, and desire delaying or limiting childbearing. ***(INTERBYU NG MGA KABABAIHAN NA NASA EDAD NA MAARING MAGANAK PA PERO HINDI BUNTIS SA KASALUKUYAN O KAPAKAPANGANAK LANG SA LOOB NG ANIM NA BUWAN, AT NAGNANAIS NA IDELAY O INIIWASAN ANG MALIMIT NA PAG-BUBUNTIS.)***

Sequence Number: ____________________
[Write the same sequence number from Cover Page]

NO.			
101	In (month of interview) 2017, did you live in a city, in a town proper/ poblacion, in the barrio or rural area, or abroad? **(Ngayon pong ________ 2017 ________________, kayo po ba ay nakatira sa syudad o bayan, sa barangay o sa ibang bansa?)**	**1.** City (City) ***(Syudad)***<br>2. TOWN PROPERIPOBLACION **(*Bayan/Poblacion*)**<br>3. BARRIO/RURAL AREA **(Barangay)**<br>4. ABROAD (*Abroad*)<br>5. DON'T KNOW **(Hindi alam.)**	->102
102	What is your marital status now? **(Kayo po ba ay __________________ sa kasalukuyan**	1. Never married or never lived with a man (***walang asawa o hindi nagkaroon ng kinakasama)***	->103
		2. Currently married **(*Kasalukuyang may asawa)***	
		3. Currently living with a man **(Kasalukuyang may kalive-in)**	
		4. Divorced/separated/widow and not currently living with a man **(Diborsyado/hiwalay/balo at kasalukuyang walang ka live-in)**	
103	What is your highest level of education attended, whether or not that level was completed? ***(Ano po ang inyong natapos na antas ng pag-aaral?)***	1. No education **(Wala)**<br>2. Elementary **(Elementarya)**<br>3. High school **(Mataas na Paaralan)**<br>**4.** College **(Kolehiyo)**<br>5. Post-graduate **(Masteral o Ph D)**	->104
104	How many children do you have who are still alive? (**Ilan po ang inyong anak na buhay?)**	Number of children alive **(Bilang ng anak na buhay)**	->105
105	**Women sometimes have pregnancies that do not result in a live born child. That is, a pregnancy can end early, in a miscarriage or the child can be born dead. Have you ever had a pregnancy that did not end in a live birth?**<br><br>May pagkakataon na ang pagbubuntis ay hindi nagbubunga sa pagluluwal ng isang buhay na bata. Maaring ang pagbubuntis ay mauwi sa pagkaagas	**1.** Yes **(Oo)**<br>2. No **(Hindi)**	1 ->106<br>2 -> 107

	o ipanganak ng patay. Nagkaroon na po ba kayo ng karanasan na ang inyong ipinagbubuntis ay hindi nailuwal ng buhay?		
106	**In all, how many pregnancies have you had that did not end in a live born child?**<br>**Sa kabuuan, ilang po sa inyong pagbubuntis ang hindi nagresulta sa isang buhay na sanggol?**<br><br>**(WRITE DOWN NUMBER BY SPONTANEOUS ABORTION AND INDUCED ABORTION SEPARATELY)**<br>**(ISULAT NG HIWALAY ANG BILANG NG PAGBUBUNTIS NA KUSANG NALAGLAG SA BILANG NG PAGBUBUNTIS NA SAPILITANG INILAGLAG.)**	**A. Number of pregnancy losses by nalaglag (spontaneous abortion)**<br>**A. Bilang ng pagbubuntis na kusang nalaglag.**<br><br>**B. Number of pregnancy losses by pinalaglag (induced abortion)**<br><br>**B. Bilang ng pagbubuntis na sapilitaing ipinilaglag.**	**->107**
107	Are you covered by any health insurance, either as member or dependent? (***Meron po ba kayong health insurance, bilang miyembro o dependent***.)?	1. Not covered (***Walang health insurance***)<br>2. Philhealth<br>3. Government Service Insurance System (GSIS)<br>4. Social Security System (SSS)<br>***5.*** Private insurance company/Health (maintenance organization /Pre-need insurance plan company (***Pribadong health insurance.)***<br>6. Other (Specify)**(*Iba pa… pakisabi..)***)	->201
201	REVIEW: Are you or your husband/partner currently doing something or using any method to delay or avoid getting pregnant?<br>***(Kayo po ba o ang inyong asawa/kapartner ay may ginagawa o may ginanagamit na pamamaraan para ipagpaliban o maiwasan na kayo ay magbuntis?)***	1. Yes **(Oo)**<br>2. No **(Hindi)**	1 ->201<br>2 -> 206
202	Which method are you currently using? (Ano pong pamamaraan o method ang inyong ginagamit?)<br><br>WRITE DOWN ALL MENTIONED.	1. Female sterilization **(Pagkapon sa babae)**<br>2. Male sterilization (**Pagkapon sa lalaki**)<br>3. IUD<br>4. Injectable (e.g.DMPA)<br>5. Implants<br>6. Patch<br>7. Pill<br>8. Condom<br>9. Female condom<br>10. Diaphragm<br>11. Foam/Jelly/Cream	->203

REPONDENT NUMBER:

Region	Province	City/Mun	Brgy	No

		12. Mucus/Billings/Ovulation<br>13. Basal body temperature<br>14. Symptothermal<br>15. Standard days method<br>16. LAM<br>17. Calendar/Rhythm/Periodic abstinence<br>18. Withdrawal<br>19. Other traditional method<br>20. Other modern method (specify)					
	LINE NUMBER	01	02	03	04	05	
203	Now I would like to ask you one by one about all methods you are using now. ***(Ngayon naman po ay isa-isa kong tatanungin ang tungkol sa lahat ng pamamaraan na ginagamit ninyo ngayon.)***<br><br>RECORD ALL METHODS BEING USED NOW, ONE METHOD PER ONE LINE NUMBER.<br>IF THERE ARE MORE THAN 5 METHODS, USE ADDITIONAL QUESTIONNAIRE. ***(ISULAT LAHAT NG PAMAMARAAN NA GINAGAMIT SA KASALUKUYAN, ISANG PAMAMARAAN SA BAWAT LINYA. KUNG MAY HIGIT SA LIMANG PAMAMARAAN, GUMAMIT NG ISA PANG QUESTIONNAIRE) (show card)***<br>1. Female sterilization ***(Pagkapon sa babae)***<br>2. Male sterilization ***(Pagkapon sa lalaki)***<br>3. IUD<br>4. Injectable (e.g.DMPA)<br>5. Implants<br>6. Patch<br>7. Pill<br>8. Condom<br>9. Female condom<br>10. Diaphragm<br>11. Form/Jelly/Cream<br>12. Mucus/Billings/Ovulation<br>13. Basal body temperature<br>14. Symptothermal<br>15. Standard days method<br>16. LAM<br>17. Calendar/Rhythm/Periodic abstinence<br>18. Withdrawal<br>19. Other traditional method<br>20. Other modern method (specify)						->204
204	Where did you obtain that method when you first started using it? ***(Nang mag-umpisa po kayo na gumamit ng _______ saan po kayo kumuha nito.)***						->205

REPONDENT NUMBER:

Region	Province	City/Mun	Brgy	No

	1. National hospital<br>2. Regional hospital/Public medical center<br>3. Provincial hospital<br>4. District hospital<br>5. Municipal hospital<br>6. Rural health unit (RHU)/urban health center(UHC)/Lying-in<br>7. Barangay health station (BHS)<br>8. Barangay supply/service point officer/BHW<br>9. Mobile clinic<br>10. Other (specify. Private facility is included here)						
205	What was the purpose of your going to the health facility on the day you first received the contraceptive method**? *(Ano po ang dahilan at kayo ay nagpunta sa health center noong una kayong nakatanggap/gumamit ng ________________*** 1. Prenatal care ***(Magkonsulta dahil buntis)***<br>***2.*** Giving birth, while a women is still in the facility (***Nanganak, habang nasa panganakan pa.)***<br>3. Health check after giving birth, after a woman left the facility ***(Nagkonsulta pagkatapos na manganak, nakaalis sa paanakan)***<br>**4.** Receiving vaccination or routine check up for child **(Para pabakunahan ang anak o regular na check up ng bata.)**<br>*5.* Seeking medical advice or treatment for sickness or injury of child ***(Nagkonsulta o ipinagamot ang maysakit o nasaktan ang bata.)***<br>*6.* Seeking medical advice or treatment for sickness or injury of herself ***(Nagkonsulta o nagpagamot dahil sa sya ay maysakit o nasaktan.)***<br>7. Adolescent clinic<br>8. Other (specify)						-> 203. Repeat until all methods were explained.<br><br>Then<br>->206
206	If you <u>are not</u> using any method to delay or avoid getting pregnant now, have you or your sexual partner done something or used a method to delay or avoid getting pregnant in the past*?* ***(Kung kayo po ay hindi gumagamit nang ano mang paraan para madelay ang pagbubuntis sa ngayon, kayo po ba o ang inyong kapartner ay may ginagawa o ginagamit na pamamaraan para madelay or hindi magbuntis noong nakaraang panahon?)***					1. Yes **(Oo)**<br>2. No **(Hindi)**	1->207<br>2-> 301

REPONDENT NUMBER:

Region	Province	City/Mun	Brgy	No

<table>
<tr><td></td><td colspan="6">If <u>you are</u> using a method to delay or avoid getting pregnant now, have you or your sexual partner ever used a different method to delay or avoid getting pregnant in the past? (Kung kayo ay gumagamit ng method o paraan para mdelay or huwag magbuntis sa ngayon, kayo ba o ang inyong kapartner ay gumamit ng ibang pamamaraan o method para madelay o hindi magbunti noong unang panahon?)</td><td></td><td></td></tr>
<tr><td>207</td><td>Which methods have you used in the past? (Alin-aling pong method o mga pamamaraan ang inyong ginamit noong nakaraang panahon?)

WRITE DOWN ALL MENTIONED. (ISULAT LAHAT NG PAMAMARAAN O METHOD.)</td><td colspan="6">1. Female sterilization (Pagkapon sa babae)
2. Male sterilization (Pagkapon sa lalaki)
3. IUD
4. Injectable (e.g.DMPA)
5. Implants
6. Patch
7. Pill
8. Condom
9. Female condom
10. Diaphragm
11. Foam/Jelly/Cream
12. Mucus/Billings/Ovulation
13. Basal body temperature
14. Symptothermal
15. Standard days method
16. LAM
17. Calendar/Rhythm/Periodic abstinence
18. Withdrawal
19. Other traditional method
20. Other modern method (specify)</td><td>-> 208</td></tr>
<tr><td></td><td>LINE NUMBER</td><td>01</td><td>02</td><td>03</td><td>04</td><td>05</td><td></td><td></td></tr>
<tr><td>208</td><td>Now I would like to ask you one by one about all methods you have used in the past. (Ngayon naman po ay isa-isa kong tatanungin ang tungkol sa lahat ng pamamaraan na ginagamit ninyo noong nakaraang panahon).

RECORD ALL METHODS, ONE METHOD PER ONE LINE NUMBER. (ISULAT LAHAT NG PAMAMARAAN NA GINAGAMIT NOONG NAKARAAN, ISANG PAMAMARAAN SA BAWAT LINYA.)

IF THERE ARE MORE THAN 5 METHODS, USE ADDITIONAL QUESTIONNAIRE. (KUNG MAY HIGIT SA LIMANG PAMAMARAAN, GUMAMIT NG ISA PANG QUESTIONNAIRE)

1. Female sterilization (Pagkapon sa babae)</td><td></td><td></td><td></td><td></td><td></td><td></td><td>-> 209</td></tr>
</table>

REPONDENT NUMBER:

Region	Province	City/Mun	Brgy	No

	**2.** Male sterilization **(Pagkapon sa lalaki)**<br>3. IUD<br>4. Injectable (e.g.DMPA)<br>5. Implants<br>6. Patch<br>7. Pill<br>8. Condom<br>9. Female condom<br>10. Diaphragm<br>11. Form/Jelly/Cream<br>12. Mucus/Billings/Ovulation<br>13. Basal body temperature<br>14. Symptothermal<br>15. Standard days method<br>16. LAM<br>17. Calendar/Rhythm/Periodic abstinence<br>18. Withdrawal<br>19. Other traditional method<br>20. Other modern method (specify)							
209	Where did you obtain the family planning method when you first started using it? ***(Nang mag-umpisa po kayo na gumamit ng _______ san po kayo kumuha nito.)***<br><br>1. National hospital<br>2. Regional hospital/Public medical center<br>3. Provincial hospital<br>4. District hospital<br>5. Municipal hospital<br>6. Rural health unit (RHU)/urban health center(UHC)/Lying-in<br>7. Barangay health station (BHS)<br>8. Barangay supply/service point officer/BHW<br>9. Mobile clinic<br>10. Other (specify. Private facility is included here.)							-> 210
210	Why did you visit the health facility where you first started using the family planning method? ***(Ano po ang dahilan at kayo ay nagpunta sa health center noong una kayong nakatanggap/gumamit ng _________________***<br><br>1. Reproductive Health Clinic, Mother, Child<br>2. Prenatal care (Magkonsulta dahil buntis)<br>3. Giving birth, while a women is still in the facility (***Nanganak, habang nasa panganakan pa.)***							-> 211

REPONDENT NUMBER:

Region	Province	City/Mun	Brgy	No

	4. Health check after giving birth, after a woman left the facility ***(Nagkonsulta pagkatapos na manganak, nakaalis sa paanakan)***<br>**5.** Receiving vaccination or routine check up for child **(Para pabakunahan ang anak o regular na check up ng bata.)**<br>*6.* Seeking medical advice or treatment for sickness or injury of **child** ***(Nagkonsulta o ipinagamot ang maysakit o nasaktan ang bata.)***<br>*7.* Seeking medical advice or treatment for sickness or injury of **herself** ***(Nagkonsulta o nagpagamot dahil sa sya ay maysakit o nasaktan.)***<br>8. Other (specify)							
211	Why did you stop using the family planning method that you used in the past? ***(Bakit po ninyo itinigil ang paggamit ng _________________________***<br><br>1. Side effects ***(May side effect/ibang epekto)***<br>***2.*** Method not available at the facility ***(Wala sa health clinic.)***<br>3. Concerns about risks of pregnancy ***(Agam-agam tungkol sa epekto ng panganganak)***<br>*4.* Could not afford to purchase ***(Hindi kayang bilihin.)***<br>***5.*** Health worker did not continue to provide the method. ***(Ang health worker ay itinigil na ang pagbibigay ng _____________.***<br>***6. Quality of care provided by the health facility or provider*** ***(Uri ng pag-aalaga ng pasilidad na pangkalusugan o tagapagalaga.)***<br>***7.*** Advice of friends, relatives, neighbors ***(Payo ng kaibigan, kamaganak, at kapitbahay)***<br>8. Husband/partner did not support **or allow to use** **(Ayaw ng asawa o partner o pinayagang gumamit)**<br>9. Wanted to get pregnant ***(Gustong magbuntis)***<br>10. Other (specify):___________							-> 212

REPONDENT NUMBER:

Region	Province	City/Mun	Brgy	No

	**Section 3. FP Concerns and Today's FP counseling**							
301	Do you have any health concerns about any type of family planning method? ***(Meron po ba kayong inalala na pangkalusugan sa pagamit ng ano mang uri ng pamamaraan ng pagpaplano ng pamilya?)***	1. Yes **(Oo)**<br>2. No **(Hindi)**						1 ->302<br>2 ->305
	LINE NUMBER	01	02	03	04	05	06	
302	What are your health concerns about family planning methods*?* ***(Ano-ano po ang inyong mga inaalalang ipekto sa kalusugan ng bawat pamamaraan?)***<br>Please tell me one by one. ***(Pakisabi po ninyo ang bawat isa.)***<br><br>USE ONE LINE NUMBER FOR ONE CONCERN. WRITE DOWN ALL MENTIONED CONCERNS. (**ISANG LINYA SA BAWAT ALALAHANIN. ISULAT ANG LAHAT NA ALALAHANIN.)**<br>IF THERE ARE MORE THAN 6 CONCERNS, USE ADDITIONAL QUESTIONNAIRE. *KUNG MAY HIGIT SA ANIM NA PAMAMARAAN, GUMAMIT NG ISA PANG QUESTIONNAIRE)*<br>*1.* Cause cancer in the uterus ***(Maging sanhi ng kanser sa matris)***<br>***2.*** Cause cysts in the uterus ***(Maging sanhi ng bukol sa matris)***<br>***3.*** Cause infection of the uterus ***(Maging sanhi ng inpeksyon sa matris)***<br>***4.*** Cause frequent bleeding ***(Maging sanhi ng malimit na pagdurugo)***<br>**5.** Cause thyroid problems **(Maging sanhi ng problema sa thyroid)**<br>**6.** Cause/worse asthma (***Maging sanhi para magkaroon* o mas lumlala ang asthma)**<br>7. Cause/worsen lots of veins ***(Maging sanhi o mas lumala ang mga ugat??)***<br>8. Cause dry skin, skin disease (***Maging sanhi para matuyo ang balat o sakit sa balat***.)<br>***9.*** Cause edema (***Maging sanhi ng pamamanas)***<br>***10.***Cause weight gain (***Maging sanhi para tumaba.)***<br>**11.**Cause weight loss ***(Maging sanhi para pumayat.)***<br>***12.***Cause bloated stomach ***(Maging sanhi ng mamaga ang tyan)***							-> 303

13. Cause headache ***(Maging sanhi para sumakit and ulo.)***

14. Cause irritability ***(Maging sanhi ng pagiging bugnutin.)***

15. Increase libido/turn into a maniac ***(Maging dahilan para tumaas ang libog/maging maniac.)***

16. Cause loss/reduce of libido **(Maging dahilan para mabawasan/mawala ang libog)**

17. Cause loss/reduce of sexual satisfaction **(Maging dahilan para mabawasan/mawala ang sekswal na kasiyahan.)**

18. One will not have children anymore **(Maging dahilan para hindi na magkaanak.)**

19. Not fully effective, woman could still get pregnant **(Hindi lubos na epektibo, ang babae ay pwede pa ring mabuntis.)**

20. When it does not work, the baby is born with abnormalities **(Kapag ito ay sumablay, ang batang ipapanganak ay may abnormalities)**

21. Results in mortal sin because it is against church teachings **(Ang paggamit ay isang mortal na kasalanan dahil ito ay laban sa mga turo ng simbahan.)**

IUD/Implants

22. Melt or move around inside the body and doctors will not be able to find. ***(Natutunaw o gumagala sa loob ng katawan at hindi na makikita ng doktor.)***

23. Washed away/pushed out of body. *(**Lumalabas ng kusa sa katawan**.)*

24. Painful to insert (***Masakit pag inilalagay.)***

IUD

25. Itchy on the vagina. **(Makati sa ari.)**

26. Entangled around the man's penis **(Pumupulupot sa titi.)**

27. Messy when inserted **(Makalat pag inilalagay.)**

Male sterilization

REPONDENT NUMBER:

Region	Province	City/Mun	Brgy	No

	28.Part of the man's testicles are cut off ***(May parte ng bayag na napuputol.)***<br>***29.***It hurts the testicles ***(Nakakasakit sa bayag.)***<br>**30.**The man loses his manhood ("kapon") ***(Nakakawala ng pagkalalaki.)***<br><br>31. Others (specify) **(Iba, pakibanggit)**							
303	About which family planning methods do you have concerns***? (Alin pong pagpaplano ng pamilya ang meron kayong pag-aalala o agam-agam?)***<br><br>REPEAT EACH CONCERN IN TURN. FOR EACH CONCERN, WRITE DOWN ALL METHODS CAUSING THAT CONCERN. **(ULITIN ANG BAWAT ALALAHANIN. SA BAWAT ALALAHANIN, ISULAT LAHAT NG PARAAN NG PAGPAPLANO NG PAMILYA.)**<br><br>1. Female sterilization (Pagkapon sa babae)<br>2. Male sterilization (Pagkapon sa lalaki)<br>3. IUD<br>4. Injectable<br>5. Implants<br>6. Patch<br>7. Pill<br>8. Other modern method (specify)<br>9. Other method (specify)							-> 304
304	Who told you or how did you find about your concerns about family planning methods? ***(Sino po ang nagsabi sa inyo o paano kayo nagkaroon ng pag-aalala o agam-agam tungkol sa pamaraan ng pagpaplano ng pamilya.)***<br><br>REPEAT EACH CONCERN IN TURN. FOR EACH WRITE DOWN ALL SOUCES OF INFORMATION. **(ULITIN ANG BAWAT ALALAHANIN. SA BAWAT PAMAMARAAN, ISULAT ANG BAWAT PINAGMULAN NG INPORMASYON)**<br>1. Health staff<br>2. BHW or health volunteers<br>3. Husband or partner **did not want to use** ***(Ayaw gumamit ng asawa o partner)***<br>*4.* Friend, neighbours, relatives ***(Kaibigan, kapitbahay, kamaganak)***							-> 305

	5. Church ***(Simbahan)***<br>6. Radio ***(Radyo)***<br>7. Television ***(Telebisyon)***<br>*8.* Newspaper or magazine ***(Dyaryo o magasin)***<br>9. Online or internet<br>10. Others (specify)								

305	Today, did any staff member at the health facility speak to you about family planning methods? ***(Ngayon po, meron po bang tauhan ng health center na kinausap kayo tungkol sa pagpaplano ng pamilya?)***	1. Yes (Oo)<br>2. No (Hindi)	1 ->306<br>2 ->401
306	Did the health worker ask you about your concerns**?** ***(Tinanong po ba kayo ng health worker tungkol sa inyong mga inaalala o agam-agam?)***	1. Yes (Oo)<br>2. No (Hindi)	1 ->307<br>2 ->409
307	Do you feel the health worker understands your concerns? (Sa palagay mo naiintindihan ng health worker ang inyong mga alalahanin?)	1. Yes (Oo)<br>2. No (Hindi)	->308
308	Did the health worker help you to find solutions to your concerns? (Natulungan po ba kayo ng health worker na humanap ng solusyon sa inyong katanungan?)	1. Yes (Oo)<br>2. No (Hindi)	->309
309	Did the health worker offer you information how different family planning methods work? (Binigyan po ba kayo ng impormasyon kung paano ginagamit ang ibat-ibang pamamaraan sa pagpaplano ng pamilya?)	1. Yes (Oo)<br>2. No (Hindi)	1 ->310<br>2 ->312
310	Which methods did health worker mention today? (***Aling paraan ng pagpaplano ng pamilya ang nabanggit ng health worker?)***	1. Female sterilization (Pagkapon sa babae)<br>2. Male sterilization (Pagkapon sa lalaki)<br>3. IUD<br>4. Injectable (e.g.DMPA)<br>5. Implants<br>6. Patch<br>7. Pill<br>8. Condom<br>9. Female condom<br>10. Diaphragm<br>11. Foam/Jelly/Cream<br>12. Mucus/Billings/Ovulation<br>13. Basal body temperature<br>14. Symptothermal	->311

REPONDENT NUMBER:

Region	Province	City/Mun	Brgy	No

		15. Standard days method<br>16. LAM<br>17. Calendar/Rhythm/Periodic abstinence<br>18. Withdrawal<br>19. Other traditional method<br>20. Other modern method (specify)	
311	Did the health worker tell you about side-effects or problems you might have with any methods of family planning? ***(Nabanggit ba ng health worker ang mga ibang epekto o problema na dala ng paraan ng pagpaplano ng pamilya?)***	1. Yes **(Oo)**<br>2. No **(Hindi)**	-> 312
312	Did the health worker offer you information how your family planning method works? *(Nagbigay ba ng impormasyon kung ano o paano ginanagamit ang paraan ng pagpaplano ng pamilya?)*	1. Yes (**Oo**)<br>2. No (**Hindi**)<br>3. N/A (not using a method now)	1 -> 313<br>2-> 313<br>3-> 315
313	Did the health worker explain about the side effects of your current method? ***(Ipinaliwanag ba ng health worker ang ibang epekto ng inyong ginagamit sa kasalukuyan na paraan ng pagpaplano ng pamilya.)***	1. Yes (**Oo**)<br>2. No (**Hindi**)	-> 314
314	Did the health worker ask you to describe how you use your current method? ***(Tinanong po ba kayo ng health worker kung paano ginangamit ang kasalukuyang pamamaraan ng pagplano ng pamilya?.)***	1. Yes (**Oo**)<br>2. No (**Hindi**)	-> 401
315	After receiving FP counselling will you begin using a family planning method today? **(Pagkatapos po kayong payuhan tungkol sa pagpaplano ng pamilya, gagamitin ninyo po ba ito ngayon?)**	1. Yes (**Oo**)<br>2. No (**Hindi**)	1 -> 317<br>2 -> 316
316	After receiving FP counselling will you begin using, do you think you will use a contraceptive method anytime in the future? ***(Pagkatapos po kayong payuhan tungkol sa pagpaplano ng pamilya, kayo po ba ay gagamit ng paraan ng pagpaplano ng pamilya ano mang oras sa darating na panahon?)***	1. Yes (**Oo**)<br>2. No (**Hindi**)	1 -> 317<br>2 -> 316
317	Which contraceptive method would you prefer to use? ***(Alin pong pamamaraan ng pagpaplano ng pamilya ang inyong gustong gamitin?)***	1. Female sterilization (**Pagkapon sa babae)**	-> 401

REPONDENT NUMBER:

Region	Province	City/Mun	Brgy	No

		2. Male sterilization (**Pagkapon sa lalaki)**<br>3. IUD<br>4. Injectable (e.g.DMPA)<br>5. Implants<br>6. Patch<br>7. Pill<br>8. Condom<br>9. Female condom<br>10. Diaphragm<br>11. Foam/Jelly/Cream<br>12. Mucus/Billings/Ovulation<br>13. Basal body temperature<br>14. Symptothermal<br>15. Standard days method<br>16. LAM<br>17. Calendar/Rhythm/Periodic abstinence<br>18. Withdrawal<br>19. Other traditional method<br>20. Other modern method (specify)	

	Section 4. Past Health facility visit and FP counseling<br>**Do not count today's visit.**						
401	Not including today, in the last 12 months, have you visited a health facility for care for yourself or your children for any purpose? ***(Kung hindi po natin isasama ang pagpunta ninyo ngayon, kayo po ba ay nakapunta sa isang health facility/hospital para sa pangangalaga ng inyong kalusugan o para sa inyong mga anak sa ano mang dahilan?***	1. Yes **(Oo)**<br>2. No **(Hindi)**			1 -> 402<br>**2 -> END OF INTERVIEW**		
	LINE NUMBER	01	02	03	04	05	06
402	Now I would like to record all your facility visits for last 12 months. Start with the latest visit you had.<br>Why did you visit a health facility***? (Ngayon po ay itatanong ko ang lahat ng pagpunta ninyo sa health clinic/hospital simula Enero hanggang Disyembre 2016.) Bakit po kayo pumunta sa health clinic/hospital?***<br><br>AFTER WRITING THE FIRST VISIT IN LINE NUMBER 01, ASK Q403-410 FOR THAT VISIT. THEN ASK THE 2nd LATEST VISIT TO WRITE IN 402 LINE NUMBER 02, THEN ASK Q 403 AND Q404. (**PAGKATAPOS ISULAT ANG HULING PAGPUNTA SA HEALTH CLINIC SA UNANG LINYA, ITANONG ANG**						

	**IKALAWA SA PINAKAHULING PAGPUNTA SA HEALTH CLINIC AT ISULAT SA Q402 SA BILANG 02, PAGKATAPOS ITANONG ANG Q403 AT 404).**<br>REPEAT FOR ALL HEALTH FACILITY VISITS FOR LAST 12 MONTHS. **(ULITIN PARA SA LAHAT NG PAGPUNTA SA HEALTH CLINIC SIMULA ENERO HANGGANG ENERO 2016) IF THERE ARE MORE THAN 6, USE AN ADDITIONAL QUESTIONNAIRE. (KUNG MAHIGIT SA ANIM, GUMAMIT NG ISA PANG QUESTIONNAIRE.)**<br><br>1. Prenatal care ***(Magkonsulta dahil buntis)***<br>***2.*** Giving birth, while a women is still in the facility (***Nanganak, habang nasa panganakan pa.)***<br>3. Health check after giving birth, after a woman left the facility ***(Nagkonsulta pagkatapos na manganak, nakaalis sa paanakan)***<br>**4.** Receiving vaccination or routine check up for child **(Para pabakunahan ang anak o regular na check up ng bata.)**<br>*5.* Seeking medical advice or treatment for sickness or injury of **child *(Nagkonsulta o ipinagamot ang maysakit o nasaktan ang bata.)***<br>*6.* Seeking medical advice or treatment for sickness or injury of **herself *(Nagkonsulta o nagpagamot dahil sa sya ay maysakit o nasaktan.)***<br>7. Adolescent clinic<br>8. Other (specify)						
403	Where did you visit? ***(Saan po kayo nagpuntang health facility?)***<br><br>1. National hospital<br>2. Regional hospital/Public medical center<br>3. Provincial hospital<br>4. District hospital<br>5. Municipal hospital<br>6. Rural health unit (RHU)/urban health center(UHC)/Lying-in<br>7. Barangay health station (BHS)<br>8. Barangay supply/service point officer/BHW<br>9. Mobile clinic<br>10.Other (specify. Private facility is included here.)						
404	At that visit, were you or your sexual partner already using any method to delay or avoid getting pregnant? (***Noong pumunta po kayo sa health clinic/hospital, kayo po ba o ang inyong partner ay mayroon nang ginanamit na pamaraan para madelay or para maiwasang magbuntis)***						

REPONDENT NUMBER:

Region	Province	City/Mun	Brgy	No

	1. Yes (**Oo**)<br>2. No (**Hindi**)						
405	Which method(s) were you using? ***(Alin pong pamamaraan ang inyong ginangamit?)***<br><br>WRITE DOWN ALL MENTIONED **(ISULAT LAHAT NG PAMAMARAAN)**<br><br>1. Female sterilization (Pagkapon sa babae)<br>2. Male sterilization (Pagkapon sa lalaki)<br>3. IUD<br>4. Injectable (e.g.DMPA)<br>5. Implants<br>6. Patch<br>7. Pill<br>8. Condom<br>9. Female condom<br>10. Diaphragm<br>11. Foam/Jelly/Cream<br>12. Mucus/Billings/Ovulation<br>13. Basal body temperature<br>14. Symptothermal<br>15. Standard days method<br>16. LAM<br>17. Calendar/Rhythm/Periodic abstinence<br>18. Withdrawal<br>19. Other traditional method<br>20. Other modern method (specify)						
406	At that visit, did any staff member at the health facility speak to you about family planning methods? ***(Sa inyo pong pagpunta sa health clinic/hospital, meron po bang health staff na kumausap sa inyo tungkol sa paraan ng pagpaplano ng pamilya.)***<br><br>**1.** Yes (**Oo)**<br>2. No **(Hindi**)						
407	After that visit, did you start using any FP method or change from your previous method to a new method? ***(Pagkatapos po ng inyong pagpunta sa clinic, nagpalit po ba kayo ng paraan sa pagpaplano ng pamilya?)***<br><br>1. Yes (**Oo)**<br>2. No (**Hindi**)						
408	If you did not start a new method or change from your previous method, why? ***(Kung hindi nagpalit, bakit o anong dahilan?)***						

REPONDENT NUMBER:

Region	Province	City/Mun	Brgy	No

	1. No need (***Hindi kailangan.)***<br>***2.*** Possible side effects of new method ***(Posibleng epekto ng bagong pamamaraan.)***<br>***3.*** New method not available at the facility ***(Ang bagong pamamaraan ay wala sa health unit.)***<br>4. Concerns about risk of pregnancy with new method ***(Nag-aalala tungkol sa panganib sa pagbubuntis ng bagong pamamaraan.)***<br>***5.*** Not enough information ***(Walang sapat na impormasyon.)***<br>6. Could not afford to purchase **(Hindi kayang bilihin.)**<br>***7.*** Advice of friends, relatives, neighbours not to start or change (***Payo ng mga kaibigan, kamaganak, kapitbahay na huwag gumamit o magpalit ng paraan ng pagpaplano ng pamilya.)***<br>***8.*** Husband/partner did not support ***(Ayaw ng asawa/partner)***<br>9. Other (specify):___________						
409	Which FP method did you start using after that visit or which new method did you change to? ***(Pagkatapos po ng inyong pagpunta sa clinic, anong paraan ng pagpaplano ng pamilya ang inyo nang ginamit?)***<br><br>1. Female sterilization (Pagkapon sa babae)<br>2. Male sterilization (Pagkapon sa lalaki)<br>3. IUD<br>4. Injectable (e.g.DMPA)<br>5. Implants<br>6. Patch<br>7. Pill<br>8. Condom<br>9. Female condom<br>10. Diaphragm<br>11. Foam/Jelly/Cream<br>12. Mucus/Billings/Ovulation<br>13. Basal body temperature<br>14. Symptothermal<br>15. Standard days method<br>16. LAM<br>17. Calendar/Rhythm/Periodic abstinence<br>18. Withdrawal<br>19. Other traditional method<br>20. Other modern method (specify)						

END OF THE INTERVIEW
