# Supplementary material for: Opportunities lost: Barriers to increasing the use of effective contraception in the Philippines
Source: PLoS One. 2019 Jul 25;14(7):e0218187. doi: 10.1371/journal.pone.0218187 (PMC6657820; doi:10.1371/journal.pone.0218187)
Supplement: S8 Questionnaire — (PDF) [file pone.0218187.s008.pdf]

COVER PAGE

FORM1. Iinterview women of reproductive age who are not currently pregnant or within 6 weeks of delivery, and desire delaying or limiting childbearing

Sequence number: _____________

[Fill one number for each woman contacted in the order they were contacted at the health facility; if done over several days, continue unique sequence numbers]

Identification of interview place		
Region		
Province		
CITY/MUNICIPALITY		
BARANGAY		
Health facility name		
Home address (for home visit only)		
Latitude and longitude<br>(Use the coordinate of GPS in a mobile phone)		
Interview Record		
Date of interview		
Interviewer's name		
Health facility level where interview took place	1. National hospital<br>2. Regional hospital/Public medical center<br>3. Provincial hospital<br>4. District hospital<br>5. Municipal hospital<br>6. Rural health unit (RHU)/urban health center(UHC)/Lying-in<br>7. Barangay health station (BHS)<br>8. Barangay supply/service point officer/BHW<br>9. Mobile clinic<br>10. Other (specify)	
Clinic where interview took place (for large hospitals)	1. Reproductive health clinic for postnatal care<br>2. Reproductive health clinic not related to postnatal care<br>3. Receiving vaccination or routine check-up for child<br>4. Seeking medical advice or treatment for sickness or injury of child<br>5. Seeking medical advice or treatment for sickness or injury of **herself**<br>6. Other (specify)	

Sequence Number: ___________________
[Write the same sequence number from Cover Page]

Instructions:
Read the information sheet. Answer questions. If the woman agrees to participate give the certificate of consent for her to sign. Then start the Screening Form.

Screening Form

State: "We would like to start by asking a few questions that determine if you are eligible for the survey."
"In kami ini mabayah mangasubu maniyu tiyu kaniyo bang in kamo manjari para ha survey namo"

001	How old were you on your last birthday?<br>"Pila tahun na kaw amun last birth day mo?"	Age in completed years		18-49 years ->002<br>Other -> 009
002	Are you pregnant now?<br>"Burus kaw bihaun?"	1. Yes "Huun,Oo"<br>2. No "Bukon"<br>3. Unsure "Di ko kaingatan"		1 ->009<br>2 ->003<br>3 ->003
003	What is the name of your last baby?<br>"Uno in ngan sin kamanghuran anak mo?"<br>Record name	1. Name: _____________<br>2. No previous baby		1 ->004<br>2 ->006
004	In what month and year was NAME born?<br>"Uno bulan iban tahun sya piyanganak?"<br>(probe: when is his or her birthday)	Month: __ __<br><br>Year: __ __ __ __		Age $\geq$6 wks ->005<br>Age<6 wks ->009
005	Has your menstrual period returned since the birth of NAME?<br>"Nagbalik na ba in pagdugu mo amun pagubus sin pag anak mo?"	1. Yes<br>2. No		1 ->006<br>2 ->006
006	Now I have some questions about the future. Would you like to have (a/another) child, or would you prefer not to have any (more) children?<br>"Bihaun mangasubu ako. Mabayah pa kaw manganak magbalik atawa dih na?"	1. Have (a/another) child "Magkaanak magbalik"<br>2. No more/none "Dih na"<br>3. Cannot get pregnant "Dih na maburus"<br>4. Undecided / don't know "Dih ku kaingatan"		1 -> 007<br>2 ->008<br>3 ->009<br>4 ->009
007	Do you want (a/another) child soon?<br>"Mabayah pa kaw magka anak magbalik ha susungan?"	1. Yes "Huun,Oo"<br>2. No, want to wait "Dih muna"<br>3. Don't know "Inday ku"		1 ->009<br>2 ->008<br>3- >009
008	Are you or your husband/partner currently doing something or using any method to delay or avoid getting pregnant?<br>"Awun iyuusal nyo iban bana mo or hinang nyo para kaw di maburus magtuy"	1. Yes<br>2. No		1 -> 101<br>2 -> 101<br>To achieve a total of 5 users and non-users (hospitals) and 3 users and 3 non-users (health centres).
009	Thank the woman, indicate ineligibility for the survey and stop the interview. Enter this woman into "number of women contacted". Then find another woman to interview.			

FORM1. Interview of women of reproductive age who are not currently pregnant or within 6 weeks of delivery, and desire delaying or limiting childbearing

Sequence Number:
[Write the same sequence number from Cover Page]

NO.	Section 1. Respondent background			
101	In (month of interview) 2016, did you live in a city, in a town proper/ poblacion, in the barrio or rural area, or abroad?<br>"Ha tahun 2016 nakabutang kaw ha city proper, barangay atawa abroad?"	1. City<br>2. TOWN PROPERIPOBLACION<br>3. BARRIO/RURAL AREA<br>4. ABROAD<br>5. DON'T KNOW		->102
102	What is your marital status now?<br>"In ikaw yan budjang, awun na bana (natiyaun na), nagbugit iban bana?	1. Never married or never lived with a man "budjang"<br>2. Currently married "awun bana (natiyaun na)"<br>3. Currently living with a man "not applicable for Islam"<br>4. Divorced/separated/widow and not currently living with a man "nagbugit iban bana"		-> 103
103	What is your highest level of education attended, whether or not that level was completed?<br>"Uno in natalus mo ha iskul?"	1. No education "uway naka iskul"<br>2. Elementary<br>3. High school<br>4. College<br>5. Post-graduate		->104
104	How many children do you have who are still alive?<br>"Pila in anak mo bihaun?"	Number of children alive		->106
105	Did you or someone else do anything to end any of your past pregnancies?<br>"Bakas awun nahinang mo para di matuy in pagburus?"	1. Yes<br>2. No		1 ->107<br>2 -> 108
106	How many pregnancies did you or someone else do anything to end?<br>"Nakapila nahinang?"	Number of induced abortion		->108
107	Are you covered by any health insurance, either as member or dependent?<br>"Awun health insurance nyo?"	1. Not covered<br>2. Philhealth<br>3. Government Service Insurance System<br>4. Social Security System<br>5. Private insurance company/Health maintenance organization /Pre-need insurance plan company<br>6. Other (Specify)		->201

NO.	Section 2. Current use of FP			
201	REVIEW: Are you or your husband/partner currently doing something or using any method to delay or avoid getting pregnant?	3. Yes<br>4. No		1 -> 202<br>2 -> 206

	“Awun iyuusal nyo iban bana mo or hinang nyo para kaw di maburus magtuy”						
202	Which method are you currently using?<br>“Uno in mga hinang nyo?”<br>WRITE DOWN ALL MENTIONED.	1. Female sterilization<br>2. Male sterilization<br>3. IUD<br>4. Injectable (e.g.DMPA)<br>5. Implants<br>6. Patch<br>7. Pill<br>8. Condom<br>9. Female condom<br>10. Diaphragm<br>11. Form/Jelly/Cream<br>12. Mucus/Billings/Ovulation<br>13. Basal body temperature<br>14. Symptothermal<br>15. Standard days method<br>16. LAM<br>17. Calendar/Rhythm/Periodic abstinence<br>18. Withdrawal<br>19. Other traditional method<br>20. Other modern method (specify)					-> 203
	LINE NUMBER	01	02	03	04	05	
203	Now I would like to ask you one by one about all methods you are using now.<br>“Bihaun mabyah ako mangasubu knyo pasal ha mga method nyo”<br><br>RECORD ALL METHODS BEING USED NOW, ONE METHOD PER ONE LINE NUMBER.<br>IF THERE ARE MORE THAN 5 METHODS, USE ADDITIONAL QUESTIONNAIRE.<br><br>1. Female sterilization<br>2. Male sterilization<br>3. IUD<br>4. Injectable (e.g.DMPA)<br>5. Implants<br>6. Patch<br>7. Pill<br>8. Condom<br>9. Female condom<br>10. Diaphragm<br>11. Form/Jelly/Cream<br>12. Mucus/Billings/Ovulation<br>13. Basal body temperature<br>14. Symptothermal<br>15. Standard days method<br>16. LAM<br>17. Calendar/Rhythm/Periodic abstinence<br>18. Withdrawal<br>19. Other traditional method<br>20. Other modern method (specify)						->204

204	Where did you obtain that method when you first started using it?<br><br>"Hawnu mo nakawa in mga method ini?"<br><br>1. National hospital<br>2. Regional hospital/Public medical center<br>3. Provincial hospital<br>4. District hospital<br>5. Municipal hospital<br>6. Rural health unit (RHU)/urban health center(UHC)/Lying-in<br>7. Barangay health station (BHS)<br>8. Barangay supply/service point officer/BHW<br>9. Mobile clinic<br>10. Other (specify. Private facility is included here)						-> 205
205	What was the purpose of your going to the health facility on the day you first received the contraceptive method?<br>"Uno in natalih mo amun miyadtu kaw pa health facility ubus dinihilan kaw contraceptive method?"<br><br>1. Prenatal care<br>2. Giving birth, while a women is still in the facility<br>3. Reproductive health outpatient clinic for postnatal care<br>4. Reproductive health clinic not related to postnatal care<br>5. Receiving vaccination or routine check up for child<br>6. Seeking medical advice or treatment for sickness or injury of **child**<br>7. Seeking medical advice or treatment for sickness or injury of **herself**<br>8. Adolescent clinic<br>9. Other (specify)						-> 203. Repeat until all methods were explained.<br><br>Then ->206
206	If you <u>are not</u> using any method to delay or avoid getting pregnant now, have you or your sexual partner done something or used a method to delay or avoid getting pregnant in the past?<br>"Bang kaw y nagusal protection para dih maburus bihaun. Nakausal kamo iban banah mo sin tagna?"	1. Yes<br>2. No					1->207<br>2-> 301

<table>
<tr><td></td><td></td><td colspan="3"></td><td colspan="2"></td><td></td></tr>
<tr><td>207</td><td>Which methods have you used in the past?
“Uno method in nahinang nyo tagna?”

WRITE DOWN ALL MENTIONED.</td><td colspan="3">1. Female sterilization
2. Male sterilization
3. IUD
4. Injectable (e.g.DMPA)
5. Implants
6. Patch
7. Pill
8. Condom
9. Female condom
10. Diaphragm
11. Form/Jelly/Cream
12. Mucus/Billings/Ovulation
13. Basal body temperature
14. Symptothermal
15. Standard days method
16. LAM
17. Calendar/Rhythm/Periodic abstinence
18. Withdrawal
19. Other traditional method
20. Other modern method (specify)</td><td colspan="2"></td><td>-> 208</td></tr>
<tr><td></td><td>LINE NUMBER</td><td>01</td><td>02</td><td>03</td><td>04</td><td>05</td><td></td></tr>
<tr><td>208</td><td>Now I would like to ask you one by one about all methods you have used in the past
“Bihaun mabyah ako mangasubu knyo pasal ha mga method nyo”

RECORD ALL METHODS, ONE METHOD PER ONE LINE NUMBER.
IF THERE ARE MORE THAN 5 METHODS, USE ADDITIONAL QUESTIONNAIRE.

1. Female sterilization
2. Male sterilization
3. IUD
4. Injectable (e.g.DMPA)
5. Implants
6. Patch
7. Pill
8. Condom
9. Female condom
10. Diaphragm
11. Form/Jelly/Cream
12. Mucus/Billings/Ovulation
13. Basal body temperature
14. Symptothermal
15. Standard days method
16. LAM
17. Calendar/Rhythm/Periodic abstinence
18. Withdrawal
19. Other traditional method
20. Other modern method (specify)</td><td></td><td></td><td></td><td></td><td></td><td>->209</td></tr>
</table>

209	Where did you obtain the family planning method when you first started using it?<br>"Hawnu mo nakawa tagna in family planning method"<br><br>1. National hospital<br>2. Regional hospital/Public medical center<br>3. Provincial hospital<br>4. District hospital<br>5. Municipal hospital<br>6. Rural health unit (RHU)/urban health center(UHC)/Lying-in<br>7. Barangay health station (BHS)<br>8. Barangay supply/service point officer/BHW<br>9. Mobile clinic<br>10. Other (specify. Private facility is included here.)						->210
210	Why did you visit the health facility where you first started using the family planning method?<br>"Maytah kaw miyadtu pa health facility amun hawnu mo nakawa in family planning method?"<br>1. Prenatal care<br>2. Giving birth, while still in the facility "Nag anak"<br>3. Reproductive health outpatient clinic for postnatal care<br>4. Reproductive health clinic not related to postnatal care<br>5. Receiving vaccinations or routine check-ups for a child "nagpa vaccine"<br>6. Seeking medical advice or treatment for sickness or injury of a **child** "nagpa ubat sin anak awun sakit"<br>7. Seeking medical advice or treatment for sickness or injury of **herself** "nagpa ubat aku"<br>8. Adolescent clinic<br>9. Other (specify)						-> 211
211	Why did you stop using the family planning method that you used in the past?<br>"Maytah kaw humali ha family planning method na nausal mo tagna?<br>1. Side effects<br>2. Method not available at the facility						->212

	3. Concerns about risks of pregnancy<br>4. Could not afford to purchase<br>5. Health worker did not continue to provide the method<br>6. Advice of friends, relatives, neighbors<br>7. Husband/partner did not support<br>8. Wanted to get pregnant<br>9. Other (specify):___________						

	Section 3. FP Concerns and Today's FP counseling							
301	Do you have any health concerns about any type of family planning method?<br>"Awun mga pangasubu mo pasal ha family planning method?"	1. Yes<br>2. No						1 ->302<br>2 ->305
	LINE NUMBER	01	02	03	04	05	06	
302	What are your health concerns about family planning methods? Please tell me one by one.<br>"Uno in mga pangasubo mo pasal ha family planning methods?" Iyana kako.<br>USE ONE LINE NUMBER FOR ONE CONCERN. WRITE DOWN ALL MENTIONED CONCERNS.<br>IF THERE ARE MORE THAN 6 CONCERNS, USE ADDITIONAL QUESTIONNAIRE.<br><br>1. Cause cancer in the uterus<br>2. Cause cysts in the uterus<br>3. Cause infection of the uterus<br>4. Cause frequent bleeding<br>5. Cause thyroid problems<br>6. Cause/worse asthma<br>7. Cause/worse lots of veins<br>8. Cause dry skin, skin disease<br>9. Cause edema<br>10. Cause weight gain<br>11. Cause weight loss<br>12. Cause bloated stomach<br>13. Cause headache<br>14. Cause irritability<br>15. Increase libido/turn into a maniac<br>16. Cause loss/reduce of libido<br>17. Cause loss/reduce of sexual satisfaction<br>18. One will not have children anymore<br>19. Not fully effective, woman could still get pregnant<br>20. When it does not work, the baby is born with abnormalities<br>21. Results in mortal sin because it is against church teachings<br><br>**IUD/Implants**<br>22. Melt or move around inside the body and doctors will not be able to find<br>23. Washed away/pushed out of body							-> 303

	24.Painful to insert<br><br>**IUD**<br>25.Itchy on the vagina<br>26.Entangled around the man's penis<br>27.Messy when inserted<br><br>**Male sterilization**<br>28.Part of the man's testicles are cut off<br>29.It hurts the testicles<br>30.The man loses his manhood ("kapon")<br><br>31. Others (specify)							
303	About which family planning methods do you have concerns? "Uno ha mga family planning in pangasubo mo?"<br>REPEAT EACH CONCERN IN TURN. FOR EACH CONCERN, WRITE DOWN ALL METHODS CAUSING THAT CONCERN.<br><br>1. Female sterilization<br>2. Male sterilization<br>3. IUD<br>4. Injectable<br>5. Implants<br>6. Patch<br>7. Pill<br>8. Other modern method (specify)<br>9. Other method (specify)							-> 304
304	Who told you or how did you find about your concerns about family planning methods?<br>"Siyu nagbaytah kaymo atawa biyadin kiyaingatan mo pasal ha mga family planning methods?"<br>REPEAT EACH CONCERN IN TURN. FOR EACH WRITE DOWN ALL SOUCES OF INFORMATION.<br><br>1. Health staff<br>2. BHW or health volunteers<br>3. Husband or partner<br>4. Friend, neighbours, relatives<br>5. Church<br>6. Radio<br>7. Television<br>8. Newspaper or magazine<br>9. Online or internet<br>10. Others (specify)							-> 305
305	Today, did any staff member at the health facility speak to you about family planning methods?<br>Bihaun, awun ba mga taga healt center dima kaniyo nagbissara	1. Yes<br>2. No						1 ->306<br>2 ->401

	pasal ha family planning method?"			
306	Did the health worker ask you about your concerns?<br>"Iyasubo kaw sin tau ha health worker pasal ha mga problema (concern) nyo?"	1. Yes<br>2. No		1 ->307<br>2 -> 309
307	Do you feel the health worker understands your concerns?<br>"Kiyahatihan da kaw sin health worker pasal sin problema (concern) mo?"	1. Yes<br>2. No		->308
308	Did the health worker help you to find solutions to your concerns?<br>Timabang ba in health worker kymo pasal ha problema (concern)?"	1. Yes<br>2. No		->309
309	Did the health worker offer you information how different family planning methods work?<br>"Nagdihil ba in health worker bang uno-uno in karayawan sin mga family planning methods?"	1. Yes<br>2. No		1 ->310<br>2 ->312
310	Which methods did health worker mention today?<br>"Uno method in biyaytah knyo adlaw ini?"	1. Female sterilization<br>2. Male sterilization<br>3. IUD<br>4. Injectable (e.g.DMPA)<br>5. Implants<br>6. Patch<br>7. Pill<br>8. Condom<br>9. Female condom<br>10. Diaphragm<br>11. Form/Jelly/Cream<br>12. Mucus/Billings/Ovulation<br>13. Basal body temperature<br>14. Symptothermal<br>15. Standard days method<br>16. LAM<br>17. Calendar/Rhythm/Periodic abstinence<br>18. Withdrawal<br>19. Other traditional method<br>20. Other modern method (specify)		- >311
311	Did the health worker tell you about side-effects or problems you might have with any methods of family planning?<br>"Kiya baytaan kamo sin health worker pasal sin problema sin methods of family planning?"	1. Yes<br>2. No		-> 312
312	Did the health worker offer you information how your family planning method works?<br>"Kiyabaytaan ba kaw sin health worker bang uno in mahinang sin family planning methods?"	1. Yes<br>2. No<br>3. N/A (not using a method now)		1 -> 313<br>2-> 313<br>3-> 315

313	Did the health worker explain about the side effects of your current method?<br>"Biyaytaan kaw sin healt worker bng uno in side effect sin iyuusal mo method bihaun?"	1. Yes<br>2. No		-> 314
314	Did the health worker ask you to describe how you use your current method?<br>"Iyasubo kaw sin health worker bng bihadiin usalun in method iyuusal mo?"	1. Yes<br>2. No		-> 401
315	After receiving FP counselling will you begin using a family planning method today?<br>"Bihaun kaingatan mo na in pasal ha family planning, usalun mo na bihaun in family planning?"	1. Yes<br>2. No		1 -> 317<br>2 -> 316
316	After receiving FP counselling will you begin using, do you think you will use a contraceptive method anytime in the future?<br>"Bihaun kaingatan mo na in pasal ha family planning atawa ha susungan?"	1. Yes<br>2. No		1-> 317<br>2-> 401
317	Which contraceptive method would you prefer to use?<br>"Uno contraceptive method in kabayaan mo usalun?"	1. Female sterilization<br>2. Male sterilization<br>3. IUD<br>4. Injectable (e.g.DMPA)<br>5. Implants<br>6. Patch<br>7. Pill<br>8. Condom<br>9. Female condom<br>10. Diaphragm<br>11. Form/Jelly/Cream<br>12. Mucus/Billings/Ovulation<br>13. Basal body temperature<br>14. Symptothermal<br>15. Standard days method<br>16. LAM<br>17. Calendar/Rhythm/Periodic abstinence<br>18. Withdrawal<br>19. Other traditional method<br>20. Other modern method (specify)		-> 401

	Section 4. Past Health facility visit and FP counseling<br>**Do not count today's visit.**							
401	Not including today, in the last 12 months, have you visited a health facility for care for yourself or your children for any purpose?<br>"Uway iyagad in adlaw ini, nakakadtu kaw pa health center tagna amun last 12 months pa healt facility atawa mga anak mo?"	1. Yes<br>2. No						1 -> 402<br>2 -> End of interview
	LINE NUMBER	01	02	03	04	05	06	
402	Now I would like to record all your facility visits for last 12 months. Start with the latest visit you had.<br>Why did you visit a health facility?<br>"Bihaun ilista ku in mga pagkadtu mo pa health center amun nakalabay 12 months. Maytah kaw miyadtu pa health facility?"<br><br>AFTER WRITING THE FIRST VISIT IN LINE NUMBER 01, ASK Q403-410 FOR THAT VISIT. THEN ASK THE 2nd LATEST VISIT TO WRITE IN 402 LINE NUMBER 02, THEN ASK Q 403 AND Q404.<br>REPEAT FOR ALL HEALTH FACILITY VISITS FOR LAST 12 MONTHS.<br>IF THERE ARE MORE THAN 6, USE AN ADDITIONAL QUESTIONNAIRE.<br><br>1. Prenatal care<br>2. Giving birth, while a women is still in the facility<br>3. Reproductive health outpatient clinic for postnatal care<br>4. Reproductive health clinic not related to postnatal care<br>5. Receiving vaccination or routine check up for child<br>6. Seeking medical advice or treatment for sickness or injury of **child**<br>7. Seeking medical advice or treatment for sickness or injury of **herself**<br>8. Adolescent clinic<br>9. Other (specify)							-> 403
403	Where did you visit?<br>"Uno in kiyadtuhan mo?"<br>1. National hospital<br>2. Regional hospital/Public medical center<br>3. Provincial hospital							-> 404

	4. District hospital<br>5. Municipal hospital<br>6. Rural health unit (RHU)/urban health center(UHC)/Lying-in<br>7. Barangay health station (BHS)<br>8. Barangay supply/service point officer/BHW<br>9. Mobile clinic<br>10. Other (specify. Private facility is included here.)							
404	At that visit, were you or your sexual partner already using any method to delay or avoid getting pregnant?<br>"Pagubus mo miyadtu. Iyusal niyo na ba magtuy in method para mag delay iban dih na maburus?"<br>1. Yes<br>2. No							1 ->405<br>2 -> 406
405	Which method(s) were you using?<br>"Uno method in iyuusal mo?"<br>WRITE DOWN ALL MENTIONED<br>1. Female sterilization<br>2. Male sterilization<br>3. IUD<br>4. Injectable (e.g.DMPA)<br>5. Implants<br>6. Patch<br>7. Pill<br>8. Condom<br>9. Female condom<br>10. Diaphragm<br>11. Form/Jelly/Cream<br>12. Mucus/Billings/Ovulation<br>13. Basal body temperature<br>14. Symptothermal<br>15. Standard days method<br>16. LAM<br>17. Calendar/Rhythm/Periodic abstinence<br>18. Withdrawal<br>19. Other traditional method<br>20. Other modern method (specify)							->406
406	At that visit, did any staff member at the health facility speak to you about family planning methods?<br>"Ha pagkadtu mo awun mga staff member ha health facility nagbaytah kaymo pasal sin family planning methods?"<br>1. Yes<br>2. No							1-> 407<br>2-> 402 next line number

407	After that visit, did you start using any FP method or change from your previous method to a new method?<br>"Pagubus sin pagkadtu mo, nagtagna na kaw nagusal family planning method atawa nag ganti kaw sin nakauna method mo pa bago?"<br>1. Yes<br>2. No							1 -> 409<br>2 ->408
408	If you did not start a new method or change from your previous method, why?<br>"Bang kaw uway nagtagna ha new method atawa nagpinda ha nakauna. Maytah?"<br>1. No need<br>2. Possible side effects of new method<br>3. New method not available at the facility<br>4. Concerns about risk of pregnancy with new method<br>5. Not enough information<br>6. Could not afford to purchase<br>7. Advice of friends, relatives, neighbours not to start or change<br>8. Husband/partner did not support<br>9. Other (specify):___________							
409	Which FP method did you start using after that visit or which new method did you change to?<br>"Uno family planning in iyusal mo pag ubus sin pagkadtu mo atawa uno in giyantian mo ha bago method?"<br>1. Female sterilization<br>2. Male sterilization<br>3. IUD<br>4. Injectable (e.g.DMPA)<br>5. Implants<br>6. Patch<br>7. Pill<br>8. Condom<br>9. Female condom<br>10. Diaphragm<br>11. Form/Jelly/Cream<br>12. Mucus/Billings/Ovulation<br>13. Basal body temperature<br>14. Symptothermal<br>15. Standard days method<br>16. LAM<br>17. Calendar/Rhythm/Periodic abstinence<br>18. Withdrawal<br>19. Other traditional method							-> 402 next line number

	20. Other modern method (specify)							

END OF THE INTERVIEW
