# Supplementary material for: Opportunities lost: Barriers to increasing the use of effective contraception in the Philippines
Source: PLoS One. 2019 Jul 25;14(7):e0218187. doi: 10.1371/journal.pone.0218187 (PMC6657820; doi:10.1371/journal.pone.0218187)
Supplement: S9 Questionnaire — (PDF) [file pone.0218187.s009.pdf]

COVER PAGE

FORM1. Iinterview women of reproductive age who are not currently pregnant or within 6 weeks of delivery, and desire delaying or limiting childbearing

Sequence number: _____________

[Fill one number for each woman contacted in the order they were contacted at the health facility; if done over several days, continue unique sequence numbers]

Identification of interview place

Region

Province

CITY/MUNICIPALITY

BARANGAY

Health facility name

Home address (for home visit only)

Latitude and longitude

(Use the coordinate of GPS in a mobile phone)

Interview Record

Date of interview

Interviewer's name

Health facility level where interview took place	1. National hospital<br>2. Regional hospital/Public medical center<br>3. Provincial hospital<br>4. District hospital<br>5. Municipal hospital<br>6. Rural health unit (RHU)/urban health center(UHC)/Lying-in

	7. Barangay health station (BHS)<br>8. Barangay supply/service point officer/BHW<br>9. Mobile clinic<br>10. Other (specify)
Clinic where interview took place (for large hospitals)	1. Postnatal health check after giving birth, after a woman left the facility<br>2. Receiving vaccination or routine check-up for child<br>3. Seeking medical advice or treatment for sickness or injury of child<br>4. Seeking medical advice or treatment for sickness or injury of **herself**<br>5. Adolescent clinic<br>6. Other (specify)

Sequence Number: _________________
[Write the same sequence number from Cover Page]
[Isulat ang parehong pagsunod-sunod sa numero gikan sa Cover Page]

Instructions:
Read the information sheet. Answer questions. If the woman agrees to participate give the certificate of consent for her to sign. Then start the Screening Form.
Basaha ang information sheet. Tubaga ang mga pangutana. Kung nisugot naang babaye muapil, ihatag ang certificate of consent para mapirmahan. Dayon sugdi na ang Screening Form.

Screening Form

State: "We would like to start by asking a few questions that determine if you are eligible for the survey."
Isulti: "Magsugod mi ug pipila ka pangutana aron mahibal-an kung angayan ba ka iapil sa survey."

001	How old were you on your last birthday?<br>*Pila'y edad nimo sa ulahi nimong adlaw nga natawhan?*	Age in completed years<br>*Edad sa kumpletong tuig*		18-49 years ->002<br>Other -> 009
002	Are you pregnant now?<br>*Nagsabak ka karon?*	*1.* Yes *Oo*<br>*2.* No *Wala*<br>*3.* Unsure *Dili sigurado*		1 ->009<br>2 ->003<br>3 ->003
003	What is the name of your last baby?<br>*Unsa'y pangalan sa imong kamanghurang anak?*<br>Record name<br>*Ilista ang mga pangalan*	1. Name: *Pangalan* _____________<br>*2.* No previous baby *Wala pa'y anak*		1 ->004<br>2 ->006
004	In what month and year was NAME born?<br>*Unsang bulana ug tuig natawo si PANGALAN?*<br>(probe: when is his or her birthday)<br>*(probe: kanus'a iyang birthday?)*	Month: __ __<br>*Buwan*<br>Year: __ __ __ __<br>*Tuig*		Age ≥6 wks ->005<br>*Edad > 6 semana*<br>Age<6 wks ->009<br>*Edad < 6 semana*
005	Has your menstrual period returned since the birth of NAME?<br>*Gidugo na ba ka ug balik gikan*	*1.* Yes *Oo*<br>*2.* No *Wala*		1 ->006<br>2 ->006

	pagpanganak kang PANGALAN?			
006	Now I have some questions about the future. Would you like to have (a/another) child, or would you prefer not to have any (more) children?<br><br>*Karon naa ko'y pangutana kabahin sa kaugmaon. Ganahan pa ka pun-an imong anak o dili na ka ganahan pun-an?*	*1.* Have (a/another) child *Pun-an pa ang anak*<br>*2.* No more/none *Dili na*<br>*3.* Cannot get pregnant *Dili na maburos*<br>*4.* Undecided / don't know *Wala pa kabalo*		1 -> 007<br><br>2 ->008<br><br>3 ->009<br><br>4 ->009
007	Do you want (a/another) child soon?<br><br>*Sa dili madugay, gusto pa ka ug laing anak?*	*1.* Yes *Oo*<br>*2.* No, want to wait *Dili, maghulat ko*<br>*3.* Don't know *Wala ko kabalo*		1 ->009<br>2 ->008<br>3- >009
008	Are you or your husband/partner currently doing something or using any method to delay or avoid getting pregnant?<br><br>*Ikaw ba o ang imong kapikas adunay gigamit nga mga pamaagi aron mapugngan o malikayan nga magsabak ka?*	*1.* Yes *Oo*<br><br>*2.* No *Wala*		1 -> 101<br><br>2 -> 101<br><br>To achieve a total of 5 users and non-users (hospitals) and 3 users and 3 non-users (health centres).<br><br>*Para makakuha sa kinatibuk-an nga 5 ka naggamit ug wala naggamit (hospitals) ug 3 ka naggamit ug 3 ka wala naggamit (health centers)*
009	Thank the woman, indicate ineligibility for the survey and stop the interview. Enter this woman into "number of women contacted". Then find another woman to interview.<br><br>*Pasalamati ang babaye nga nagpakita nga dili siya pwede iapil sa survey ug hununga na ang pag interbyu. Iapil ning bayhana sa "numbers of women contacted". Dayon pangita ug laing babaye para mainterbyu.*			

QUESTIONNAIRE

FORM1. Interview of women of reproductive age who are not currently pregnant or within 6 weeks of delivery, and desire delaying or limiting childbearing
Interbyu sa mga babaye nga anaa sa reproductive age nga wala magsabak o manganakay na sulod sa unom ka semana ug nagtinguha nga mapugngan o paglimit sa pagpanganak.

Sequence Number:
[Write the same sequence number from Cover Page]

[Isulat ang parehong pagsunod-sunod sa numero gikan sa Cover Page]

NO.	Section 1. Respondent background			
101	In (month of interview) 2016, did you live in a city, in a town proper/ poblacion, in the barrio or rural area, or abroad?<br><br>*Sa (bulan nga nag-interbyu) 2016, nagpuyo ba ka sa syudad, sentro/ poblacion, sa bukid, o sa laing nasud?*	1. City *Syudad*<br>2. TOWN PROPERIPOBLACION *Sentro/ Poblacion*<br>3. BARRIO/RURAL AREA *Bukid*<br>4. ABROAD *Laing nasud*<br>5. DON'T KNOW *Wala kabalo*		->102
102	What is your marital status now?<br><br>*Unsa man ang estado sa imong kaminyoon karon?*	1. Never married or never lived with a man *Wala pa maminyo o wala pa makig-ipon sa lalaki*<br>2. Currently married *Kasamtangang Minyo*<br>3. Currently living with a man *Kasamtangang naa'y kaipon nga lalaki*<br>4. Divorced/separated/widow and not currently living with a man *deborsyada/bulag/balo ug walay kaipon nga lalaki*		-> 103

103	What is your highest level of education attended, whether or not that level was completed?<br><br>*Unsa man ang pinakataas nga lebel nga imong natunghaan sa pag-eskwela, nahuman man nimo o wala?*	*1.* No education *Wala naka-eskwela*<br><br>*2.* Elementary *Elementarya*<br><br>3. High school Hayskul<br><br>*4.* College *Kolehiyo*<br><br>5. Post-graduate		->104
104	How many children do you have who are still alive?<br><br>*Pila kabuok imong mga anak nga buhi pa?*	Number of children alive<br><br>*Gidaghanon sa anak nga buhi*		->106
105	Did you or someone else do anything to end any of your past pregnancies?<br><br>*Sa mga niaging pagsabak nimo, nakapakuha na ba ka o kaha adunay laing tawo nga nagpakuha sa imong gisabak?*	*1.* Yes *Oo*<br><br>*2.* No *Wala*		1 ->107<br><br>2 -> 108
106	How many pregnancies did you or someone else do anything to end?<br><br>*Kapila ka o kaha laing tawo nakapakuha sa imong gisabak?*	Number of induced abortion<br><br>*Gidagahanon sa abosyon*		->108
107	Are you covered by any health insurance, either as member or dependent?<br><br>*Aduna ka ba'y health insurance, kanang membro o gadepende ka?*	1. Not covered<br><br>2. Philhealth<br><br>3. Government Service Insurance System<br><br>4. Social Security System<br><br>5. Private insurance company/Health maintenance organization /Pre-need insurance plan		->201

		company<br>6. Other (Specify) *Uban pa (Hinganli)*		

NO.

Section 2. Current use of FP

201	REVIEW: Are you or your husband/partner currently doing something or using any method to delay or avoid getting pregnant?<br><br>I*kaw ba o ang imong bana/ kapikas naggamitan ug bisan unsang pamaagi aron mapugngan o malikayan ang pagsabak?*	3. Yes *Oo*<br>4. No *Wala*		1 -> 202<br>2 -> 206
202	Which method are you currently using?<br><br>*Unsa nga pamaagi ang gigamit nimo karon?*<br><br>WRITE DOWN ALL MENTIONED.<br><br>*ILISTA ANG TANANG GILITOK*	1. Female sterilization<br>2. Male sterilization<br>3. IUD<br>4. Injectable (e.g.DMPA)<br>5. Implants<br>6. Patch<br>7. Pill<br>8. Condom<br>9. Female condom<br>10. Diaphragm<br>11. Form/Jelly/Cream<br>12. Mucus/Billings/Ovulation<br>13. Basal body temperature<br>14. Symptothermal<br>15. Standard days method<br>16. LAM<br>17. Calendar/Rhythm/Periodic abstinence<br>18. Withdrawal<br>19. Other traditional method *Uban pang kinaraan nga*		-> 203

		pamaagi<br>20. Other modern method (specify) *Uban pang modernong pamaagi (Hinganli)*					
	LINE NUMBER	01	02	03	04	05	
203	Now I would like to ask you one by one about all methods you are using now.<br><br>*Pangutan-on ka nako karon matag usa kabahin sa tanang pamaagi nga kasamtangan nimong gigamit.*<br><br>RECORD ALL METHODS BEING USED NOW, ONE METHOD PER ONE LINE NUMBER.<br><br>*ILISTA TANANG PAMAAGI NGA KASAMTANGANG GIGAMIT. USA KA PAMAAGI KADA LINYA SA NUMERO.*<br><br>IF THERE ARE MORE THAN 5 METHODS, USE ADDITIONAL QUESTIONNAIRE.<br><br>*KUNG LABAW PA SA 5 KA PAMAAGI, PAGGAMIT UG ADDITIONAL QUESTIONNAIRE*<br><br>1. Female sterilization<br>2. Male sterilization<br>3. IUD<br>4. Injectable (e.g.DMPA)<br>5. Implants<br>6. Patch<br>7. Pill<br>8. Condom<br>9. Female condom<br>10. Diaphragm<br>11. Form/Jelly/Cream						->204

	12. Mucus/Billings/Ovulation<br>13. Basal body temperature<br>14. Symptothermal<br>15. Standard days method<br>16. LAM<br>17. Calendar/Rhythm/Periodic abstinence<br>18. Withdrawal<br>*19.* Other traditional method *Uban pang kinaraang pamaagi*<br>*20.* Other modern method (specify) *Uban pang modernong pamaagi (Hinganli)*						
204	Where did you obtain that method when you first started using it?<br><br>*Sa pagsugod nimo ug gamit, diin man ka nakahibalo ana nga pamaagi?*<br><br>1. National hospital<br>2. Regional hospital/Public medical center<br>3. Provincial hospital<br>4. District hospital<br>5. Municipal hospital<br>6. Rural health unit (RHU)/urban health center(UHC)/Lying-in<br>7. Barangay health station (BHS)<br>8. Barangay supply/service point officer/BHW<br>9. Mobile clinic<br>*10.* Other (specify. Private facility is included here) *Uban pa (Hinganli. Apil diri*						-> 205

	ang Private Facility)						
205	What was the purpose of your going to the health facility on the day you first received the contraceptive method?<br><br>*Unsa man ang imong tumong sa pag-adto sa health facility niadtong unang adlaw nga nakadawat ka sa contraceptive method?*<br><br>1. Prenatal care<br>*2.* Giving birth, while a women is still in the facility *Nanganak, samtang anaa pa sulod sa facility.*<br>*3.* Health check after giving birth, after a woman left the facility. *Nagpakonsulta human nanganak, sa dihang nibiya na sa facility*<br>*4.* Receiving vaccination or routine check up for child. *Nagpabakuna o naandan ng check up sa bata.*<br>*5.* Seeking medical advice or treatment for sickness or injury of **child.** *Nagpakonsulta o nagpatambal sa sakit o samad sa bata*<br>*6.* Seeking medical advice or treatment for sickness or injury of **herself.** *Nagpakonsulta o nagpatambal sa sakit o samad sa kaugalingon.*<br>7. Adolescent clinic<br>*8.* Other (specify) *Uban pa*						-> 203. Repeat until all methods were explained.<br><br>*Usba taman mahuman na ug pagpasabot ang tanang mga pamaagi.*<br><br>Then ->206

	(Hinganli)						

206	If you <u>are not</u> using any method to delay or avoid getting pregnant now, have you or your sexual partner done something or used a method to delay or avoid getting pregnant in the past?<br><br>*Kung karon wala kay gigamit nga bisan unsang pamaagi aron mapugngan o malikayan ang pagsabak, naggamit ka ba sauna o ang imong kapikas ug mga pamaagi aron mapugngan o malikayan ang pagsabak?*<br><br>If <u>you are</u> using a method to delay or avoid getting pregnant now, have you or your sexual partner ever used a different method to delay or avoid getting pregnant in the past?<br><br>*Kung kasamtangan kang naggamit ug pamaagi aron mapugngan o malikayan ang pagsabak, naggamit ba ka sauna o ang imong kapikas ug laing pamaagi aron mapugngan o malikayan ang pagsabak?*	*1.* Yes *Oo*<br><br>*2.* No *Wala*		1->207<br><br>2-> 301
207	Which methods have you used in the past?<br><br>*Unsa nga mgapamaagi ang gigamit nimo sauna?*	1. Female sterilization<br>2. Male sterilization<br>3. IUD<br>4. Injectable (e.g.DMPA)<br>5. Implants<br>6. Patch<br>7. Pill<br>8. Condom<br>9. Female condom		-> 208

	WRITE DOWN ALL MENTIONED.<br><br>*ILISTA ANG TANANG GILITOK*	10. Diaphragm<br>11. Form/Jelly/Cream<br>12. Mucus/Billings/Ovulation<br>13. Basal body temperature<br>14. Symptothermal<br>15. Standard days method<br>16. LAM<br>17. Calendar/Rhythm/Periodic abstinence<br>18. Withdrawal<br>*19.* Other traditional method *Uban pang kinaraang pamaagi*<br>*20.* Other modern method (specify) U*ban pang modernong pamaagi (Hinganli)*					
	LINE NUMBER	01	02	03	04	05	
208	Now I would like to ask you one by one about all methods you have used in the past<br><br>*Pangutan-on ka nako karon matag usa sa mga pamaaging gigamit nimo sauna*<br><br>RECORD ALL METHODS, ONE METHOD PER ONE LINE NUMBER.<br><br>*ILISTA TANANG PAMAAGI. USA KA PAMAAGI KADA LINYA SA NUMERO.*<br><br>IF THERE ARE MORE THAN 5 METHODS, USE ADDITIONAL QUESTIONNAIRE.<br><br>*KUNG LABAW PA SA 5 KA PAMAAGI, PAGGAMIT UG ADDITIONAL QUESTIONNAIRE*<br><br>1. Female sterilization<br>2. Male sterilization						->209

	3. IUD<br>4. Injectable (e.g.DMPA)<br>5. Implants<br>6. Patch<br>7. Pill<br>8. Condom<br>9. Female condom<br>10. Diaphragm<br>11. Form/Jelly/Cream<br>12. Mucus/Billings/Ovulation<br>13. Basal body temperature<br>14. Symptothermal<br>15. Standard days method<br>16. LAM<br>17. Calendar/Rhythm/Periodic abstinence<br>18. Withdrawal<br>*19.* Other traditional method *Uban pangkinaraang pamaagi*<br>*20.* Other modern method (specify) *Uban pang modernong pamaagi (Hinganli)*						
209	Where did you obtain the family planning method when you first started using it?<br><br>*Sa pagsugod nimo ug gamit, diin man ka nakat-on sa family planning method?*<br><br>1. National hospital<br>2. Regional hospital/Public medical center<br>3. Provincial hospital<br>4. District hospital<br>5. Municipal hospital<br>6. Rural health unit (RHU)/urban health center(UHC)/Lying-in<br>7. Barangay health station (BHS)						->210

	8. Barangay supply/service point officer/BHW<br>9. Mobile clinic<br>*10.*Other (specify. Private facility is included here.) *Uban pa (Hinganli. Apil diri ang Private Facility)*						
210	Why did you visit the health facility where you first started using the family planning method?<br><br>*Nganong nibisita man ka sa health facility niadtong unang gamit nimo sa family planning method?*<br><br>1. Prenatal care<br>*2.* Giving birth, while still in the facility. N*anganak, samtang anaa pa sulod sa facility.*<br>*3.* Health check after giving birth, after leaving the facility. *Nagpakonsulta human nanganak, sa dihang nibiya na sa facility*<br>4. Receiving vaccinations or routine check-ups for a child. N*agpabakuna o naandan ng check up sa bata.*<br>*5.* Seeking medical advice or treatment for sickness or injury of a **child.** *Nagpakonsulta o nagpatambal sa sakit o samad sa bata*<br>*6.* Seeking medical advice or treatment for sickness or injury of **herself.** *Nagpakonsulta o nagpatambal sa sakit o samad sa kaugalingon.*<br>7. Adolescent clinic<br>*8.* Other (specify) *Uban pa (Hinganli)*						-> 211

211	Why did you stop using the family planning method that you used in the past?<br><br>*Nganong niundang ka sauna sa naandan nimong family planning method?*<br><br>*1.* Side effects *Dili maayong epekto*<br>*2.* Method not available at the facility. *Pamaagi wala sa facility*<br>*3.* Concerns about risks of pregnancy. *Mahadlok sa risgo nga magsabak.*<br>*4.* Could not afford to purchase. *Walay ikapalit*<br>*5.* Health worker did not continue to provide the method. N*aundangan sa health worker ug hatag ana nga pamaagi*<br>*6.* Advice of friends, relatives, neighbors. *Sugyot sa mga higala, mga paryente, mga silingan.*<br>*7.* Husband/partner did not support. *Dili suportado sa bana/ kapikas.*<br>*8.* Wanted to get pregnant. *Gustong magsabak*<br>*9.* Other (specify):__________ *Uban pa (Hinganli)*						->212

	**Section 3. FP Concerns and Today's FP counseling**						
301	Do you have any health concerns about any type of family planning method?<br>*Aduna ka ba'y kabalaka sa bisan unsang family planning method?*	*1.* Yes *Oo*<br>*2.* No *Wala*					1 ->302<br>2 ->305
	LINE NUMBER	01	02	03	04	05	06	
302	What are your health concerns about family planning methods?<br>*Unsa ang imong kabalaka sa*							-> 303

	family planning methods?<br>Please tell me one by one.<br>*Palihog isulti sa akoa tagsa-tagsa*<br><br>USE ONE LINE NUMBER FOR ONE CONCERN. WRITE DOWN ALL MENTIONED CONCERNS.<br>*GAMITA ANG USA KA LINYA SA NUMERO KADA USA KA KABALAKA. ILISTA TANANG NALITOK NGA KABALAKA.*<br>IF THERE ARE MORE THAN 6 CONCERNS, USE ADDITIONAL QUESTIONNAIRE.<br>*KUNG LABAW PA SA 6 KA KABALAKA, PAGGAMIT UG ADDITIONAL QUESTIONNAIRE*<br><br>*1.* Cause cancer in the uterus. H*inungdan sa cancer sa matres*<br>*2.* Cause cysts in the uterus. *Hinungdan sa cysts sa matres.*<br>*3.* Cause infection of the uterus. *Hinungdan sa pagnana sa matres.*<br>*4.* Cause frequent bleeding. H*inungdan sa makanunayong pagdugo.*<br>*5.* Cause thyroid problems. *Hinungdan sa problema sa bugabuga.*<br>*6.* Cause/worse asthma. *Hinungdan/ misamot ang hubak.*<br>*7.* Cause/worse lots of veins. *Hinungdan/ misamot ang barikos.*<br>*8.* Cause dry skin, skin disease. *Hinungdan nga nag-uga ang pamanit, nakaangkon ug mga sakit-sakit sa pamanit.*<br>9. Cause edema. *Hinungdan sa mga hubag sa kalawasan.*<br>*10.* Cause weight gain.							

	Hinungdan nga nanambok.<br>*11.* Cause weight loss.<br>*Hinungdan nga nagniwang*<br>*12.* Cause bloated stomach.<br>*Hinungdan nga niburot ang tiyan.*<br>*13.* Cause headach.<br>*Hinungdan sa labad sa ulo*<br>*14.* Cause irritability.<br>*Hinungdan sa pagkasapoton*<br>*15.* Increase libido/turn into a maniac. *Hinungdan sa pagtaas sa gana o panginahanglan sa pakipaghilawas*<br>*16.* Cause loss/reduce of libido. *Hinungdan sa pagkawala sa gana sa pakipaghilawas*<br>*17.* Cause loss/reduce of sexual satisfaction. *Hinungdan sa pagkawala sa katagbawan sa pakipaghilawas.*<br>*18.* One will not have children anymore. *Dili na makaanak*<br>*19.* Not fully effective, woman could still get pregnant. *Dili epektibo, nagsabak lang gihapon*<br>*20.* When it does not work, the baby is born with abnormalities. *Sa panahong wala niepekto, gihimugso ang bata nga naa'y apan/ abnormalidad*<br>*21.* Results in mortal sin because it is against church teachings. *Dakong sala kay supak sa mga pagtulon-an sa simbahan.*<br><br>**IUD/Implants**<br>*22.* Melt or move around inside the body and doctors will not be able to find. *Nalanay o nagbalhin-balhin sulod sa lawas ug dili na*							

	makit-an sa doktor<br>*23.* Washed away/pushed out of body. *Nabanwas/ nigawas sa lawas*<br>*24.* Painful to insert. *Sakit itaud*<br><br>**IUD**<br>*25.* Itchy on the vagina. *Katol sa kinatawhan sa baye.*<br>*26.* Entangled around the man's penis. *Nasangit sa kinatawhan sa laki.*<br>*27.* Messy when inserted. *Hugawan nga nataud*<br><br>**Male sterilization**<br>*28.* Part of the man's testicles are cut off. *Giputlan ang parte sa itlog sa laki.*<br>*29.* It hurts the testicles. *Sakit sa itlog*<br>*30.* The man loses his manhood ("kapon"). *Kaponon ang laki*<br>*31.* Others (specify) *Uban pa (Hinganli)*							
303	About which family planning methods do you have concerns? *Sa unsang family planning method nabalaka ka?*<br><br>REPEAT EACH CONCERN IN TURN. FOR EACH CONCERN, WRITE DOWN ALL METHODS CAUSING THAT CONCERN. *USBA ANG MATAG KABALAKA NGA NALITOK. KADA KABALAKA ILISTA ANG TANAG PAMAAGI NGA HINUNGDAN SA KABALAKA.*<br><br>1. Female sterilization<br>2. Male sterilization<br>3. IUD<br>4. Injectable							-> 304

	5. Implants<br>6. Patch<br>7. Pill<br>8. Other modern method (specify)<br>*9.* Other method (specify) *Uban pang pamaagi (Hinganli)*							
304	Who told you or how did you find about your concerns about family planning methods?<br>*Kinsa'y gasulti o giunsa nimo pagkahibalo anang imong kabalaka kabahin sa family planning methods?*<br><br>REPEAT EACH CONCERN IN TURN. FOR EACH WRITE DOWN ALL SOUCES OF INFORMATION.<br>*USBA KADA KABALAKANG NALITOK. KADA KABALAKA ILISTA TANANG TINUBDAN SA INPORMASYON.*<br><br>1. Health staff<br>2. BHW or health volunteers<br>*3.* Husband or partner *Bana o kapikas*<br>*4.* Friend, neighbours, relatives *Higala, mga silingan, mga paryente*<br>*5.* Church *Simbahan*<br>*6.* Radio *Radyo*<br>7. Television<br>8. Newspaper or magazine<br>9. Online or internet<br>*10.* Others (specify) *Uban pa (Hinganli)*							-> 305
305	Today, did any staff member at the health facility speak to you about family planning methods?<br>*Pagkakaron, aduna ba'y trabahante sa health facility nga naghisgot sa imoha kabahin sa family planning methods.*	*1.* Yes *oo*<br>*2.* No *wala*						1 ->306<br>2 ->401

306	Did the health worker ask you about your concerns?<br>*Gipangutana ba ka sa health worker kabahin sa imong mga kabalaka?*	*1.* Yes *Oo*<br>*2.* No *Wala*		1 ->307<br>2 -> 309
307	Do you feel the health worker understands your concerns?<br>*Nbati ba nimo nga nasabtan sa health worker ang imong mga kabalaka?*	*1.* Yes *Oo*<br>*2.* No *Wala*		->308
308	Did the health worker help you to find solutions to your concerns?<br>*Nitabang ba ang health worker ug pangita'g solusyon sa imong mga kabalaka?*	*1.* Yes *Oo*<br>*2.* No *Wala*		->309
309	Did the health worker offer you information how different family planning methods work?<br>*Gipasabot ba sa health worker ang epekto sa nagkalain-laing family planning methods?*	*1.* Yes *Oo*<br>*2.* No *Wala*		1 ->310<br>2 ->312
310	Which methods did health worker mention today?<br>*Unsa nga mga pamaagi ang nahisgutan sa heath worker karong adlawa?*	1. Female sterilization<br>2. Male sterilization<br>3. IUD<br>4. Injectable (e.g.DMPA)<br>5. Implants<br>6. Patch<br>7. Pill<br>8. Condom<br>9. Female condom<br>10. Diaphragm<br>11. Form/Jelly/Cream<br>12. Mucus/Billings/Ovulation<br>13. Basal body temperature<br>14. Symptothermal<br>15. Standard days method<br>16. LAM<br>17. Calendar/Rhythm/Periodic abstinence<br>18. Withdrawal		- >311

		19. Other traditional method *Uban pang kinaraan nga pamaagi*<br>20. Other modern method (specify) *Uban pang modernong pamaagi (Hinganli)*		
311	Did the health worker tell you about side-effects or problems you might have with any methods of family planning? *Anaa ba'y nahisgutan ang health worker kabahin sa mga makadaot nga epekto o problema nga mahiaguman sa bisan unsang family planning methods?*	1. Yes *Oo*<br>2. No *Wala*		-> 312
312	Did the health worker offer you information how your family planning method works? *Gihatagan ba ka ug inpormasyon sa health worker kung unsa'y nabuhat sa imong family planning method?*	1. Yes *Oo*<br>2. No *Wala*<br>3. N/A (not using a method now) *(wala'y gigamit karon)*		1 -> 313<br>2-> 313<br>3-> 315
313	Did the health worker explain about the side effects of your current method?<br><br>*Gipasabot ba sa health worker ang mga makadaot nga epekto sa gigamit nimo nga pamaagi karon?*	1. Yes *Oo*<br>2. No *Wala*		-> 314
314	Did the health worker ask you to describe how you use your current method?<br><br>*Gipasaysay ba sa health worker kung giunsa nimo paggamit ang imong pamaagi karon?*	1. Yes *Oo*<br>2. No *Wala*		-> 401
315	After receiving FP counselling will you begin using a family planning method today?	1. Yes *Oo*<br>2. No *Dili*		1 -> 317<br>2 -> 316

	Pagkahuman sa family planning counseling, magsugod na ba ka ug gamit sa family planning methods?			
316	After receiving FP counselling will you begin using, do you think you will use a contraceptive method anytime in the future?<br>*Pagkahuman sa family planning counseling, kursonada na ba ka mugamit sa contraceptive method bisan unsang orasa sa mga umaabot nga mga adlaw?*	*1.* Yes *Oo*<br>*2.* No *Dili*		1-> 317<br>2-> 401
317	Which contraceptive method would you prefer to use?<br><br>*Unsa nga contraceptive method ang gusto nimo gamiton?*	1. Female sterilization<br>2. Male sterilization<br>3. IUD<br>4. Injectable (e.g.DMPA)<br>5. Implants<br>6. Patch<br>7. Pill<br>8. Condom<br>9. Female condom<br>10. Diaphragm<br>11. Form/Jelly/Cream<br>12. Mucus/Billings/Ovulation<br>13. Basal body temperature<br>14. Symptothermal<br>15. Standard days method<br>16. LAM<br>17. Calendar/Rhythm/Periodic abstinence<br>18. Withdrawal<br>19. Other traditional method *Uban pang kinaraang pamaagi*<br>*20.* Other modern method (specify) *Uban pang modernong pamaagi (Hinganli)*		-> 401

<table>
<tr><td></td><td colspan="8">Section 4. Past Health facility visit and FP counseling

Do not count today's visit.
Ayaw apila ug ihap ang pagbisita karong adlawa.</td></tr>
<tr><td>401</td><td>Not including today, in the last 12 months, have you visited a health facility for care for yourself or your children for any purpose?

Wala'y labot karon, sa mga nilabay nga 12 ka bulan nibisita ba ka sa health facility aron pag-atiman sa imong kaugalingon o sa imong mga anak sa bisan unsang katuyoan?</td><td colspan="4">1. Yes Oo
2. No Wala</td><td colspan="2"></td><td>1 -> 402
2 -> End of interview</td></tr>
<tr><td></td><td>LINE NUMBER</td><td>01</td><td>02</td><td>03</td><td>04</td><td>05</td><td>06</td><td></td></tr>
<tr><td>402</td><td>Now I would like to record all your facility visits for last 12 months. Start with the latest visit you had.

Ilista nako karon tanang pagbisita nimo sa facility sa mga nilabay nga 12 ka bulan. Sugdi sa pinakaulahian.

Why did you visit a health facility?

Nganong nibisita man ka sa health facility?

AFTER WRITING THE FIRST VISIT IN LINE NUMBER 01, ASK Q403-410 FOR THAT VISIT. THEN ASK THE 2nd LATEST VISIT TO WRITE IN 402 LINE NUMBER 02, THEN ASK Q 403 AND Q404.</td><td></td><td></td><td></td><td></td><td></td><td></td><td>-> 403</td></tr>
</table>

	PAGKAHUMAN UG SULAT SA UNANG BISITA SA NUMERO 01 NGA LINYA, IPANGUTANA ANG Q403-410 PARA ANA NGA PAGBISITA. DAYON PANGUTAN-A ANG IKADUHANG PINAKAULAHING PAGBISITA PARA ISULAT SA 402 NUMERO 02 NGA LINYA, DAYON IPANGUTANA ANG Q403 UG Q404<br><br>REPEAT FOR ALL HEALTH FACILITY VISITS FOR LAST 12 MONTHS.<br><br>*USBA PARA SA TANANG PAGBISITA SA HEALTH FACILITY SA MGA NILABAY NGA 12 KA MGA BULAN.*<br><br>IF THERE ARE MORE THAN 6, USE AN ADDITIONAL QUESTIONNAIRE.<br><br>*KUNG LABAW PA SA 6, PAGGAMIT UG ADDITIONAL QUESTIONNAIRE.*<br><br>1. Prenatal care<br>2. Giving birth, while a women is still in the facility *Nanganak, samtang anaa pa sulod sa facility.*<br>3. Health check after giving birth, after a woman left the facility *Nagpakonsulta human nanganak, sa dihang nibiya na sa facility.*<br>4. Receiving vaccination or routine check up for child *Nagpabakuna o naandan ng*							

	check-up sa bata.<br>*5.* Seeking medical advice or treatment for sickness or injury of **child** *Nagpakonsulta o nagpatambal sa sakit o samad sa bata.*<br>*6.* Seeking medical advice or treatment for sickness or injury of **herself** *Nagpakonsulta o nagpatambal sa sakit o samad sa imong kaugalingon.*<br>7. Adolescent clinic<br>*8.* Other (specify) *Uban pa (Hinganli)*							
403	Where did you visit?<br><br>*Asa ka nibisita?*<br><br>1. National hospital<br>2. Regional hospital/Public medical center<br>3. Provincial hospital<br>4. District hospital<br>5. Municipal hospital<br>6. Rural health unit (RHU)/urban health center(UHC)/Lying-in<br>7. Barangay health station (BHS)<br>8. Barangay supply/service point officer/BHW<br>9. Mobile clinic<br>*10.* Other (specify. Private facility is included here.) *Uban pa (Hinganli. Apil diri ang Private facility)*							-> 404
404	At that visit, were you or your							1 ->405

	sexual partner already using any method to delay or avoid getting pregnant?<br><br>*Anang pagbisitaha, ikaw ba o ang imong kapikas aduna na'y gigamit nga bisan unsang pamaagi aron mapugngan o malikayan ang pagsabak?*<br><br>*1.* Yes *Oo*<br>*2.* No *Wala*							2 -> 406
405	Which method(s) were you using?<br><br>*Unsa nga pamaagi ang inyong gigamit?*<br><br>WRITE DOWN ALL MENTIONED<br>*ISULAT ANG TANANG GILITOK.*<br><br>1. Female sterilization<br>2. Male sterilization<br>3. IUD<br>4. Injectable (e.g.DMPA)<br>5. Implants<br>6. Patch<br>7. Pill<br>8. Condom<br>9. Female condom<br>10. Diaphragm<br>11. Form/Jelly/Cream<br>12. Mucus/Billings/Ovulation<br>13. Basal body temperature<br>14. Symptothermal<br>15. Standard days method<br>16. LAM<br>17. Calendar/Rhythm/Periodic abstinence<br>18. Withdrawal<br>19. Other traditional method *Uban pang kinaraang pamaagi*							->406

	20. Other modern method (specify) *Uban pang modernong pamaagi (Hinganli)*							
406	At that visit, did any staff member at the health facility speak to you about family planning methods?<br><br>*Anang pagbisitaha, aduna ba'y trabahante sa health facility ang nagsulti sa imoha kabahin sa mga pamaagi sa family planning?*<br><br>*1.* Yes *Oo*<br>*2.* No *Wala*							1-> 407<br><br>2-> 402 next line number
407	After that visit, did you start using any FP method or change from your previous method to a new method?<br><br>*Pagkahuman ug bisita, nagsugod na ba ka dayon ug gamit sa bisan unsang family planning method o kaha nag-ilis ka sa nahiunang pamaagi ngadto sa bag-ong pamaagi?*<br><br>*1.* Yes *Oo*<br>*2.* No *Wala*							1 -> 409<br><br>2 ->408
408	If you did not start a new method or change from your previous method, why?<br><br>*Kung wala ka nagsugod ug gamit sa bag-ong pamaagi o nag-ilis gikan sa nahiunang pamaagi, ngano man?*<br><br>*1.* No need *Dili na kinahanglanon*<br><br>*2.* Possible side effects of new method *Posibli nga*							

	makadaot nga epekto sa bag-ong pamaagi<br>*3.* New method not available at the facility *Wala sa facility ang bag-ong pamaagi*<br>*4.* Concerns about risk of pregnancy with new method *Mahadlok sa risgo nga magsabak sa bag-ong pamaagi*<br>*5.* Not enough information *Kulang sa inpormasyon*<br>*6.* Could not afford to purchase *Wala'y ikapalit*<br>*7.* Advice of friends, relatives, neighbours not to start or change *Sugyot sa mga higala, mga paryente, mga silingan nga wala nibalhin o nagsugod ug gamit sa bag-ong pamaagi*<br>*8.* Husband/partner did not support *Dili suportado sa bana/ kapikas*<br>*9.* Other (specify):__________ *Uban pa (Hinganli)*							
409	Which FP method did you start using after that visit or which new method did you change to?<br>*Unsa nga family planning method ang gisugdan nimo ug gamit pagkahuman nimo ug bisita o unsa nga bag-ong pamaagi ang imong giilis?*<br>1. Female sterilization<br>2. Male sterilization<br>3. IUD							-> 402 next line number

	4. Injectable (e.g.DMPA)<br>5. Implants<br>6. Patch<br>7. Pill<br>8. Condom<br>9. Female condom<br>10. Diaphragm<br>11. Form/Jelly/Cream<br>12. Mucus/Billings/Ovulation<br>13. Basal body temperature<br>14. Symptothermal<br>15. Standard days method<br>16. LAM<br>17. Calendar/Rhythm/Periodic abstinence<br>18. Withdrawal<br>19. Other traditional method *Uban pang kinaraang pamaagi*<br>*20.* Other modern method (specify) *Uban pang modernong pamaagi (Hinganli)*							

END OF THE INTERVIEW
