# Supplementary material for: Opportunities lost: Barriers to increasing the use of effective contraception in the Philippines
Source: PLoS One. 2019 Jul 25;14(7):e0218187. doi: 10.1371/journal.pone.0218187 (PMC6657820; doi:10.1371/journal.pone.0218187)
Supplement: S10 Questionnaire — (PDF) [file pone.0218187.s010.pdf]

FACILITY NUMBER:

| | | | | |
|---|---|---|---|---|
| | | | | |
| Region | Province | City/Mun | Brgy | No |

As of April 14, 2017

FORM2. **Assessments of Facility Availability of Family Planning Commodities and Hospital Accreditation Status**

[Fill one number for each health facility where the interview of the woman was conducted.]

| Identification of the Health Facility | | |
|---|---|---|
| Region | | |
| Province | | |
| City/Municipality | | |
| Health facility name | | |
| Barangay | | |
| Latitude and longitude (Use the coordinate of GPS in a mobile phone) | Latitude | Longitude |
| Interview Record | | |
| Date of Interview **(dd/mm/yyyy)** | | |
| Name of Key Informant (**Last Name, Fist Name Middle Initial)** | | |
| Type of Health facility | | |
| | **Public Sector**<br>1. Government hospital<br>2. Rural health unit/Urban health center /<br>Barangay Health center<br>3. Barangay health station<br>4. Barangay supply/Service point office/BHW<br>5. Other public (specify)<br>**Other places**<br>6. Puericulture center<br>7. Others (specify) _____________ | |

FACILITY NUMBER:

| | | | | |
|---|---|---|---|---|
| | | | | |
| Region | Province | City/Mun | Brgy | No |

Section 1. Accreditations and certifications of health facility.
**[ANSWER ALL QUESTIONS. TAKE A PICTURE OF THE EVIDENCE AND INSERT TO THE RIGHT]**

| | Item | | Write the response here | Insert Picture |
|---|---|---|---|---|
| 101 | Phil Health Accredited | 1 – Yes<br>2 - No | | |
| 102 | Philippine Obstetrics and Gynecology Society Accredited | 1 – Yes<br>2 - No | | |
| 103 | At least one staff certified on implant insertion | 1 – Yes<br>2 - No | | |
| 104 | Any other accreditation status? | Specify___________ | | |

| Presence of Regular Health Personnel | 1-Yes 2-No |
|---|---|
| Doctor | |
| Dentist | |
| Nurse | |
| Midwife | |

FACILITY NUMBER:

| Region | Province | City/Mun | Brgy | No |
|---|---|---|---|---|

Section2. Commodity stocks and condition during past 12 months

**[ANSWER ALL QUESTIONS]**

| | Commodity | Do you regularly provide/have any of the commodity listed below?[a] 1-Yes 2-No | Are these available on the day of the visit? 1-Yes 2-No | Are stocks stored in normal condition[b]? 1-Yes 2-No | Are there expired commodity?/ Are the equipment functional? 1-Yes 2-No | Are there stock records? 1-Yes 2-No | In 2016, how many times in a month that you did not have any stock? [c] | Price (Pesos) (NO NEED TO FILL-OUT THOSE ITEMS WHICH THE HOSPITAL DOES NOT HAVE) | |
|---|---|---|---|---|---|---|---|---|---|
| 201 | Condom | | | | | | | | /per piece |
| 202 | Pill | | | | | | | | /per sheet |
| 203 | Patch | | | | | | | | /per piece |
| 204 | Injectable (e.g., Depo-Provera-DMPA | | | | | | | | /per kit |
| 205 | Implants | | | | | | | | /per kit |
| 206 | IUD | | | | | | | | /per kit |
| 207 | Equipment for Male sterilization | | | | | | | | /per operation |
| 208 | Equipment for Female sterilization | | | | | | | | /per operation |

[a] If the answer is no, there is no need to ask the columns to the right. Go to the next commodity.

[b] Clean, dry storage, away from direct sun and protected from extreme heat. (Family Planning. A global handbook for providers WHO 2011) [INTERVIEWER MUST SEE WHERE THE COMMODITY IS STORED AND IF THERE ARE EXPIRED COMMODITY]

c How many times in 2016 (January 2016 – December 2016) was the facility without any stock available (even if for one day only) = How many months was the stock not available for at least 1 day (x/12).
